# Mitochondrial DNA damage triggers spread of Parkinson’s disease-like pathology

**DOI:** 10.1038/s41380-023-02251-4

**Published:** 2023-10-02

**Authors:** Emilie Tresse, Joana Marturia-Navarro, Wei Qi Guinevere Sew, Marina Cisquella-Serra, Elham Jaberi, Lluis Riera-Ponsati, Natasha Fauerby, Erling Hu, Oliver Kretz, Susana Aznar, Shohreh Issazadeh-Navikas

**Affiliations:** 1 Neuroinflammation Unit, Biotech Research & Innovation Centre (BRIC), Faculty of Health and Medical Sciences, University of Copenhagen, Copenhagen Biocentre, Ole Maaløes Vej 5, DK-2200 Copenhagen N, Denmark; 2 Department of Medicine, University Medical Center Hamburg-Eppendorf, Hamburg, Germany; 3 Centre for Neuroscience and Stereology, University Hospital Bispebjerg-Frederiksberg, 2400 Copenhagen, Denmark

**Keywords:** Molecular biology, Psychiatric disorders

## Abstract

In the field of neurodegenerative diseases, especially sporadic Parkinson’s disease (sPD) with dementia (sPDD), the question of how the disease starts and spreads in the brain remains central. While prion-like proteins have been designated as a culprit, recent studies suggest the involvement of additional factors. We found that oxidative stress, damaged DNA binding, cytosolic DNA sensing, and Toll-Like Receptor (TLR)4/9 activation pathways are strongly associated with the sPDD transcriptome, which has dysregulated type I Interferon (IFN) signaling. In sPD patients, we confirmed deletions of mitochondrial (mt)DNA in the medial frontal gyrus, suggesting a potential role of damaged mtDNA in the disease pathophysiology. To explore its contribution to pathology, we used spontaneous models of sPDD caused by deletion of type I IFN signaling (*Ifnb*^*–/–*^/*Ifnar*^*–/–*^ mice). We found that the lack of neuronal IFNβ/IFNAR leads to oxidization, mutation, and deletion in mtDNA, which is subsequently released outside the neurons. Injecting damaged mtDNA into mouse brain induced PDD-like behavioral symptoms, including neuropsychiatric, motor, and cognitive impairments. Furthermore, it caused neurodegeneration in brain regions distant from the injection site, suggesting that damaged mtDNA triggers spread of PDD characteristics in an “infectious-like” manner. We also discovered that the mechanism through which damaged mtDNA causes pathology in healthy neurons is independent of Cyclic GMP-AMP synthase and IFNβ/IFNAR, but rather involves the dual activation of TLR9/4 pathways, resulting in increased oxidative stress and neuronal cell death, respectively. Our proteomic analysis of extracellular vesicles containing damaged mtDNA identified the TLR4 activator, Ribosomal Protein S3 as a key protein involved in recognizing and extruding damaged mtDNA. These findings might shed light on new molecular pathways through which damaged mtDNA initiates and spreads PD-like disease, potentially opening new avenues for therapeutic interventions or disease monitoring.

## Introduction

PD is the most common motor neurodegenerative disease. It is a multifactorial pathology with complex etiology mainly characterized by the loss of neurons in the Substantia Nigra (SN) [1]. Even though the exact cause of the neuronal death remains unknown, dysfunctional mitochondria is suggested to play a detrimental role. Neurons have high energy demands due to their elongated morphology [2] and their physiological functions, including membrane-gradient restoration after action potential [3] and recycling of neurotransmitters [4]. Neuronal energy is provided by mitochondria through oxidative metabolism. Genetic studies on familial PD have described monogenic forms with disease-causing mutations in several genes leading directly to mitochondrial dysfunction, including PARK2 (encoding Parkin), PARK6 (encoding Pink1), PARK8, and PARK7 (encoding DJ1) [5–10].

Due to their ancestral endosymbiotic origin, mitochondria possess their own DNA (mtDNA) which, similarly to bacterial DNA, is circular and rich in unmethylated CpG motifs. In mammals, it encodes 13 proteins of the respiratory chain, while most of the proteins involved in mitochondrial homeostasis are encoded by the nuclear genome. To balance the pool of mtDNA molecules over the number of mitochondria and cellular energy needs, mitochondrial homeostasis and mtDNA biogenesis and maintenance are tightly co-regulated [11, 12]. Upon damage, mtDNA is degraded through mitophagy [13]. Since mtDNA is essential for energy production by the respiratory chain and is therefore critical for neuronal welfare, mtDNA alterations could lead to neurodegeneration.

Genetic variations of mtDNA increase with age [14] and are reported to be associated with PD [14, 15] albeit with conflicting outcomes. These discrepancies may be attributed to the diverse pathology observed in PD and PDD and the differences in brain regions investigated [14, 16–20]. Nevertheless, strong evidence from PD animal models supports the involvement of mitochondrial disturbances including mtDNA packaging [21]. However, there is a large gap in our understanding of the molecular chain of events underlying mitochondrial dysfunctions, their impact on the nature of mtDNA and how damages to mtDNA can further trigger PD pathology and its spread leading to the clinical manifestations.

Interferon-beta (IFNβ) is a neuroprotective cytokine, which plays a central role in viral infection [22, 23], treatment of Multiple Sclerosis (MS) [24] cerebral ischemia [25, 26] and also in preventing neurodegeneration [27, 28]. In sporadic PD patients, dysregulated IFNβ signaling was identified as the top candidate pathway associated with disease and its progression to dementia, and subsequent meta-analysis of GWAS recognized sequence variants in IFNβ-IFNAR signaling pathway related genes [28]. Accordingly, mice knock-out for *Ifnb* or *Ifnar1* develop Parkinson-like disease with dementia (PDD) [27]. This is accompanied with proteinopathy, neurodegeneration including dopaminergic neuron death, and autophagy deficiency [27]. Interestingly, removal of the major accumulated protein associated with PD, α-synuclein (α-syn), still allowed proteinopathy and Parkinson-like phenotypes to develop, emphasizing a central role for IFNβ in preventing PD pathological features [29]. Using the PD *Ifnb*^−/−^ mouse model, we have previously demonstrated that neuronal IFNβ is necessary for maintaining mitochondrial homeostasis and metabolism [30]. Lack of neuronal IFNβ [30] or perturbation of its downstream signaling [28, 31] causes accumulation of damaged mitochondria with excessive oxidative stress and insufficient ATP production [30].

Our current study reveals that PDD patients with defective type I IFN signaling [28] display dysregulated transcriptomic pathways related to oxidative stress, damaged DNA binding, and cytosolic DNA sensing. Investigating another cohort of PDD patients revealed mtDNA deletions in complex I respiratory chain subunits. To understand the role of IFN in mtDNA damages and PD pathology, we used *Ifnb/Ifnar1* deleted mice. We demonstrate that neuronal IFNβ-IFNAR1 signaling deficiency leads to mtDNA damage and its extrusion both intra-cytoplasmically and extra-neuronally. Injecting damaged mtDNA into healthy mouse brain causes PDD-like pathology and motor and cognitive impairments, while healthy mtDNA does not. Damaged mtDNA also spreads the pathology to other brain regions. This occurs through TLR4 and TLR9 activation, via TLR4-activator protein, Ribosomal Protein S3 (Rps3) [32] that plays a key role in mtDNA damage recognition, extrusion, and neurotoxicity.

## Materials and methods

Detailed information is found in supplementary materials.

### Human brain samples

Human brain samples from healthy controls (NCs) (*N* = 7, 3 females and 4 males) and sPD patients (*N* = 7, 3 females, 4 males) were collected from the medial frontal gyrus, a region implicated in cognitive impairments in PD [33, 34]. The samples were obtained from the Harvard Brain Tissue Resource Center, USA, Bispebjerg Hospital Brain Bank, Copenhagen, Denmark, and Netherlands Brain Bank with appropriate ethical approvals. Diagnoses were confirmed by postmortem pathology.

For microarray analysis, publicly available data from Parkinson’s disease dementia (PDD) and without dementia (PD/PDND), and healthy controls (HC) were used as reported [28].

### Mice

*Ifnb*^*–/–*^ or *Ifnar*^*–/–*^ mice in C57BL6 background were utilized as models for PD [27, 28] and compared to their littermates/wildtype (also referred to *Ifnb*^*+/+*^/*Ifnar*^*+/+*^/WT). Experiments followed the Danish ethical license 2018-15-0201-01572. All injections and behavioral tests were performed in a randomized double-blind manner.

### mtDNA sequencing by MitoSV-seq

mtDNA sequencing was performed and analysed using the MitoSV-seq method on DNA extracted from Dopaminergic Neurons (DN) FACS-purified from 6-week-old mouse brain as described previously [35].

### mtDNA injection

mtDNA was isolated from wild-type, *Ifnb*^*−/−*^ or *Ifnar1*^*−/−*^ primary cortical neurons [27] using the DNA blood and tissue kit (Qiagen) from mitochondria fraction obtained as described earlier [30]. mtDNA was injected in 10-week-old C57BL/6 mice. By using a stereotactic instrument, 2 µL of a 10 ng/µL mtDNA solution were injected using a 10 μl Hamilton syringe on both sides in the Frontal Cortex (A/P + 1,8; M/L 1,0; D/V 2,5), the Substantia Nigra (A/P −3,0; M/L 1,3; D/V −3,9) and the Striatum (A/P 0,3; M/L 2,3; D/V −2,9) relative to the bregma. The mtDNA solution was infused at 2 μL/min. Eight mice per group were used [29]. Behavior was studied as described previously [27–29], 15 and 30 days post injection. Mice were euthanized 1-month post-injection of mtDNA and brains were collected for further assessment [27–29].

### Cell culture and treatments

Primary cortical neuron (CN) cultures were obtained from cortices of 0–1-day-old pups, dissociated with Papain (2 mg/mL) and single cell suspended using a Pasteur pipette carefully. Cells were cultured in poly-D-lysine hydrobromide coated dishes with Buffer B.

Immortalized Mouse Embryonic Fibroblasts (MEFs), NTC and *Ifnb*^*−/*−^ Neuro2A (N2A) neuroblastoma cell line were generated as described earlier [30].

*mtDNA treatment* in vitro*:* N2As or CNs (DIV7–9) were treated for 24 h with 30 ng/mL of WT or *Ifnb*^*−/−*^ mtDNA.

*Knock-down:* N2As and CNs were transfected with the respective siRNAs (siRps3 or siTLR4) using Lipofectamine RNAiMAX (Invitrogen 13778030).

*Inhibitors:* ODN-2088 (InvivoGen Catalog code: tlrl-2088) was used at a final concentration of 2.5 μM, LPS-rs (InvivoGen #tlrl-prslps) at 0.5 ng/mL and RU.521 (InvivoGen cat. Inh-ru521) at 0.5 ng/mL for 24 h according to manufacturer´s protocol (Supplementary table 1).

### Western Blotting (WB)

WB was performed as described [30] using the antibodies listed in Supplementary Table 2.

### qPCR

qPCR was performed as described [30] using primers in Supplementary Table 3.

### Immunostaining

Immunostainings and imaging were performed as described [30]. Antibodies and concentration used are listed in Supplementary Table 2.

### Electron microscopy (EM)

Sample preparation details in Supplementary. Images were acquired with a Philips CM 100 Transmission EM (Philips, Eindhoven, The Netherlands) at an accelerating voltage of 80 kV [30].

### Extracellular Vesicles (EVs) purification

EVs were purified from CN culture medium, using the ExoEasy kit (Qiagen). EVs were then pelleted by ultracentrifugation at 160,000 *g* for 2 h at 4 °C and resuspended in culture medium for EV treatment or in lysis buffer for protein analysis or in PBS for DNA purification.

From brain tissue, EVs were purified from freshly collected tissue as described [36].

### EVs Mass Spectrometry and proteomic analysis

Purified EVs were resuspended in Guanidine buffer and run at DTU Proteomics facility for LC-MS. Differential expression proteomics analysis was performed using DEP package (Bioconductor) in RStudio (version 1.4.1106 with R version 4.0.4).

### Immunoprecipitation (IP)

*mtDNA/Rps3*: Neuro2A cells were cultured until 80% confluency, medium was removed, and cells were cross-linked in 1% PFA. Cells were lysed in RIPA, quantified and immunoprecipitated with 30 µL of pre-cleared Protein G Sepharose 4 Fast Flow beads and 6 µL anti-Rps3 ON at 4 °C. After washes DNA was eluted in 1% SDS, 100 mM NaHCO3, crosslink was reversed with NaCl and DNA was purified and assessed by qPCR.

*Rps3/TLR4:* IP was performed as described [37] using anti-Rps3 antibody (4 µg/mL).

### Statistics

For all graphs, 1 dot means 1 individual patient, animal or independent biological replicate. € means *p* < 0.05, €€ *p* < 0.01, €€€ *p* < 0.001, and €€€€ *p* < 0.0001 for ANOVA, ordinary one-way ANOVA, Krustal–Wallis ANOVA if distribution did not show Gaussian distribution (Shapiro–Wilk test) or Brown-Forsythe ANOVA if distribution showed SD differences (Bartlett’s test). * means *p* < 0.05, ** *p* < 0.01, *** *p* < 0.001, and **** *p* < 0.0001 for *t*-test.

## Results

### Lack of IFNβ/IFNAR signaling causes mtDNA oxidization and mutation in a hotspot in complex I respiratory chain subunits mimicking PD brain pathology

**Fig. 1:**
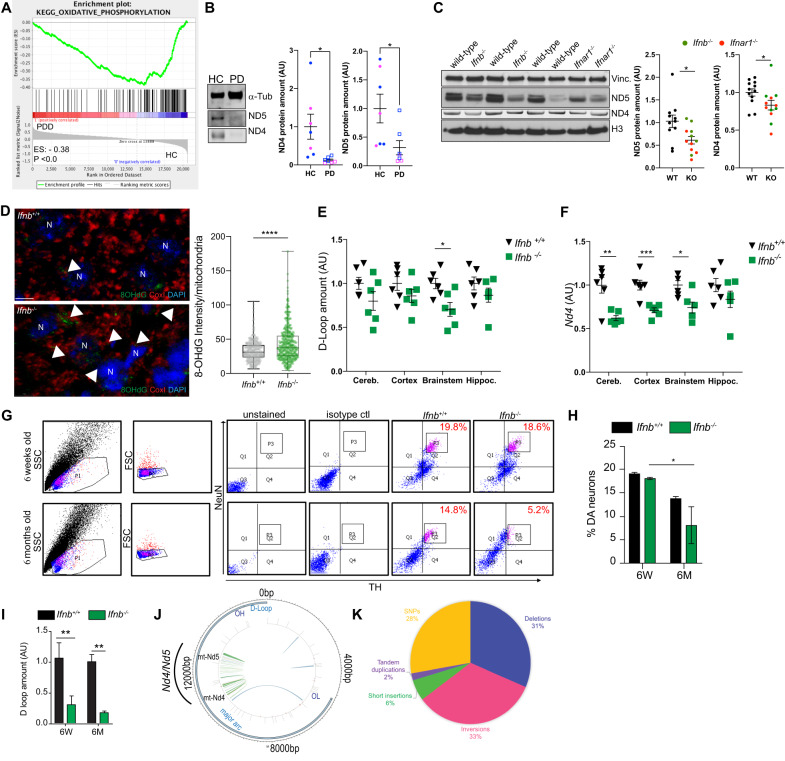
Complex I respiratory chain subunits are altered in PD patients and PD models *(Ifnb*^-/-^/*Ifnar1*^-/-^) due to mtDNA damages. **A** Enrichment plot of KEGG-OXPHOS from microarray data comparing sporadic Parkinson’s disease with dementia (sPDD/N = 13) and healthy controls (HC/N = 14). **B** Immunoblot showing loss of ND4 and ND5 proteins in the prefrontal cortex of healthy controls (HC) or Parkinson Disease patients (PD), with quantification. Pink indicates females and blue males. **C** Immunoblot showing loss of ND4 and ND5 proteins in the brainstems of 12-month-old mice, with quantifications. **D** Immunolabelling for 8-OHdG (pseudocoloured green), CoxI (mitochondria, pseudocoloured red) in mice midbrains. DAPI stains nuclei. Scale bars equal 5 microns. Quantification of 8-OHdG as integrated density of 8-OHdG in mitochondria of 3-month-old mice. Each dot represents a mitochondrion assessed (*N* = 415) from 3 biological replicates. **E** qPCR for D-loop in different brain regions in 6-week-old mice. **F** qPCR for major arc based on *Nd4* amplification in different brain regions in 6-week-old mice. For **E** and **F** each dot represents one individual animal, *N* = 6. **G** FACS cell sorting to isolate single dopaminergic (DN) neurons from midbrain of 6-week and 6-month-old mice. NeuN^+^ indicate neurons and NeuN^+^TH^+^, dopaminergic neurons. **H** Quantification of (**G**) in 6-week-old or 6-month-old mice. *N* = 3. **I** qPCR for D-loop in single isolated DN. Data are mean ± SEM, *N* = 3 mice/group. **J** Position of SVs and SNVs detected with MitoSV-seq in 6-week-old *Ifnb*^−/−^ mice. Circos plots of mouse mtDNA genome displaying Heavy and Light Origin of replication (OH and OL, respectively), major arc and D-loop (outer blue circle). mtDNA genes are displayed in the middle circle (grey lines). SVs as arches with deletions (blue), tandem duplications, inversions and insertions (orange). Thickness of the arches correspond to SVs heteroplasmy. SNVs are marked with short red lines (¬_) according to their positions in mouse mtDNA. Intensity of red lines correspond to SNVs’ heteroplasmy. **K** Pie chart showing the distribution of variation types detected in *Ifnb*^*−/−*^ mice; SNP: Single Nucleotide Polymorphism. For all graphs, * *p* < 0.05, ** *p* < 0.01, *** *p* < 0.001, and **** *p* < 0.0001 by unpaired *t*-test.

We analyzed transcriptomic datasets from sPD patients [28] to identify molecular pathways related to the disease pathology. Dysregulated oxidative phosphorylation (OXPHOS) emerged as the top-ranked pathway in sPDD, patients with reported defective type I IFN-signaling [28], compared to HC (Fig. 1A, Supplementary tables 4, 5). This OXPHOS dysregulation was also observed in sPD patients compared to HC (Supplementary table 6). OXPHOS is a crucial neuronal process for energy generation, and its imbalance can lead to excessive production of Reactive Oxygen Species (ROS), causing oxidative mtDNA mutations and damages.

In post-mortem brain samples from another cohort of sPD patients, we observed a loss of ND4 and ND5 (Fig. 1B), which are subunits of the respiratory chain responsible for OXPHOS and are encoded in mtDNA. This finding suggested a potential role of damaged mtDNA in the disease. To investigate this further, we used sPDD-murine models lacking IFNβ-IFNAR signaling, which resemble the sPDD patients [27, 28]. In *Ifnb*^−/−^ neurons, downregulation of OXPHOS was the top 7th KEGG pathway (Fig. S1A), and brain samples of both *Ifnb*^−/−^ and *Ifnar1*^−/−^ confirmed the presence of deletions in ND4 and ND5 (Fig. 1C).

*Ifnb*^−/−^ neurons exhibited high ROS levels, likely due to defective mitochondrial fission and impaired mitophagy, leading to increased oxidative DNA damages, such as 8-Hydroxyguanosine (8-OHdG) [30]. Immunolabeling of brain tissues from *Ifnb*^−/−^ mice showed positive 8-OHdG puncta localized with or near mitochondria, but not in wild-type/*Ifnb*^+/+^ brains (Fig. 1D), suggesting increased mtDNA oxidation. Quantification of mtDNA in different brain regions of *Ifnb*^−/−^ mice revealed significant loss in the D-loop and *Nd4* regions, containing a more stable DNA region vs. a more susceptible to damages respectively [38], already at 6-week-young mice (Fig. 1E, F), indicating mtDNA alterations especially in the brainstem. These findings reinforce the resemblance of these sPD-models to the neuropathology observed in patients with PD, encompassing regions beyond the basal ganglia [39, 40]. To determine the extent of these damages, we isolated DN from *Ifnb*^+/+^ and *Ifnb*^−/−^ mice using Flow Cytometry and sequenced their mtDNA by MitoSVseq [35] (Fig. 1G–K, Fig. S1B and Supplementary tables 7–9). We observed a significant loss of DN in the brains of 6-month-old mice, while the number of DN was similar in 6-week-old mice (Fig. 1G, H). Next, the loss of mtDNA was confirmed in DN already in 6-week-old mice, demonstrating that alterations of mtDNA precede DN loss in the *Ifnb*^−/−^ sPDD-model, which is observed from 3-month-old mice [27]. Sequencing revealed an accumulation of mutations in mtDNA from *Ifnb*^−/−^ DN, in a hot-spot encompassing *Nd4* and *Nd5* encoding genes (Fig. 1J). *Ifnb*^−/−^ mtDNA showed a large range of variations, presenting with 28% Single Nucleotide Polymorphisms (SNP), 31% deletions, and 33% inversions (Fig. 1K and Fig. S1B). Similar mtDNA alterations were observed in DN extracted from *Ifnar1*^−/−^ brains [35], particularly accumulating in *Nd4* and *Nd5* (Fig. S1C).

These data strongly indicates that dysregulated OXPHOS and mtDNA deletions in brains of sPD patients are co-associated with defects in type I IFN. Lack of IFNβ-IFNAR signaling leads to oxidization and indel mutations in mtDNA of DN, an early event prior to exhibition of the PDD-like manifestation in *Ifnb1*^−/−^ and *Ifnar1*^−/−^ mice. These alterations are associated with loss of the key complex I subunits from the electron transport chain, ND4 and ND5, mirroring the findings in patients with PD.

### Damaged mtDNA is extruded from cells lacking IFNβ/IFNAR

**Fig. 2:**
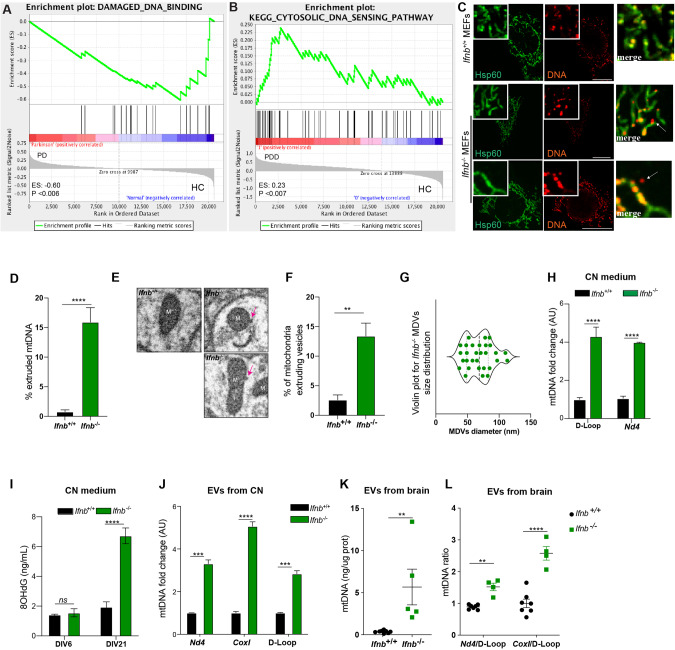
mtDNA is extruded in cells lacking IFNβ. **A** Enrichment plot of GO-Damaged DNA binding, (**B**) Enrichment plot of KEGG-Cytosolic DNA sensing from microarray data comparing sPDD (*N* = 13) and HC (*N* = 14). **C** Immunofluorescence of anti-Hsp60 (mitochondria, pseudocoloured green) and DNA (pseudocoloured red) in MEFs. Extruded mtDNA is indicated with white arrows. Scale bars equal 10 microns. **D** Quantification of extruded mtDNA as % of non-nuclear DNA+ puncta not localized in mitochondria. *N* = 3. **E** EM showing buds of vesicles being extruded from mitochondria from *Ifnb*^−/−^ thalami. Pink arrows indicate vesicles. Scale bars equal 100 nm. **F** Quantification of % of mitochondria extruding vesicles in thalami. *N* = 10 EM field/ genotype. **G** Size quantification of vesicles being extruded from mitochondria. **H** qPCR from CM of wild-type or *Ifnb*^*−/−*^ CN (DIV6) for the mtDNA genes *Nd4* and D-Loop. *N* = 3. **I** ELISA of 8-OHdG in CM of CN (DIV6, 21). *N* = 4. **J** qPCR for mtDNA *Nd4, CoxI* and D-Loop from purified EVs from CN conditioned media (DIV6). *N* = 4. **K** Quantification of mtDNA from EVs purified from brainstems of mice. *N* = 4. **L** Ratio of *Nd4/*D-Loop and of *CoxI/*D-Loop in mtDNA from EVs purified from brainstems of mice. **K**, **L** *N* = 4. For all graphs mean ± SEM. * *p* < 0.05, ** *p* < 0.01, *** *p* < 0.001, and **** *p* < 0.0001 by unpaired *t*-test.

Supporting our mtDNA-related findings, we observed the damaged DNA binding Gene Ontology (GO)-pathway as the 5th downregulated signaling in sPD patients, and cytosolic DNA sensing responses among KEGG-pathways as the 16th upregulated in sPDD (Fig. 2A, B, Supplementary tables 10–13). To explore the nature of damaged mtDNA due to the loss of IFNβ-IFNAR signaling, we stained *Ifnb*^+/+^ and *Ifnb*^−/−^ MEFs using an anti-DNA antibody alongside anti-HSP60, although not exclusive, a widely used mitochondrial marker [30, 41, 42]. Surprisingly, in *Ifnb*^−/−^ MEFs, 15% of the mtDNA puncta were found outside of mitochondria (Fig. 2C, D), indicating that *Ifnb*^−/−^ cells release mtDNA into the cytoplasm. This extruded intracytoplasmic DNA in MEFs was confirmed not to be nuclear (nu)DNA (Fig. S2A–C). 3D reconstruction of EM images of brain tissue [30] further revealed a significantly higher percentage of mitochondria from *Ifnb*^−/−^ neurons extruding vesicular structures compared to WT neurons (Fig. 2E, F), with the size resembling mitochondrial-derived vesicles (MDVs) [43] (Fig. 2G). In both *Ifnb*^−/−^ and *Ifnar1*^−/−^ CNs, mtDNA was enriched in the cytosolic fraction (Fig. S2D). Furthermore, the conditioned medium (CM) [44] from *Ifnb*^−/−^ CNs contained significantly more mtDNA, which was oxidized as evidenced by an increase in 8-OHdG levels (Fig. 2H, I). Purified Extracellular Vesicles (EVs) from *Ifnb*^−/−^ CN CM or from *Ifnb*^−/−^ brainstem also contained higher levels of mtDNA compared to wild-type (Fig. 2J, K). This mtDNA in *Ifnb*^−/−^ brain showed a heavy load of alterations (Fig. 2L), as observed earlier.

In conclusion, our results provide evidence that neurons lacking IFNβ/IFNAR1 are extruding damaged mtDNA in EVs likely originating from mitochondria.

### Damaged mtDNA is pathogenic, induces neurodegeneration and clinical deficits like PDD

**Fig. 3:**
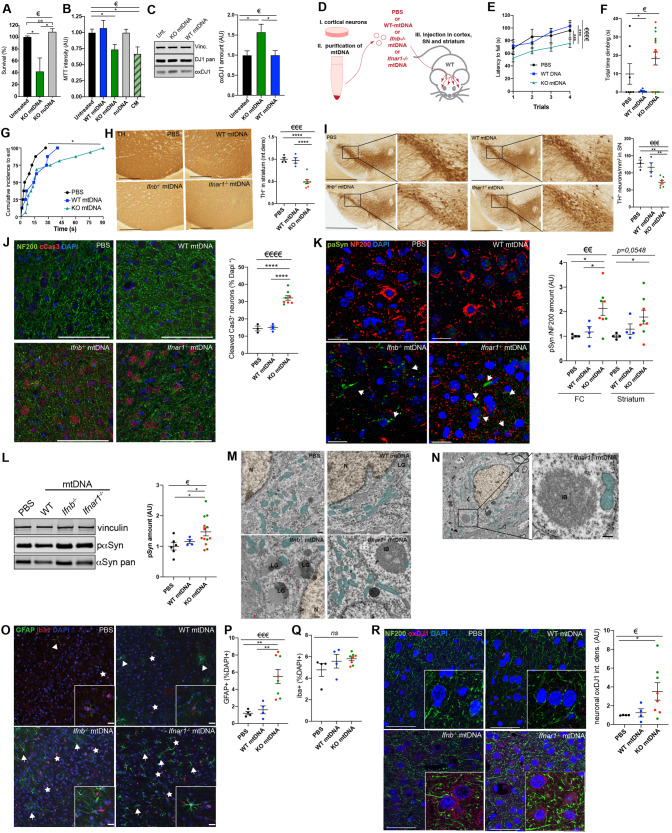
Damaged mtDNA is neurotoxic in vitro and in vivo. **A** Survival of CNs assessed by crystal violet staining upon treatment for 24 h with either KOmtDNA or nuDNA. *N* = 3. **B** CN metabolism assessed by MTT assay upon treatment for 24 h with either KO or WT mtDNA, nuDNA or CM. *N* = 4. **C** Oxidative stress assessed in wild-type CNs upon treatment for 24 h with either WT or KO mtDNA by immunoblot anti-oxDJ1 and quantification. *N* = 6. **D** Schematic of injection of mtDNA in mouse brain. **E–G** Motor and cognitive evaluation of mice injected with either PBS (black, *N* = 8), WTmtDNA (blue, *N* = 8), or KOmtDNA (*Ifnb*^−/−^ mtDNA: green, *N* = 8; *Ifnar1*^−/−^ mtDNA: red, *N* = 8). **E** Rotarod. Statistics calculated from AUC. **F** Cylinder test. **G** Cumulative Incidence to find probe during Barnes Maze. *P-val* = *0,0287* upon Mantel-Cox statistical assessment. **E**–**G** *N* = 8 **H** TH staining in striatum with quantification. Scale bars equal 1 mm. **I** TH staining in SN with quantification. Scale bars equal 500 microns. **J** Immunostaining anti-cCas3, NF200 (neurons) and DAPI (nuclei) in FC and quantification. Scale bars equal 100 microns. **K** Projection of immunostaining anti-pαSyn, NF200 (neurons) and DAPI (nuclei) in FC and quantification. White arrowheads indicate neuronal perinuclear accumulation of pαSyn. Scale bars equal 20 microns. **H**–**K** *N* = 4. **L** Immunoblotting for pαSyn and quantification. Vinculin was used as housekeeping. *N* = 4–6. **M**,**N** EM of injected mice 3 months post-injection. **M** Neuronal cell bodies overview. **N** Enlargement showing an inclusion body at high magnification. For all EM images, scale bars equal 200 nm, nuclei are underlined in orange, mitochondria in teal, LG means Lipofuscin Granules, IB means Inclusion Bodies, purple arrows indicate tangles. **O** Gliosis assessment by immunostaining anti-GFAP (astrocytes, green, white star) and anti-Iba1 (microglia, red, white arrowhead). **P** Quantification of GFAP staining in (**O**). **Q** quantification of Iba1 staining in (**O**). **R** Immunostaining for oxDJ1 in FC and quantification. Scale bars equal 50 microns. **O**–**R** *N* = 4. For all graphs, 1 dot means 1 individual animal. PBS is shown in black, WT mtDNA in blue, and KO mtDNA in green (*Ifnb*^−/−^ mtDNA) and red (*Ifnar1*^−/−^ mtDNA). € means *p* < 0.05, €€ *p* < 0.01, €€€ *p* < 0.001, and €€€€ *p* < 0.0001 ordinary one-way ANOVA, Krustal-Wallis ANOVA if distribution did not show Gaussian distribution (Shapiro–Wilk test) or Brown-Forsythe ANOVA if distribution showed SD differences (Bartlett’s test). * means *p* < 0.05, ** *p* < 0.01, *** *p* < 0.001, and **** *p* < 0.0001 by post-hoc unpaired *t*-test.

We examined the impact of extruded altered mtDNA on neuronal homeostasis in vitro and in vivo. Treating healthy wild-type primary neurons with damaged mtDNA from *Ifnb*^−/−^ neurons (KOmtDNA) induced neuronal cell-death, while nuDNA at the same concentration did not have the same effect (Fig. 3A). Similar results were observed in N2A cells (Fig. S3A). Remarkably, only KO damaged mtDNA, not an equivalent concentration of WTmtDNA, or even higher doses (up to 300 ng/mL) elicited the same neuronal death response (Fig. S3B, C). This contradicts previous reports suggesting that undamaged mtDNA can cause cellular toxicity. However, these effects were observed at doses much higher (up to 100 times) [45, 46] than those used in our study. Furthermore, KOmtDNA induced neuronal loss in both healthy and *Ifnb*^−/−^ neurons (Fig. S3C–E). Thus, damaged mtDNA-induced neuronal death does not require endogenous IFNβ-IFNAR signaling for its detrimental mode of action. Interestingly, even WTmtDNA increased neuronal death and astrogliosis in vulnerable *Ifnb*^−/−^ neurons (Fig. S3D, E). These results were confirmed by the assessment of primary neurons’ viability, where only damaged *Ifnb*^−/−^ mtDNA treatment decreased MTT levels. Treating primary neurons with *Ifnb*^−/−^ CM, containing EVs with damaged mtDNA, had a similar impact (Fig. 3B). In addition, treatment with damaged KOmtDNA increased oxidative stress by elevating oxidized deglycase-1, (ox)DJ1 [28, 30], in healthy WT neurons, while WTmtDNA did not impact DJ1 oxidation (Fig.3C).

Moving to in vivo experiments, we injected mtDNA extracted from WT, *Ifnb*^−/−^, or *Ifnar1*^−/−^ neurons into the brains of WT mice (Fig. 3D) and assessed their motor, neuropsychiatric, and cognitive abilities one month later. While there were no differences in weight observed, mice injected with damaged KOmtDNA showed impaired performance in motor tasks compared to those injected with mtDNA from WT neurons (Fig. 3E, Fig. S3F–H). They also exhibited increased climbing time and climbing events, potentially related to anxiety (Fig. 3F, Fig. S3I). In a Barnes Maze test, mice injected with damaged KOmtDNA required more time to find the exit-box compared to control mice injected with PBS (Fig. 3G) indicative of cognitive impairments. Staining of brain regions revealed significant neuronal loss, including DN (TH^+^) in striatum/STR and SN, increased cCas3^+^NF200^+^ and phosphorylated (p)α-syn^+^ proteinopathy associated with damaged KOmtDNA injection, particularly in the frontal cortex (FC) (Fig. 3H–K), confirmed by immunoblotting of brainstem lysates (SN, STR and thalamus) (Fig. 3L).

Three months post injection, EM revealed inclusion bodies (IB) and an accumulation of lipofuscin granules (LG) associated to neuromelanin, both pathogenic features of ND, only in the STR of damaged KOmtDNA injected animals (Fig. 3M, N). Furthermore, the mice injected with KOmtDNA displayed astrogliosis, a typical feature of PD [44], in the cortices (Fig. 3O, P). No significant differences in microgliosis (Iba1^+^) were observed between WTmtDNA and KOmtDNA injected groups (Fig. 3O, Q). In addition, injected damaged KOmtDNA resulted in increased oxDJ1 levels in the brain (Fig. 3R, Fig. S3J).

In conclusion, these results demonstrate that damaged and extruded *Ifnb*^−/−^/*Ifnar1*^−/−^ mtDNA is neurotoxic not only in vitro but also in vivo, recreating PDD-like pathology and leading to anxiety, motor and cognitive dysfunctions in mice. This is associated with neuronal loss including DNs, accumulation of pα-syn proteinopathy, oxidative stress, and astrogliosis in the injected brain regions.

### Altered *Ifnb*^−/−^/*Ifnar1*^−/−^ mtDNA propagates neurodegeneration in distant brain regions

**Fig. 4:**
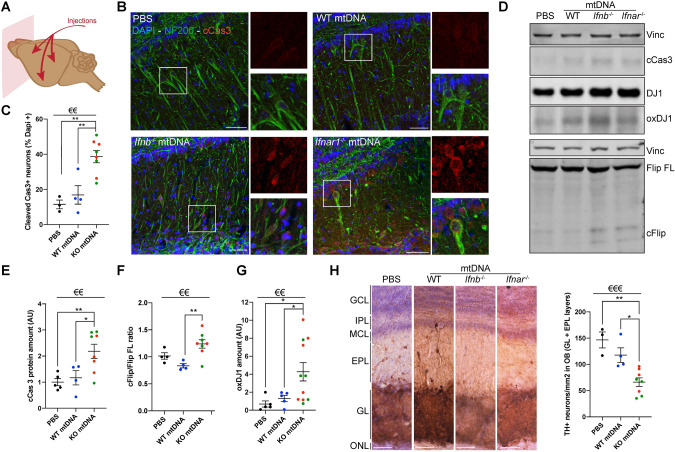
Damaged mtDNA spreads neurodegeneration in other part of the brain. **A** Schematic showing the strategy of investigation in OB. Tissue was evaluated one-month post-injection. **B** Immunofluorescence for cCas3 (red) and NF200 (green, neurons) in OB from injected mice. Scale bars equal 50 microns. **C** Quantification of (**B**). **D–G** Immunoblots and quantifications of proteins extracted from OB from mice injected with PBS, WTmtDNA, *Ifnb*^−/−^mtDNA or *Ifnar1*^*−/*−^mtDNA for cell death markers. **E** cCas3 and (**F**) Flip, and oxidative stress marker (**G**). oxDJ1. **H** Immunolabelling of TH^+^ neurons (reddish brown) with hematoxylin (blue) counterstain in OB from injected mice and quantification. For all graphs, 1 dot means 1 individual animal. PBS is shown in black, WTmtDNA in blue, and KOmtDNA in green (*Ifnb*^−/−^mtDNA) and red (*Ifnar1* ^−/−^mtDNA), *N* = 4–8/group. €€ means *p* < 0.01 and €€€ *p* < 0.001 by ordinary one-way ANOVA, Krustal-Wallis ANOVA if distribution did not show Gaussian distribution (Shapiro–Wilk test) or Brown-Forsythe ANOVA if distribution showed SD differences (Bartlett’s test). * means *p* < 0.05 and ** *p* < 0.01, by post-hoc unpaired *t*-test.

We investigated whether damaged KOmtDNA could propagate pathology to distant brain regions, considering that over 80% of PD patients develop dementia [47], and extended regional pathology in the brain [33]. The olfactory bulbs (OB) were studied as they are suggested to be primarily affected during PD development [48]. Mice injected with damaged KOmtDNA showed significantly elevated cCas3^+^ staining in the OB, indicating active neuronal death (Fig. 4A–C). Immunoblotting confirmed increased markers of apoptosis, cCas3 and cFLIP, in the OB (Fig. 4D–F). Oxidative stress, indicated by elevated oxDJ1 levels, was also observed in the OB (Fig. 4D, G). Moreover, specific DN loss in the Glomerular Layer (GL) and External Plexiform Layer (EPL) of the OB was significant in mice injected with damaged KOmtDNA (Fig. 4H). Similar spreading of neuronal death was observed in the cerebellum (Fig. S4). Notably, the spread could likely be due to axonal dopaminergic projections from the SN to the EPL, granule cell layer, and mitral cell layer of the OB, ablation of which impairs olfactory perception [49], and/or the injected solutions could potentially enter the cerebrospinal fluid and reach other brain regions, contributing to the spread of pathology.

These results demonstrate that damaged *Ifnb*^−/−^/*Ifnar1*^−/−^ mtDNA not only induces clinical and pathological manifestations of PDD in mice but also propagates the pathology to distant neurons in other brain regions.

### Upregulated TLR9 in PDD patients and damaged mtDNA-injected brains are related to mitochondrial oxidative stress

**Fig. 5:**
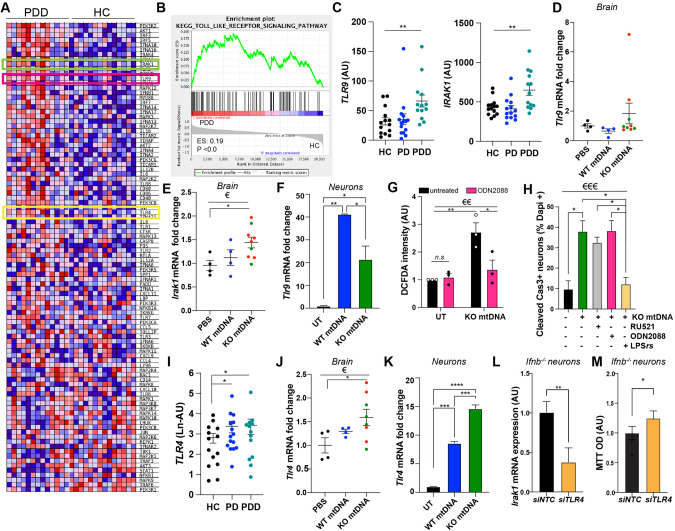
TLR9 signalling is upregulated in PDD patient and triggers oxidative stress in neurons, while TLR4 triggers neuronal death. **A** Heat map from GSEA of the TLR signalling pathway and comparing microarray data from sPDD (*N* = 13) and HC (*N* = 14). TLR9 is circled in green and TLR4 in yellow. **B** Enrichment plot of the TLR signalling pathway. **C** Expression of *TLR9* and *IRAK1* in HC and PDD patients extracted from (**A**). **D**, **E** mRNA levels of (**D**). *Tlr9* and (**E**). *Irak1* in mice injected with either PBS (*N* = 4), WTmtDNA (*N* = 4) or KO mtDNA (*N* = 9), (**F**) mRNA levels of *Tlr9* in primary CNs untreated or treated with either WT mtDNA or KO (*Ifnb*^−/−^) mtDNA. One representative of 3 independent experiments. **G** Normalized DCFDA integrated density quantification wild-type neurons treated with KOmtDNA with or without the presence of TLR9 inhibitor ODN2088. **H** Quantification of cCas3 staining in WT neurons treated with KOmtDNA with or without the presence of cGAS inhibitor RU521, TLR4 inhibitor LPS *rs* or ODN2088. Immunofluorescence staining in S5D. **G**, **H** *N* = 3. **I** Expression of TLR4 in HC and PDD patients extracted from (**A**). **J** mRNA levels of *Tlr4* in mice injected with either PBS (*N* = 4), WTmtDNA (*N* = 4) or KOmtDNA (*N* = 9). **K** mRNA levels of *Tlr4* in primary CNs untreated or treated with either WTmtDNA or KO (*Ifnb*^−/−^) mtDNA. One representative of 3 independent experiments. **L** *Irak1* expression in *Ifnb*^−/−^ CNs NTC or knock-down for *Tlr4*. **M** Viability of *Ifnb*^−/−^ CNs NTC or knock-down for *Tlr4* assessed by MTT staining. For all graphs, 1 dot means 1 individual animal. For (**D**, **E** and **J**) PBS injected mice are shown in black, WTmtDNA in blue, and KOmtDNA in green (*Ifnb*^−/−^mtDNA) and red (*Ifnar1* ^−/−^mtDNA), *N* = 4/group. €€ means *p* < 0.01 and €€€ *p* < 0.001 by ordinary one-way ANOVA, Krustal–Wallis ANOVA if distribution did not show Gaussian distribution (Shapiro–Wilk test) or Brown-Forsythe ANOVA if distribution showed SD differences (Bartlett’s test). **G** Was analyzed by 2way-ANOVA. * means *p* < 0.05 and ** *p* < 0.01, *** *p* < 0.001, and **** *p* < 0.0001 by post-hoc unpaired *t*-test.

To understand the pathological impact of damaged mtDNA due to dysfunctional IFNβ-IFNAR signaling, we reanalyzed transcriptomic data from sPDD patients and HC [28] for potential molecular mechanisms. We identified the TLR-signaling pathway among the top 20 significant KEGG-pathways associated with sPDD (11th, Supplementary tables 12, 14, Fig. 5A, B). *TLR9* and its downstream signaling protein *IRAK1* were significantly upregulated in sPDD patients compared to HC (Fig. 5C). TLR9 recognizes unmethylated CpG motifs in bacterial DNA and mtDNA [50, 51], we investigated whether TLR9 was activated in response to extruded damaged KOmtDNA. We found modestly increased *Tlr9* expression (Fig. 5D), and *Irak1* mRNA expression significantly upregulated in the brain of mice injected with KOmtDNA compared to controls (Fig. 5E). In primary neurons, *Tlr9* and *Irak1* mRNA were also upregulated upon mtDNA treatment (Fig. 5F, Fig. S5A), indicating a general plausible homeostatic role in neuronal response to free-mtDNA.

To explore the involvement of TLR9 in neuronal dysfunction caused by damaged KOmtDNA, we inhibited TLR9 using a specific antagonist, ODN2088 in cortical neurons. We observed an efficient blockade of the signaling, leading to downregulation of downstream proteins phosphorylated TANK-binding kinase 1 (pTBK1) and phosphorylated Interferon Regulatory Factor 7 (pIRF7), but it did not prevent cell-death in primary neurons (Fig. S5B, D).

In neurons lacking IFNβ signaling and impaired mitochondrial activity, TLR9 inhibition positively affected mitochondrial health, reducing the production of various ROS species as assessed by DCFDA and MitoSox stainings (Fig. 5G, Fig. S5E, F). In addition, using another selective inhibitor of superoxide production from mitochondrial respiratory complex site III, S3qel, further reduced DCFDA intensity when combined with the TLR9 inhibitor (Fig. S5E). In support, TLR9 inhibition also decreased *Sod2* mRNA and oxDJ1, while increasing the mitochondrial membrane potential in *Ifnb*^*−/*−^ neurons (Fig. S5G–I). Although TLR9 inhibition showed a positive impact on oxidative stress markers, it did not prevent cell-death or rescue apoptotic cCas3^+^ neurons (Fig. 5H, Fig. S5C, D).

Our data support a role for TLR9 in inducing oxidative stress upon recognizing damaged mtDNA, but not in mtDNA-induced neuronal death.

### TLR4 is upregulated in response to damaged mtDNA and triggers neuronal death

We observed significant upregulation of TLR4 in the transcriptomes of both PD and PDD patient groups (Fig. 5A and I), as well as in the brains of mice injected with damaged mtDNA (Fig. 5J), and primary neurons treated with KOmtDNA (Fig. 5K). To investigate the functional relevance of TLR4 response to damaged mtDNA, we inhibited TLR4 using the antagonist Lipopolysaccharide [52] from the photosynthetic bacterium *Rhodobacter sphaeroides* (LPSrs) (Fig. 5H and S5D–H) and via siRNA knockdown (Fig. 5L, M and S5J, K). The inhibition of TLR4 efficiently prevented neuronal death when wildtype neurons were treated with damaged KOmtDNA (Fig. 5H, Fig. S5D). Notably, inhibition of cytosolic DNA sensor, Cyclic GMP-AMP synthase (cGAS) by using its inhibitor RU521 did not prevent neuronal death mediated by damaged KOmtDNA (Fig. 5H, Fig. S5D), consistent with no significant changes in mRNA or protein levels of cGAS, or impact in wild-type CN treated with damaged KOmtDNA (Fig. S5L, M). These data indicate that TLR4, and not TLR9 or cGAS, is the main driver of damaged mtDNA-induced neuronal death.

To establish the role of TLR4 in oxidative damages in *Ifnb*^*–/−*^ neurons, we successfully knocked down *Tlr4* (Fig. S5J), which interestingly also resulted in decreased *Irak1* expression, and increased neuronal viability compared to the control (Fig. 5L, M), while it did not impact mitochondrial membrane potential (Fig. S5K). These findings suggest that although TLR4 might impact some common downstream signaling as TLR9, it also exerts distinct functions.

In summary, these results demonstrate that damaged mtDNA from cells lacking endogenous IFNβ-IFNAR signaling induces neuronal cell-death through activation of TLR4, suggesting a new function for TLR4 in response to damaged mtDNA.

### TLR4 activator Rps3 recognizes damaged mtDNA, an essential step for its extrusion

**Fig. 6:**
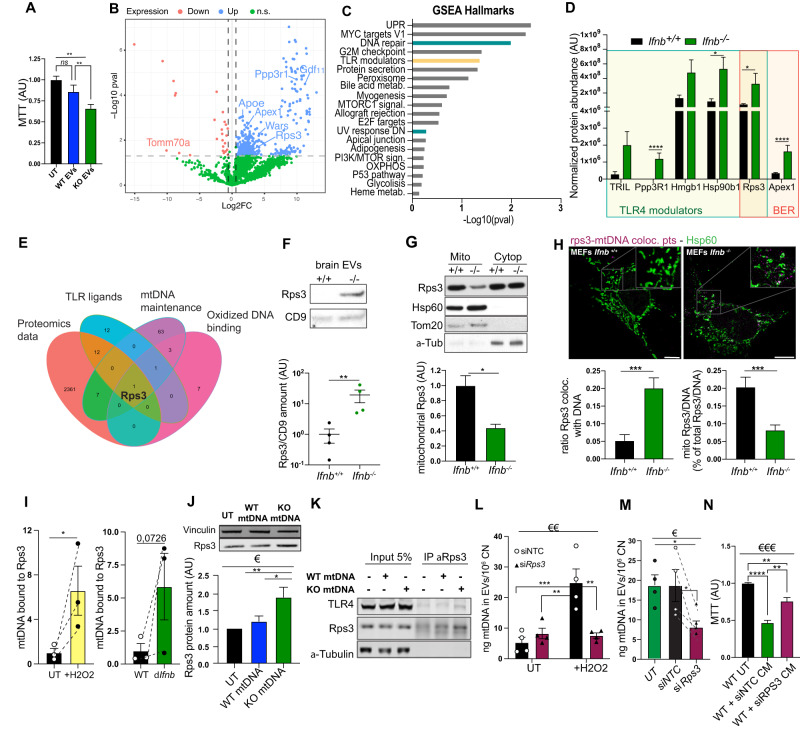
TLR4 activator Rps3 recognizes damaged mtDNA and is essential for its extrusion in EVs. **A** Metabolic activity of CN upon treatment with EVs purified for either wild type or *Ifnb*^−/−^ CN CM. *N* = 4. **B** Volcano plot of proteins differentially expressed in EVs purified from wild-type and *Ifnb*^−/−^ CN. DEP analysis of *N* = 3. **C** Top GSEA pathways dysregulated in *Ifnb*^−/−^ EVs. **D** Normalized abundances of some TLR4 modulators and BER proteins found in *Ifnb*^−/−^ EVs. **E** Venn Diagram of the common proteins between our dataset, TLR4 ligands, mitochondrial DNA maintenance and oxidized DNA binding pathways. **F** Immunoblot for Rps3 and EV marker CD9 in EVs purified from wild-type and *Ifnb*^−/−^ mouse brains. **G** Cellular fractionation of CNs followed by immunoblot for Rps3. Hsp60 and Tom20 indicate the mitochondrial fraction and α-tubulin the cytoplasmic fraction. **H** Rps3/DNA colocalization points extracted from immunofluorescence for DNA, Rps3 and Hsp60 (mitochondria) with quantifications in *Ifnb*^+/+^ and *Ifnb*^−/−^ MEFs. *N* = 3. **I** Amount of mtDNA CoIP with Rps3 in N2A cells with or without oxidative stress induced by H_2_O_2_ or by lack of *Ifnb*. *N* = 3. **J** Immunoblot showing the amount of Rps3 in wild-type CNs treated with either WT or KO mtDNA. *N* = 3. **K** Immunoblotting showing CoIP of TLR4 with Rps3 in N2A cells untreated or treated with WTmtDNA or KOmtDNA. **L** Quantified mtDNA in EVs purified from WT CN ± H_2_O_2_ treatment with or without Rps3 knock-down. **M** Amount of mtDNA quantified in EVs purified from *Ifnb*^−/−^ CN with or without Rps3 knock-down. *N* = 4. **M** Metabolic activity assessed by MTT assay in wild-type cell treated with CM from *δIfnb* N2A with or without knock-down for Rps3, *N* = 3. For all graphs, 1 dot means 1 individual animal. €€ means *p* < 0.01 and €€€ *p* < 0.001by ordinary one-way ANOVA, Krustal–Wallis ANOVA if distribution did not show Gaussian distribution (Shapiro–Wilk test) or Brown-Forsythe ANOVA if distribution showed SD differences (Bartlett’s test). * means *p* < 0.05 and ** *p* < 0.01, by post-hoc unpaired *t*-test.

We utilized a proteomic approach to investigate additional molecular factors that may contribute to the pathogenicity of damaged KOmtDNA in relation to TLR4. As we had previously demonstrated, damaged mtDNA is packed into EVs released by neurons lacking IFNβ (Fig. 2J–L). These EVs, like pure damaged mtDNA, reduced cell survival in healthy neurons (Fig. 6A). To gain insights into the molecular mechanisms underlying the packaging of damaged mtDNA into EVs and its neurotoxic effects, we purified EVs from both wild-type and *Ifnb*^−/−^ primary CN CM and subjected them to Liquid Chromatography Mass Spectrometry (LC-MS) analysis (Fig. 6B–E, Fig. S6A–G). By employing an unbiased Label-Free Quantification (LFQ) approach to identify protein changes between *Ifnb*^*+/+*^ and *Ifnb*^−/−^ EVs, we detected a total of 2381 proteins, with 2146 proteins showing High Confidence FDR, indicating a high-quality readout (Fig. S6A). Although the protein abundance was quite similar in all 3 replicates per group, EVs purified from *Ifnb*^−/−^ neurons revealed a higher number of identified proteins compared to *Ifnb*^*+/+*^ neurons (Fig. 6B, Fig. S6D), suggesting that damaged mtDNA is released within a more complex EV pool. Principal Component Analysis (PCA) of the entire dataset and clustering of samples based on genotype in Heatmap (Fig. S6C, D) revealed a specific protein signature in the *Ifnb*^−/−^ EVs. As expected, we identified EV markers in both *Ifnb*^*+/+*^ and *Ifnb*^−/−^ EVs (Fig. S6E), including L1CAM, a protein known to be enriched in neuronal EVs [53].

Importantly, we found around 300 mitochondria-related proteins in EVs, some showing clear differences between *Ifnb*^+/+^ and *Ifnb*^−/−^ populations. For example, Tomm70a, a key mitophagy protein [54], was significantly decreased in *Ifnb*^−/−^ EVs, while HspA9, a marker of MDVs destined for lysosomal degradation [55], was increased in *Ifnb*^−/−^ EVs (Fig. 6B and S6F).

Notably, DNA repair and TLR modulator pathways were among the top 5 GO terms after GSEA analysis (Fig. 6C; Fig. S6G). One of the proteins highly enriched in *Ifnb*^−/−^ EVs was Rps3 (Fig. 6B and D). Interestingly, Rps3 was the only protein we identified belonging to TLR4 modulator [32] and Base Excision Repair (BER) pathways, which binds to ApexI [56], also upregulated in the KO EVs. Furthermore, we identified Rps3 as the only protein in our data set shared among TLR-ligands, mtDNA maintenance, and oxidized DNA binding proteins pathways (Fig. 6C–E, S6D, G and Supplementary table 11). Rps3 is about 20 times more abundant in EVs purified from *Ifnb*^−/−^ brain tissues as seen by Western Blotting (Fig. 6F).

In KO neurons, we found Rps3 mainly in the cytoplasmic fraction and not in the mitochondrial fraction, while it is found in both fractions in wild-type neurons (Fig. 6G). As Rps3 specifically recognizes 8-OHdG, oxidized alterations on DNA [57], we hypothesized that Rps3 could bind oxidized and damaged mtDNA and be extruded within EVs. Indeed, in a staining of wild-type and *Ifnb*^−/−^ MEFs we found that Rps3 was outside the mitochondria and colocalized to cytoplasmic mtDNA in *Ifnb*^−/−^ cells compared to WT (Fig. 6H and Fig. S6H).

To establish if Rps3 binds to oxidized mtDNA, we immunoprecipitated Rps3 from N2A cells followed by qPCR for mtDNA genes. We established that Rps3 directly binds damaged mtDNA in a higher proportion in δ*Ifnb* cells, but also in wild-type cells treated with a H_2_O_2_ pulse as a positive control to induce oxidative damages in mtDNA (Fig. 6I and Fig. S6I). Moreover, we observed a significant increase of Rps3 protein levels in WT CNs treated with damaged KOmtDNA, but not with WTmtDNA (Fig. 6J). To check whether Rps3 could be recognized by and activate TLR4 in neurons, we coimmunoprecipitated TLR4 using an anti-Rps3 antibody (Fig. 6K) showing that these proteins indeed bind. Moreover, knock-down of Rps3 with siRNA (Fig. S6J, K) decreased the amount of damaged mtDNA extruded in EVs purified from either δ*Ifnb* N2A media (Fig. S6L), wild-type neurons treated with H_2_O_2_ (Fig. 6L), or EVs from *Ifnb*^−/−^ CN media (Fig. 6M). Accordingly, knockdown of Rps3 reduced toxicity induced by CM from δ*Ifnb* N2A cells in WT N2As, as shown by decreased MTT (Fig. 6N).

In conclusion, we identified TLR4 activator, Rps3 as an important cofactor which recognizes and binds the damaged mtDNA extruded outside mitochondria. Damaged mtDNA-Rsp3 complexes are then extruded extra-neuronally, at least partly in EVs which in turn participate in the neurodegenerative pathology and propagation of PDD-like disease via activation of TLR pathways (Fig. S7).

## Discussion

We have previously demonstrated that disruption of the IFNβ-IFNAR1 signalling pathway is associated and can cause development of PD with dementia [27, 28, 30]. In the current study, we identified mtDNA deletions in a hotspot in complex I respiratory chain subunits associated with dysregulated oxidative stress, cytosolic DNA damages and sensing pathways in cohorts of sporadic PD and PDD, in the same cohorts in which we previously reported type I IFN disruption as a top dysregulated signaling pathway [28]. These mtDNA alterations may result from the impairment of mitochondrial fission, autophagy, and mitophagy, as observed when the IFNβ-IFNAR1 signaling pathway is disrupted [27, 28, 30]. Consistently, previous studies have demonstrated that mtDNA damages are managed through the process of mitophagy [58, 59], or alternatively released via cell-free mtDNA or enclosed within EVs [60].

To investigate the relevance of mtDNA damages in PD pathology, we utilized spontaneous mice PDD models where IFNβ or IFNAR genes are deleted [27, 28, 30]. We observed that lack of endogenous neuronal IFNβ-IFNAR signaling causes altered mtDNA, harboring sequence variations and oxidative damages. Damaged mtDNA is in turn extruded from IFNβ/IFNAR knock-out neurons partly through EVs causing and spreading neurotoxicity. Moreover, we established that, although the damages in mtDNA and their subsequent release from neurons occurs only when the endogenous IFNβ-IFNAR signaling is defective, their “infectious-like” spread of the pathology is IFNβ/IFNAR-independent. Furthermore, we identified that the damaged mtDNA extrusion is dependent on its recognition by Rps3, a protein reported to be involved in oxidative DNA repair [61] and in TLR4 activation [32], but with no previously known role in the extrusion of damaged mtDNA and neurodegeneration. Importantly, we demonstrated that the damaged mtDNA extruded from neurons is neurotoxic, notably through the coactivation of TLR9 and TLR4 which direct different molecular events. Our results established that while TLR9-dependent sensing is involved in neuronal mitochondrial oxidative stress, TLR4 activation contributes to neuronal cell-death. Of note, we did not observe a role for cGAS-STING pathway, one of the earlier suggested main players [62–64], in our reported neuronal pathologies.

Although an increased presence of mtDNA mutations in PD patients is reported [14, 19, 65], the functions of extruded and circulating mtDNA and their relationship to physiological and pathological conditions in the context of PD remain a subject of intense debate, with conflicting findings reported in various models [65–68]. Remarkably, the specific characteristics of mtDNA that are released and subsequently extruded extracellularly, as well as their in vivo implications for the propagation of PDD pathology, have not been thoroughly investigated. Macrophages are reported to fragment and release oxidized mtDNA via VDAC pores [69] and not via MDVs, thus distinct from the extrusion mechanism observed here in post-mitotic neurons.

Significantly, we demonstrated that damaged mtDNA, resulting from impaired neuronal IFNβ-IFNAR signaling, does not only induce PDD-like pathology upon injection into healthy animals, including causing motor and cognitive deficits, pα-synuclein accumulation, and neuronal loss, it also triggers the spread of the pathology to other brain regions in an “infectious-like manner”. The precise mechanisms of this spread are still to be elucidated, but the impact of damaged mtDNA on TLR9-mediated mitochondrial membrane potential reduction and oxidative stress increase suggests that damaged mtDNA could spread mitochondrial dysfunction to neighbouring healthy neurons. This is supported by the observed increase in neuronal oxDJ1, not only at the injection site but also in distant brain regions. Interestingly, previous studies have demonstrated that oxidative stress promotes prion-like protein aggregation [70].

Our findings indicate that the neuronal pathology induced by damaged mtDNA involves the coactivation of TLR9 and TLR4. We observed increased expression of both receptors in the transcriptome of PDD patients, which was further verified in another cohort of PDD patients, mouse brains injected with damaged KOmtDNA, and primary neurons treated with damaged KOmtDNA. Surprisingly, *Tlr4* showed higher upregulation upon treatment with damaged mtDNA compared to WTmtDNA. While TLR9 has been previously reported to recognize unmethylated CpG motifs from mtDNA [50, 51], the association of TLR4 with mtDNA sensing is elusive. Previous studies had suggested the involvement of TLR4 in PD pathology [71, 72], but primarily linked to microglia activation by α-synuclein [73, 74]. However, its association with mtDNA pathology and its role in neuronal death associated with PDD had not been demonstrated. In our study, we identified Rps3 as a key mediator of TLR4 activation in response to damaged mtDNA in neurons. Rps3 specifically recognizes oxidative damages on mtDNA and directly binds to TLR4. It plays a crucial role in nuDNA and mtDNA maintenance during oxidative stress in mammalian cells, including neurons [56, 57, 75–77]. We show that Rps3 is essential for the recognition of damaged mtDNA in neurons and its extrusion from mitochondria in EVs. This alternative mechanism is particularly important in neurons with defective mitophagy, such as *Ifnb*^−/−^/*Ifnar1*^−/−^ neurons. This would restrict cytosolic mtDNA leakage and subsequent cGAS activation, thereby preventing an inflammatory response [58]. However, if damaged mtDNA is released extracellularly, in cell-free form or enclosed within EVs, it may cause an infectious-like pathology upon uptake by healthy neurons.

Our data reveal a distinct role of damaged mtDNA in neurotoxicity and the spread of an infectious PDD-like pathology. This process involves the participation of EVs and the key players Rps3-TLR4 and TLR9, operating independently of cGAS and IFNβ-IFNAR signaling. The understanding of damaged mtDNA/Rps3-mediated neurodegeneration holds promises in elucidating the mechanisms underlying the propagation of Parkinson’s pathology throughout the brain and its progression to dementia. These insights may pave the way for innovative treatment strategies and monitoring approaches for Parkinson’s disease.

### Supplementary information

Supplemental Material

## Data Availability

The mass spectrometry proteomics data have been deposited to the ProteomeXchange Consortium via the PRIDE partner repository with the dataset identifier PXD038362.
