# Supplementary material for: Mitochondrial DNA damage triggers spread of Parkinson’s disease-like pathology
Source: Mol Psychiatry. 2023 Oct 2;28(11):4902–14. doi: 10.1038/s41380-023-02251-4 (PMC10914608; doi:10.1038/s41380-023-02251-4)
Supplement: Supplementary file 1 — Supplemental Material [file 41380_2023_2251_MOESM1_ESM.pdf]

Mitochondrial DNA damage triggers spread of Parkinson's disease-like pathology

Emilie Tresse[1], Joana Marturia-Navarro[1], Wei Qi Guinevere Sew[1], Marina Cisquella-Serra[1], Elham Jaberi[1], Lluis Riera-Ponsati[1], Natasha Fauerby[1], Erling Hu[1], Oliver Kretz[2], Susana Aznar[3] and Shohreh Issazadeh-Navikas[1*]

[1]Neuroinflammation Unit, Biotech Research & Innovation Centre (BRIC), Faculty of Health and Medical Sciences, University of Copenhagen, Copenhagen Biocentre, Ole Maaløes Vej 5, DK-2200 Copenhagen N, Denmark
[2]Department of Medicine, University Medical Center Hamburg-Eppendorf, Hamburg, Germany
[3]Centre for Neuroscience and Stereology, University Hospital Bispebjerg-Frederiksberg, 2400 Copenhagen, Denmark.

[*]**Corresponding author:** Shohreh Issazadeh-Navikas, Professor, Neuroinflammation Unit, Biotech Research and Innovation Centre (BRIC), University of Copenhagen, Ole Maaløes Vej 5, DK-2200 Copenhagen N, Denmark
E-mail: shohreh.issazadeh@bric.ku.dk
Tel: +45-353 25649

Running Title: ***Mitochondrial DNA damage propagates PD***

Content:

The supplementary information file contains all supplementary figures 1-7, supplementary tables 1-14, supplementary materials and methods, and a list of proteins used for generating Venn diagram.

Abstract

In the field of neurodegenerative diseases, especially sporadic Parkinson's disease (sPD) with dementia (sPDD), the question of how the disease starts and spreads in the brain remains central. While prion-like proteins have been designated as a culprit, recent studies suggest the involvement of additional factors. We found that oxidative stress, damaged DNA binding, cytosolic DNA sensing, and Toll-Like Receptor (TLR)4/9 activation pathways are strongly associated with the sPDD transcriptome, which has dysregulated type I Interferon (IFN) signaling. In sPD patients, we confirmed deletions of mitochondrial (mt)DNA in the medial frontal gyrus, suggesting a potential role of damaged mtDNA in the disease pathophysiology.

To explore its contribution to pathology, we used spontaneous models of sPDD caused by deletion of type I IFN signaling (*Ifnb*$^{-/-}$/*Ifnar*$^{-/-}$ mice). We found that the lack of neuronal IFNβ/IFNAR leads to oxidization, mutation, and deletion in mtDNA, which is subsequently released outside the neurons. Injecting damaged mtDNA into mouse brain induced PDD-like behavioral symptoms, including neuropsychiatric, motor, and cognitive impairments. Furthermore, it caused neurodegeneration in brain regions distant from the injection site, suggesting that damaged mtDNA triggers spread of PDD characteristics in an "infectious-like" manner. We also discovered that the mechanism through which damaged mtDNA causes pathology in healthy neurons is independent of Cyclic GMP-AMP synthase and IFNβ/IFNAR, but rather involves the dual activation of TLR9/4 pathways, resulting in increased oxidative stress and neuronal cell death, respectively. Our proteomic analysis of extracellular vesicles containing damaged mtDNA identified the TLR4 activator, Ribosomal Protein S3 as a key protein involved in recognizing and extruding damaged mtDNA. These findings might shed light on new molecular pathways through which damaged mtDNA initiates and spreads PD-like disease, potentially opening new avenues for therapeutic interventions or disease monitoring.

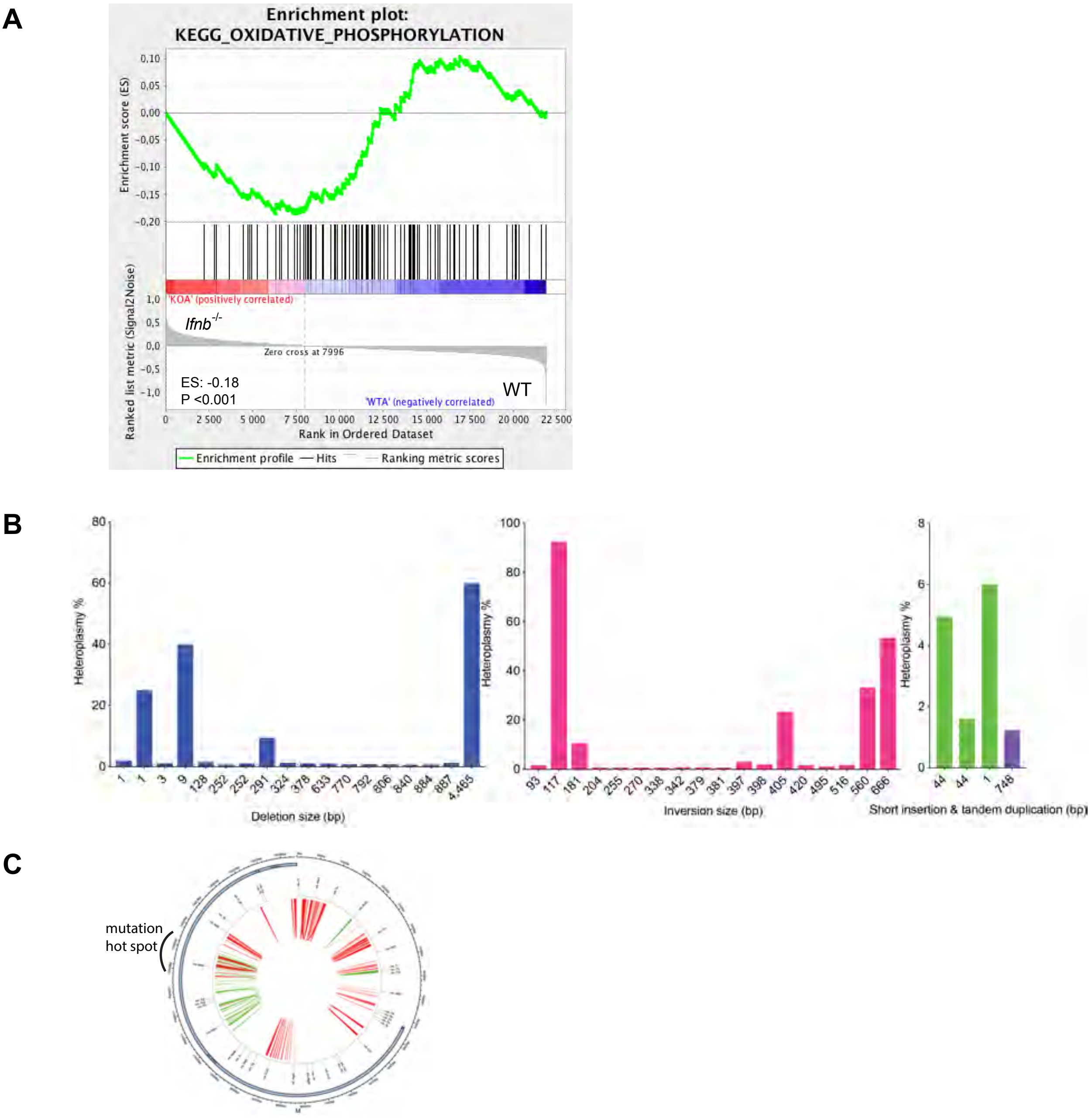

Supplementary Figure 1. The Nd4/Nd5 mutation hotspot is common to *Ifnb*$^{-/-}$ and *Ifnar1*$^{-/-}$ dopaminergic neurons. A. Oxidative phosphorylation KEGG-pathway in *Ifnb*$^{-/-}$ vs. WT neurons from microarray analysis. **B.** Distribution of heteroplasmy for deletions, inversions, short insertions, and tandem duplications according to their size (bp) (from left to right). 4,465 bp deletions is the PC in MitoSV-seq method which shows 60% heteroplasmy. bp: base pair. **C.** Comparison of SV and SNVs detected in *Ifnb*$^{-/-}$ (green) and *Ifnar1*$^{-/-}$ (red) DN showing common mutation hotspot. N=10 single cells.

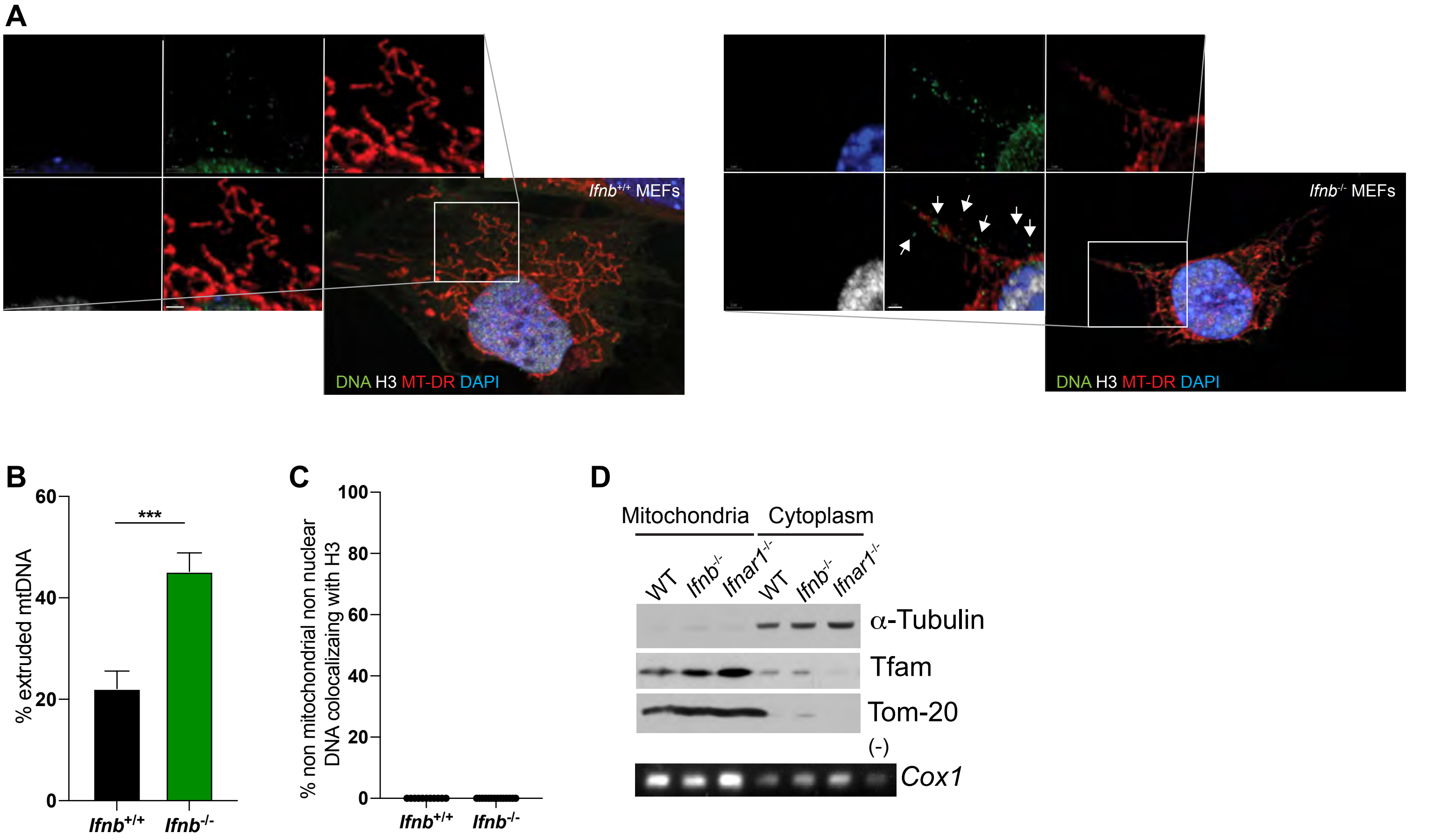

Supplementary Figure 2. mtDNA, not nuDNA, is extruded in cells lacking IFNβ. A. Immunofluorescence anti-H3 (pseudocoloured white), MitoTracker Deep Red (MT-DR) (mitochondria, pseudocoloured red) and DNA (green) in *Ifnb*$^{+/+}$ or *Ifnb*$^{-/-}$ MEFs. Non-nuclear-non-mitochondrial DNA foci are indicated with white arrows. Scale bars represent 10 um. **B.** Quantification of the percentage of extruded mtDNA as non-nuclear non-mitochondrial DNA. N=3, at least 12 cells were quantified per condition. **C.** % of non-mitochondrial, non-nuclear DNA colocalizaing with H3. N=3 at least 12 cells were quantified per condition. **D.** Cell fractionation of wild-type, *Ifnb*$^{-/-}$ or *Ifnar1*$^{-/-}$ CNs DIV6, followed by immunoblotting (upper panel) and matching PCR against mtDNA gene *CoxI*. For all graphs, data represents mean ± SEM. * means p<0.05, ** p<0.01 and *** p<0.001 by unpaired t-test.

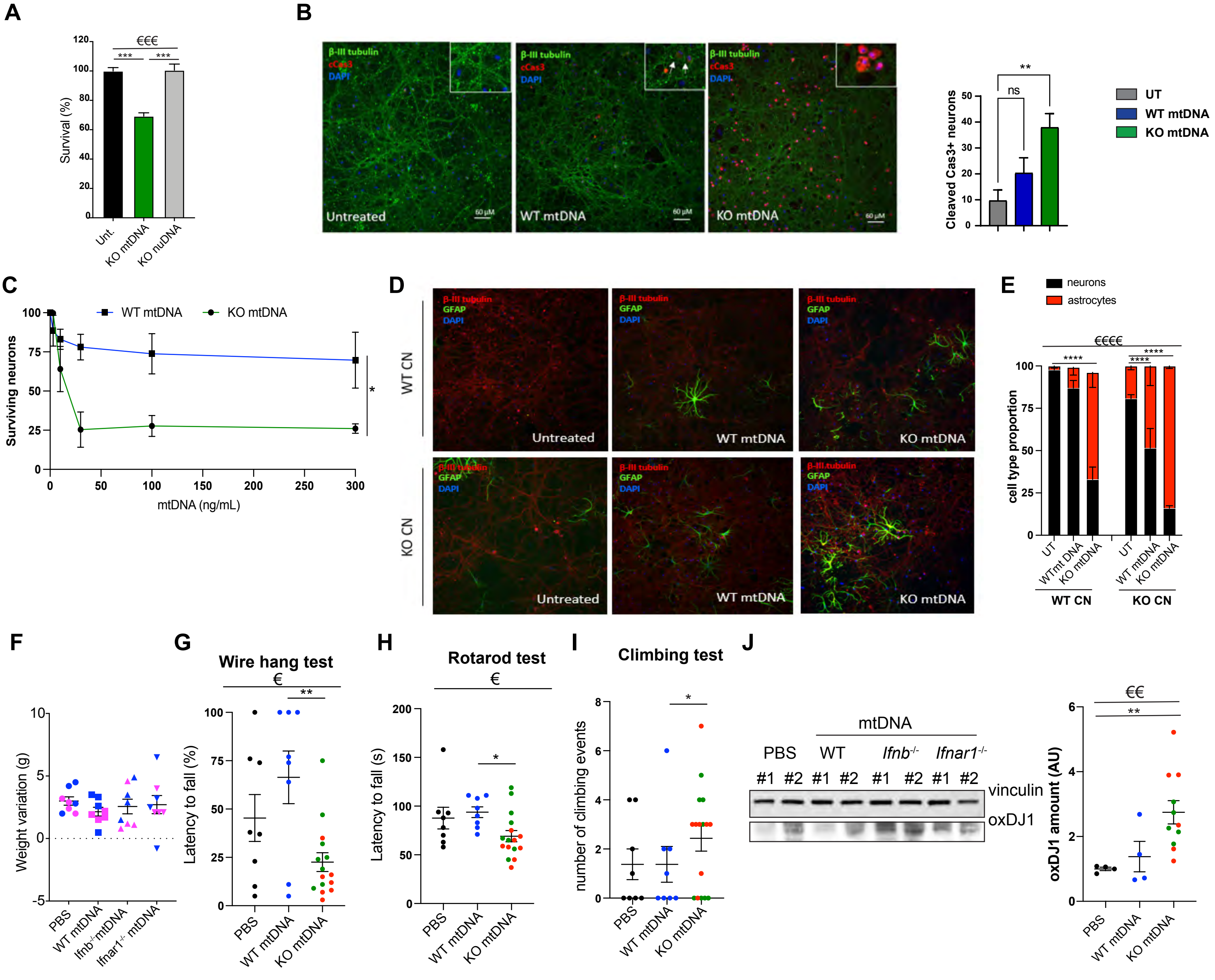

Supplementary Figure 3. Damaged mtDNA is neurotoxic. A. Survival estimated on differentiated N2A cells after treatment with either 100ng/mL of KO mtDNA or KO nuDNA. N=3. **B.** Immunofluorescence of WT CNs treated with 30 ng/mL of WT mtDNA or *Ifnb*$^{-/-}$ mtDNA for 24 h stained for cleaved Caspase3 (red), βIII tubuling (green, neurons) and DAPI (nuclei). Followed by quantification of % cleaved Caspase3+. N=3. **C.** Dose-dependent survival curve upon mtDNA treatment with WT mtDNA or *Ifnb*$^{-/-}$ mtDNA in wild-type CN for 24h. N=3. Statistics are calculated from the AUC. **D.** Immunofluorescence of WT and *Ifnb*$^{-/-}$ CNs, both treated with WT mtDNA or *Ifnb*$^{-/-}$ mtDNA for 24 h and stained for GFAP (green, astrocytes), βIII tubuling (red, neurons) and DAPI. **E.** Proportion of neurons and astrocytes in neuronal culture treated with 30ng/ml of either WT mtDNA or KO mtDNA. N=3. €€€€ p<0.0001 by two-way ANOVA. **F-I.** Complementary information on behaviour and cognitive tests presented in Figure 3. **F.** Weight monitoring of mice injected with either PBS (black, N=8), WT mtDNA (blue, N=8), *Ifnb*$^{-/-}$ mtDNA (green, N=8) or *Ifnar1*$^{-/-}$ mtDNA (red, N=8). Pointy line represents no variation of weight between day of injection and ending point. Females are indicated in pink and males in blue. **G.** Wire hang test. **H.** Rotarod 3rd (peak) trial extracted from shown in Figure 3. **I.** Climbing test. **J.** Immunoblotting of oxDJ1 and quantification. Vinculin was used as a loading control.

For all animal experiment graphs, 1 dot means 1 individual animal. PBS is shown in black, WT mtDNA in blue, and KO mtDNA in green (*Ifnb*$^{-/-}$ mtDNA) and red (*Ifnar1*$^{-/-}$ mtDNA), N=8-16/group. € means p<0.05, €€ p<0.01, €€€ p<0.001, and €€€€ p<0.0001 ordinary one-way ANOVA Krustal-Wallis ANOVA if distribution did not show Gaussian distribution (Shapiro-Wilk test) or Brown-Forsythe ANOVA if distribution showed SD differences (Bartlett's test). * means p<0.05, ** p<0.01, *** p<0.001, and **** p<0.0001 by post-hoc unpaired t-test.

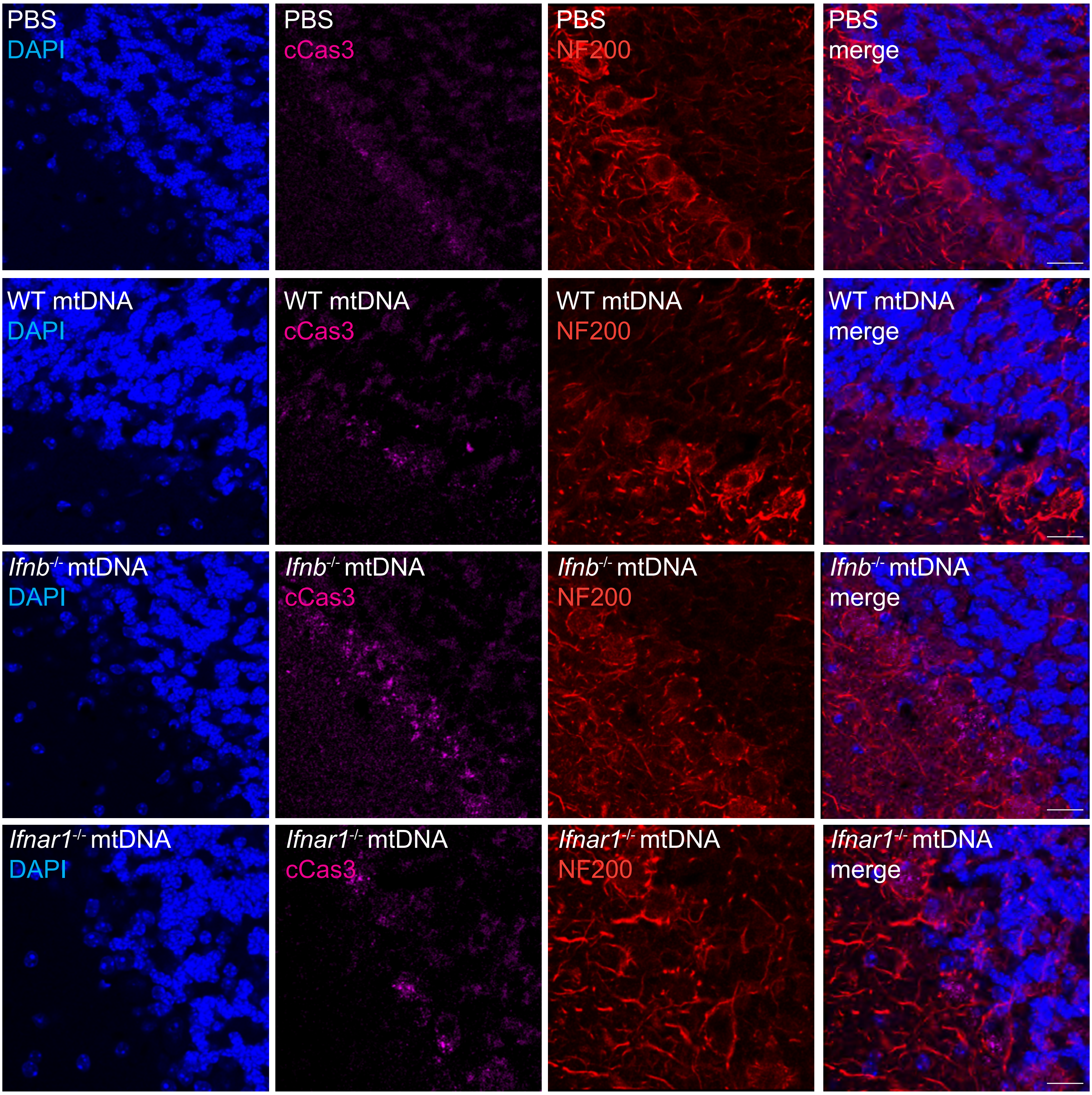

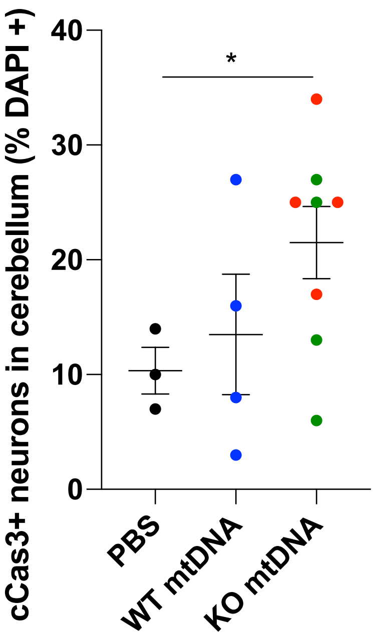

Supplementary Figure 4. Damaged mtDNA induced neurotoxicity spread in the cerebellum. Immunofluorescence for cCas3 (pink), NF200 (red, neurons) and DAPI (blue, nuclei) in cerebellum from mice injected with either PBS, WT mtDNA, *Ifnb*$^{-/-}$ mtDNA or *Ifnar1*$^{-/-}$ mtDNA. Scale bars equal 50 microns. N=4-8.
* means $p<0.05$ by unpaired t-test. PBS is shown in black, WT mtDNA in blue, and KO mtDNA in green (*Ifnb*$^{-/-}$ mtDNA) and red (*Ifnar1*$^{-/-}$ mtDNA).

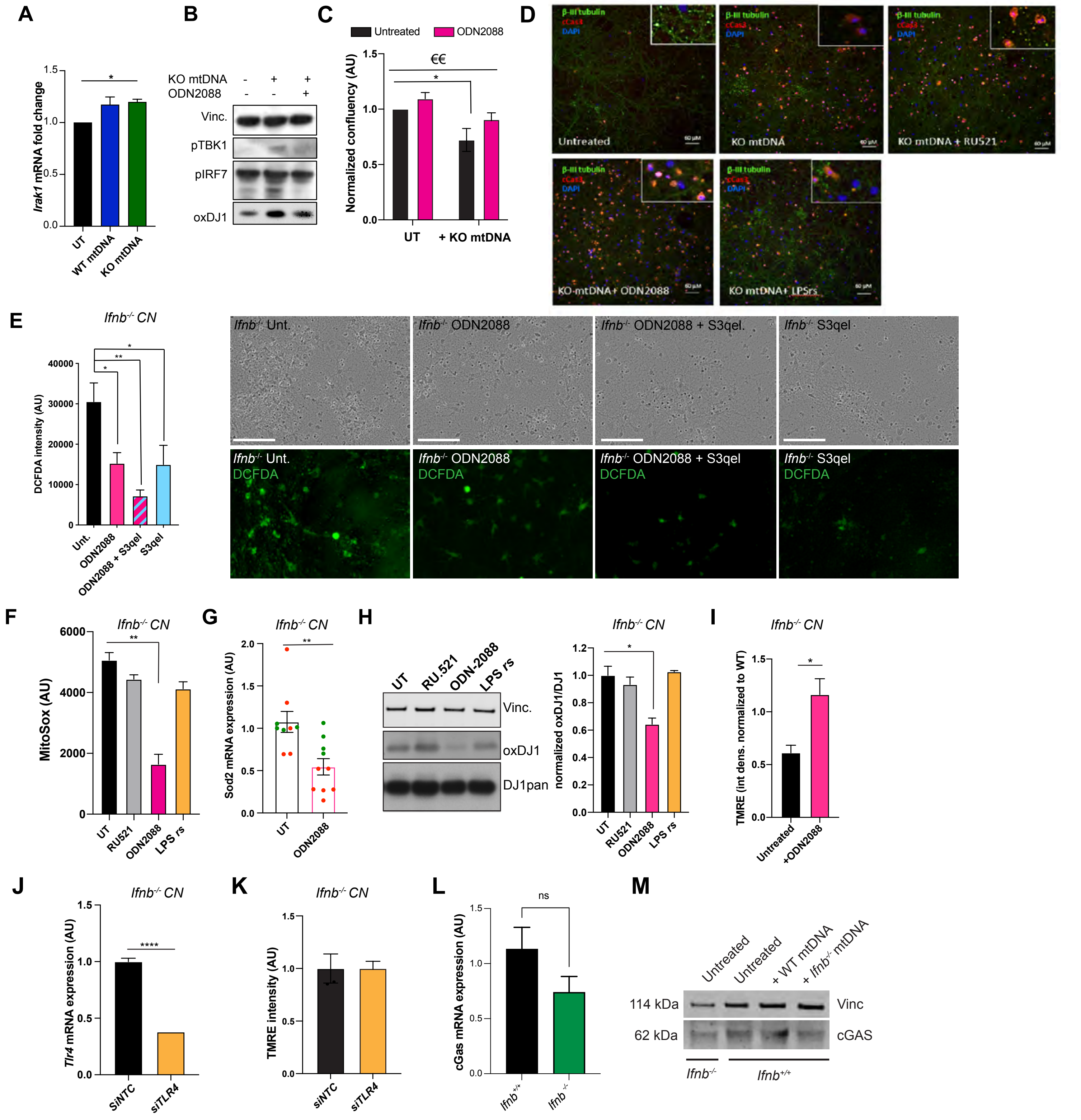

Supplementary Figure 5. Inhibition of TLR9 reverses the damaged mtDNA-induced oxidative stress in *Ifnb*$^{-/-}$ neurons. A. qPCR for *Irak1* after 24 h treatment with WT or *Ifnb*$^{-/-}$ mtDNA in WT CN. N=3. **B.** Immunoblotting for TLR9 downstream signaling proteins, pTBK1, pIRF7 and oxDJ1.3 **C.** Confluency of WT neurons after mtDNA treatment with or without the presence of ODN2088. N=3. **D.** Representative images of cCas3 (red) fluorescent staining in WT neurons treated with 30 ng/mL KO mtDNA + inhibitors: RU521, ODN2088 and LPSrs for 24h. Quantification in Figure 5H. Nuclei shown in blue (DAPI), neurons labelled in green (βIII-tubulin). Scale bars equal 60 microns. **E.** DCFDA quantification with or without ODN2088. S3qel, a selective suppressor of superoxide production from mitochondrial complex III, was used as a control. N=3. Phase and green fluorescence (DCFDA) representative images. Scale bars represent 100 µm. **F.** Mitochondrial ROS amount assessed by MitoSOX intensity after excluding nuclear staining, in *Ifnb*$^{-/-}$ CN with or without treatment with RU521, LPS rs or ODN2088. N=3/triplicates. **G.** *Sod2* expression in *Ifnb*$^{-/-}$ (green) and *Ifnar1*$^{-/-}$ (red) with or without treatment with ODN2088. N=9. **H.** oxDJ1 immunoblot in *Ifnb*$^{-/-}$ CN with or without treatment with RU521, LPS rs or ODN2088. **I.** Impact of ODN2088 on membrane potential in *Ifnb*$^{-/-}$ CN using TMRE staining. N=3. **J.** *Tlr4* mRNA expression upon TLR4 knock-down in *Ifnb*$^{-/-}$ CN. One representative of 3 biological replicates. **K.** Intensity of TMRE staining in *Ifnb*$^{-/-}$ CN with or without knock-down of TLR4. N=5. **L.** qPCR for *cGas* mRNA expression in *Ifnb*$^{+/+}$ and *Ifnb*$^{-/-}$ CN. N=5. **M.** Immunoblotting for cGAS protein amount in *Ifnb*$^{-/-}$ CN and *Ifnb*$^{+/+}$ treated with WT or *Ifnb*$^{-/-}$ mtDNA.

Data represent mean ± SEM. If two groups are represented they were statistically analysed by unpaired T-Test, if 3 or 4 groups represented, then one-way ANOVA with multiple comparisons was used. For all graphs *p-val<0.05, ** p-val<0.01, ***p-val<0.001, ****p-val<0.0001.

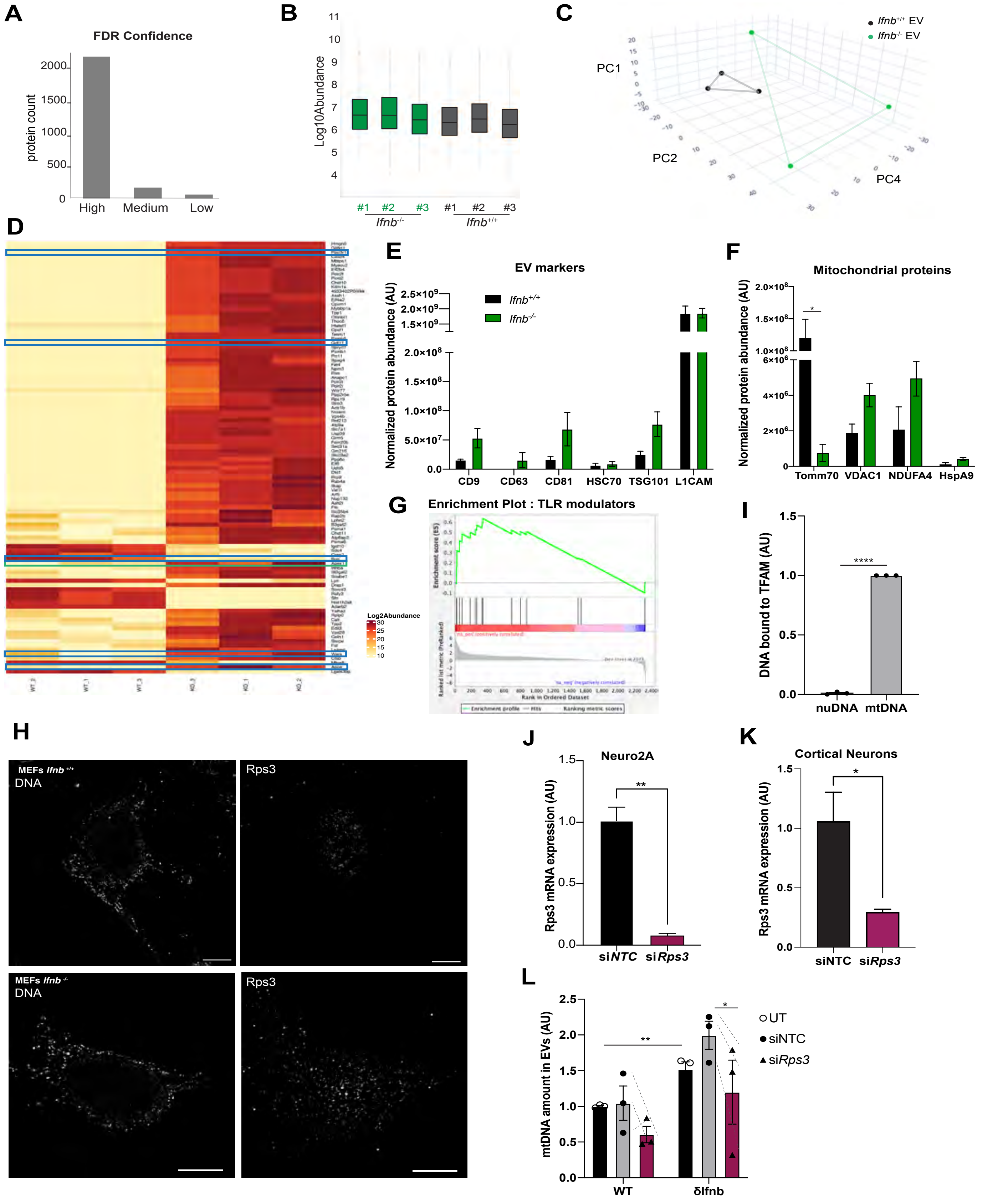

Supplementary Figure 6. Proteomics analysis revealed differential protein content of EVs purified from *Ifnb*$^{+/+}$ and *Ifnb*$^{-/-}$ CN. A. Total number of identified proteins classified according to FDR confidence in high, medium or low. **B.** Logarithmic representation of normalized abundances of individual samples coloured according to condition. **C.** 3D Principal component analysis (PCA) plot of the protein content profiles of EV from *Ifnb*$^{+/+}$ (black) and *Ifnb*$^{-/-}$ CNs. Each dot represents an individual sample. **D.** Heatmap plot of the top 100 most differential expressed proteins. **E-F.** Normalized abundances of E. EV markers; F. mitochondrial. **G.** Enrichment Plot for TLR modulators. For A-G N=3/group. **H.** Single channel of immunofluorescence staining of *Ifnb*$^{+/+}$ and *Ifnb*$^{-/-}$ MEFs for DNA and Rps3. Coloured images and quantification in Fig. 6H. **I.** Anti Tfam mtDNA immunoprecipitation used as a positive control for efficiency of mtDNA IP shown in Figure 6. One representative of 3 biological replicates. **J.** Knock-down of Rps3 in N2As validated with qPCR. **K.** Rps3 knock-down efficiency in N2As and CN. **L.** mtDNA levels in EVs purified from WT and δ*Ifnb* N2A cells knocked down for Rps3. siNTC is used as control. N=3.

For all graphs data represent mean ± SEM. Protein abundances and I-K) are analysed by unpaired T-test. L is analysed by one-way ANOVA with multiple comparisons. For all graphs *p-val<0.05, ** p-val<0.01, ***p-val<0.001, ****p-val<0.0001.

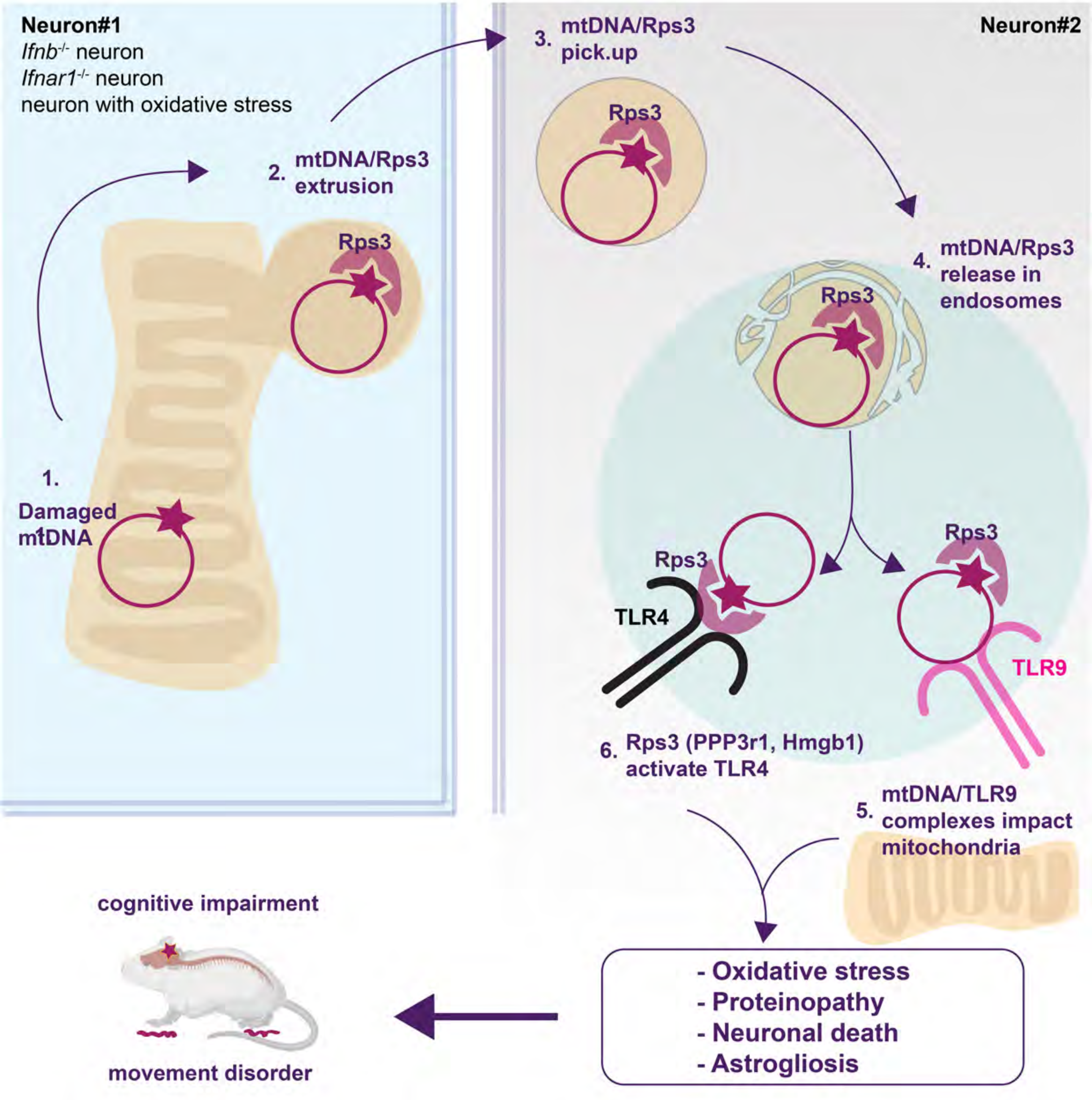

Supplementary Figure 7. Schematic model of damaged mtDNA extrusion and neurotoxicity inducing neurodegeneration and PDD-like pathology. In neurons lacking IFNβ/IFNAR signalling, subjected to oxidative stress, Rps3 binds damaged mtDNA and thereby the complexes are extruded in EVs. These EVs are picked-up by neighbouring healthy neurons and coactivate TLR9 and TLR4 pathways. The signalling pathways induce proteinopathy, neuronal death and astrogliosis, leading to neurodegeneration. The star represents oxidative damages on mtDNA.

Supplementary tables

Supplementary table 1: List of TLR modulators (activator and inhibitor proteins)

Protein name	Symbol	Uniprot ID	target	Ref	KO vs WT log2 Fold-change	KO vs WT p-value	Rank
**Calcineurin B**	Ppp3r1	Q63810	TLR4	[1]	9.92987	1.97208E-05	26
**Growth Differentiation Factor 11**	GDF11	Q9Z1W4	TLR4, TLR2	[2]	11.22797	3.1479E-05	29
**Apolipoprotein E**	ApoE	P08226	TLR3, TLR4	[3 4]	1.65056	0.000867	64
**Tryptophanyl-tRNA Synthetase 1**	WARS1	E0CXX4	TLR2, TLR4	[5]	4.17236	0.005211	100
**Endoplasmin**	Hsp90b1	P08113	TLR4, TLR2	[6]	2.80606	0.022281	208
**Nucleophosmin 1**	Npm1	Q61937	TLR4	[7]	1,93969	0,031921	278
**Ribosomal protein S3**	Rps3	P62908	TLR4	[8]	2.67281	0.034660	299
**Heatshock 70kDa**	Hsp70	P17879	TLR4, TLR2	[9]	3,17708	0,045692	376
**TLR4 interactor with leucine rich repeats**	TRIL	Q9DBY4	TLR4, TLR3	[10 11]	5.00339	0.135716	948
**Fibronectin extra domain A**	Fn1	P11276	TLR4	[12]	1.95369	0.114627	864
**High Mobility Group Box 1**	hmgb1	P63158	TLR4, TLR2, TLR5, TLR9	[13]	1.76656	0.098442	748
**60 kDa heat shock protein**	Hsp60	P63038	TLR4, TLR2, TLR5	[14]	0.94838	0.383724	1511
**Amyloid Beta**	APP	P12023	TLR2, TLR4, TLR6, TLR9	[15]	0.78691	0.044308	364
**Tenascin-C**	Tnc	Q80YX1	TLR4	[16]	0.28748	0.551466	1702
**Versican**	Vcan	Q62059	TLR2	[17]	2.42102	0.635803	1755
**Biglycan**	BGN	P28653	TLR2, TLR4	[18]	-2,70279	0.001243	72
**angiotensin II, precursor = angiotensinogen**	Agt	P11859	TLR4	[19]	NF	n/a	
**Antiphospholipid antibodies**	n/a	n/a	TLR7, TLR8	[20]	NF	n/a	
**Beta defensin-2**	BD-2	O15263	TLR4	[21]	NF	n/a	
**Fibrinogen**	Fgl1	Q71KU9	TLR4	[22]	NF	n/a	
**Hyaluronan binding protein 2**	Habp2	Q8K0D2	TLR4	[23]	NF	n/a	
**Heparan sulfate N-deacetylse**	Ndst1	Q3UHN9	TLR4	[24]	NF	n/a	
**myeloid-related protein-8**	Mrp8	P27005	TLR4	[25]	NF	n/a	
**myeloid-related protein-14**	Mrp14	P31725	TLR4	[25]	NF	n/a	
**Oxidized low-density lipoprotein receptor 1**	OLR1	Q9EQ09	TLR4	[26]	NF	n/a	
**Serum amyloid A**	SAA-1	P05366	TLR1, TLR2	[27]	NF	n/a	

Supplementary table 2: List of antibodies used.

PROTEIN	SUPPLIER	USE
**DNA**	Millipore, CBL186	WB 1:1000
**CD9**	Abcam, ab92726	WB 1:100
**Cleaved Cas3**	CST - #9661	WB 1:500, IF/IHC 1:100
**GAPDH**	Abcam, ab9484	WB 1:5000
**GFAP**	Invitrogen, 130300	IF/IHC 1:500
**DJ1**	Abcam, ab18257	IF 1:1000, WB 1:1000
**DJ1 (ox)**	Millipore, mabn1773	IF 1:100, WB 1:200
**dGuanine (8hydroxy-)**	Santa-Cruz, sc-139586	IF 1:100
**Hsp60**	Santa-Cruz, sc-1052; ENZO ADI-SPA-828	IF 1:100 both
**H3**	CST, #4499	IF 1:400
**Iba1**	WAKO, 019 19741	IHC 1:500
**Irak1**	CST, #4504	WB 1:500
**Irf7 (pS471/472)**	Biorbyt, orb6233	WB, 1:200
**Nd4**	Novus, NBP2-93572	WB 1:500
**Nd5**	Abcam, ab230772	WB, 1:500
**NF200**	Abcam -ab4680	IHC : 1:2000
**Rps3**	CST, #9538	WB 1:500, IF 1:60
**Synuclein (pan)**	BD bioscience, 610786	WB 1:500
**Synuclein (pS129)**	Bioss, bs-5628R	IHC 1:100; WB 1:500
**Tau (pT205)**	Abcam, ab4841	WB, 1:500
**TBK1 (pS172)**	CST, #5483	WB 1:500
**TH**	Pel-Freeze, P40101	IHC 1:1000
**TLR4**	Santa-Cruz, sc-293072; Thermo, 48-2300	WB 1:500
**Tom20**	Santa-Cruz, sc-11415; abcam, ab186734	IF 1:100; WB : 1:1000
**a-Tubulin**	Sigma - T9026	WB 1:10 000
**□3-Tubulin**	Santa-Cruz - sc-58888	IF 1:1000
**Vinculin**	Sigma - V9131	WB : 1:10000

Supplementary table 3: List of primers used for qPCR

Primer names	Sequence
**Actb forward primer**	5'-CTGTGTGGATTGGTGGCTTCTAT-3'
**Actb reverse primer**	5'-GTAACAGTCCGCCTAGAAGCAT-3'
**D-loop forward primer**	5’-AATCTACCATCCTCCGTGAAACC-3’
**D-loop reverse primer**	5’-TCAGTTTAGCTACCCCCAAGTTTAA-3’
**Gapdh forward primer**	5'-AGGTCGGTGTGAACGGATTTG-3'
**Gapdh reverse primer**	5'-TGTAGACCATGTAGTTGAGGTCA-3'
**cGAS forward primer**	5’- GTCGGAGTTCAAAGGTGTGGA -3’
**cGAS reverse primer**	5’- GACTCAGCGGATTTCCTCGTG -3’
**Irak1 forward primer**	5’- GAGAGTGTTCCTGGCCTCTC -3’
**Irak1 reverse primer**	5’- GCTGGGTTGATGATGATCTG -3’
**mt-Nd4 forward primer**	5’-AACGGATCCACAGCCGTA-3’
**mt-Nd4 reverse primer**	5’-AGTCCTCGGGCCCATGATT-3’
**Rps3 forward primer**	5’-AAGATGGCGGTG-CAGATTTC-3’
**Rps3 reverse primer**	5’-AGTAGCCATCTTCAGCCAGCTC-3’
**TLR4 forward primer**	5’-CAACATCATCCAGGAAGGC-3’
**TLR4 reverse primer**	5’-GAAGGCGATACAATTCCACC-3’
**TLR9 forward primer**	5’-CAAGAACCTGGTGTCACTGC-3’
**TLR9 reverse primer**	5’-TGCGATTGTCTGACAAGTCC-3’

Supplementary table 4: KEGG pathways negatively dysregulated in sporadic PDD

Probe	SIZE	ES	NES	NOM p-val	FDR q-val
**Kegg_Oxidative_Phosphorylation**	110	-0.3873	-4.644.161	00.00	00.00
**Kegg_Parkinsons_Disease**	106	-0.3840	-4.558.958	00.00	00.00
**Kegg_Proteasome**	42	-0.5567	-4.344.569	00.00	00.00
**Kegg_Spliceosome**	96	-0.3849	-43.284.864	00.00	00.00
**Kegg_Huntingtons_Disease**	164	-0.2330	-35.427.723	00.00	00.00
**Kegg_Alzheimers_Disease**	149	-0.2404	-34.297.092	00.00	00.00
**Kegg_Citrate_Cycle_Tca_Cycle**	29	-0.4970	-31.964.567	00.00	00.00
**Kegg_Ubiquitin_Mediated_Proteolysis**	122	-0.2316	-30.058.653	00.00	00.00
**Kegg_Protein_Export**	20	-0.5359	-2.857.628	00.00	00.00
**Kegg_Aminoacyl_Trna_Biosynthesis**	32	-0.4203	-28.393.216	00.00	00.00
**Kegg_Valine_Leucine_And_Isoleucine_Degradation**	43	-0.3279	-2.585.012	00.00	6,50E+02
**Kegg_Nucleotide_Excision_Repair**	44	-0.3266	-2.532.493	00.00	5,95E+02
**Kegg_Butanoate_Metabolism**	31	-0.3605	-23.957.264	00.00	0.0023
**Kegg_Pyruvate_Metabolism**	40	-0.3051	-2.265.731	00.00	0.0050
**Kegg_N_Glycan_Biosynthesis**	42	-0.2960	-22.004.862	0.0019	0.0072
**Kegg_Alanine_Aspartate_And_Glutamate_Metabolism**	32	-0.3209	-21.988.769	0.0019	0.0068
**Kegg_Mismatch_Repair**	22	-0.39452	-21.945.817	00.00	0.0064
**Kegg_Glycosylphosphatidylinositol_Gpi_Anchor_Biosynthesis**	24	-0.3588	-21.606.119	00.00	0.0080
**Kegg_Propanoate_Metabolism**	31	-0.3213	-2.119.608	0.0062	0.0095
**Kegg_Peroxisome**	72	-0.2110	-2.063.174	0.0040	0.0132
**Kegg_Terpenoid_Backbone_Biosynthesis**	15	-0.4201	-19.836.801	0.0081	0.0213

Supplementary table 5: Genes in KEGG pathway of Oxidative Phosphorylation in sporadic PDD

NAME	PROBE	GENE SYMBOL	GENE_TITLE	RANK IN GENE LIST	RANK METRIC SCORE	RUNNING ES	CORE ENRICHMENT
**row_0**	COX6A2	COX6A2	cytochrome c oxidase subunit VIa polypeptide 2	477	0.6380963921546936	-0.0141	No
**row_1**	COX10	COX10	COX10 homolog, cytochrome c oxidase assembly protein, heme A: farnesyltransferase (yeast)	859	0.5711776614189148	-0.023680009	No
**row_2**	ATP6V0D2	ATP6V0D2	ATPase, H+ transporting, lysosomal 38kDa, V0 subunit d2	1814	0.48437973856925964	-0.061134767	No
**row_3**	COX4I2	COX4I2	cytochrome c oxidase subunit IV isoform 2 (lung)	2043	0.46991807222366333	-0.06316798	No
**row_4**	ATP6V0A4	ATP6V0A4	ATPase, H+ transporting, lysosomal V0 subunit a4	2540	0.441964328289032	-0.07827692	No
**row_5**	ATP5E	ATP5E	ATP synthase, H+ transporting, mitochondrial F1 complex, epsilon subunit	2546	0.44183164834976196	-0.06942996	No
**row_6**	LHPP	LHPP	-	2883	0.42520201206207275	-0.07673249	No
**row_7**	COX6A1	COX6A1	cytochrome c oxidase subunit VIa polypeptide 1	3580	0.39432963728904724	-0.101599425	No
**row_8**	ATP6V1G3	ATP6V1G3	ATPase, H+ transporting, lysosomal 13kDa, V1 subunit G3	4169	0.3692993223667145	-0.12119704	No
**row_9**	ATP12A	ATP12A	ATPase, H+/K+ transporting, nongastric, alpha polypeptide	4640	0.35156872868537903	-0.13503744	No
**row_10**	NDUFS8	NDUFS8	NADH dehydrogenase (ubiquinone) Fe-S protein 8, 23kDa (NADH-coenzyme Q reductase)	4836	0.3437674343585968	-0.13546057	No
**row_11**	NDUFA3	NDUFA3	NADH dehydrogenase (ubiquinone) 1 alpha subcomplex, 3, 9kDa	5223	0.3286764919757843	-0.1452026	No
**row_12**	COX6B2	COX6B2	cytochrome c oxidase subunit VIb polypeptide 2 (testis)	5605	0.3152139186859131	-0.1547007	No
**row_13**	TCIRG1	TCIRG1	T-cell, immune regulator 1, ATPase, H+ transporting, lysosomal V0 subunit A3	5933	0.30455389618873596	-0.16156411	No
**row_14**	ATP6V1E2	ATP6V1E2	ATPase, H+ transporting, lysosomal 31kDa, V1 subunit E2	6516	0.2841726541519165	-0.180869	No
**row_15**	ATP6V0A1	ATP6V0A1	ATPase, H+ transporting, lysosomal V0 subunit a1	7242	0.2600027620792389	-0.20715085	No
**row_16**	NDUFB8	NDUFB8	NADH dehydrogenase (ubiquinone) 1 beta subcomplex, 8, 19kDa	8742	0.20852456986904144	-0.27119616	No
**row_17**	ATP4A	ATP4A	ATPase, H+/K+ exchanging, alpha polypeptide	9044	0.19777773320674896	-0.27679104	No
**row_18**	ATP6V0A2	ATP6V0A2	ATPase, H+ transporting, lysosomal V0 subunit a2	9050	0.19750310480594635	-0.26794407	No
**row_19**	COX7B2	COX7B2	cytochrome c oxidase subunit VIIb2	9128	0.19502145051956177	-0.26261	No
**row_20**	ATP6V0B	ATP6V0B	ATPase, H+ transporting, lysosomal 21kDa, V0 subunit b	9617	0.176547110080719	-0.2773286	No
**row_21**	NDUFB10	NDUFB10	NADH dehydrogenase (ubiquinone) 1 beta subcomplex, 10, 22kDa	9832	0.1688237190246582	-0.27867877	No
**row_22**	ATP6V0C	ATP6V0C	ATPase, H+ transporting, lysosomal 16kDa, V0 subunit c	10924	0.12755228579044342	-0.32281774	No
**row_23**	NDUFA11	NDUFA11	NADH dehydrogenase (ubiquinone) 1 alpha subcomplex, 11, 14.7kDa	11155	0.11898084729909897	-0.32494855	No
**row_24**	ATP6V1B1	ATP6V1B1	ATPase, H+ transporting, lysosomal 56/58kDa, V1 subunit B1 (Renal tubular acidosis with deafness)	11385	0.11042555421590805	-0.32703054	No
**row_25**	ATP4B	ATP4B	ATPase, H+/K+ exchanging, beta polypeptide	11611	0.10110900551080704	-0.32891738	No

**row_26**	COX15	COX15	COX15 homolog, cytochrome c oxidase assembly protein (yeast)	12031	0.082719236 61231995	-0.3402695	No
**row_27**	NDUFS7	NDUFS7	NADH dehydrogenase (ubiquinone) Fe-S protein 7, 20kDa (NADH-coenzyme Q reductase)	12143	0.077764086 42530441	-0.33659428	No
**row_28**	COX7B	COX7B	cytochrome c oxidase subunit VIIb	12661	0.055236466 228961945	-0.3527278	No
**row_29**	COX11	COX11	COX11 homolog, cytochrome c oxidase assembly protein (yeast)	12907	0.044104564 93496895	-0.35559043	No
**row_30**	ATP5I	ATP5I	ATP synthase, H+ transporting, mitochondrial F0 complex, subunit E	12949	0.042038265 615701675	-0.34849992	No
**row_31**	ATP5G2	ATP5G2	ATP synthase, H+ transporting, mitochondrial F0 complex, subunit C2 (subunit 9)	13032	0.038504354 655742645	-0.3434098	No
**row_32**	ATP6V1C2	ATP6V1C2	ATPase, H+ transporting, lysosomal 42kDa, V1 subunit C2	13358	0.024314811 45322323	-0.35017565	No
**row_33**	COX5B	COX5B	cytochrome c oxidase subunit Vb	13498	0.018004901 707172394	-0.34786654	No
**row_34**	COX7A1	COX7A1	cytochrome c oxidase subunit VIIa polypeptide 1 (muscle)	14024	- 0.007189299 911260605	-0.3643904	No
**row_35**	NDUFB7	NDUFB7	NADH dehydrogenase (ubiquinone) 1 beta subcomplex, 7, 18kDa	14322	- 0.023781195 282936096	-0.3697901	No
**row_36**	ATP5D	ATP5D	ATP synthase, H+ transporting, mitochondrial F1 complex, delta subunit	14435	- 0.029495235 5325222	-0.36616367	No
**row_37**	COX8C	COX8C	cytochrome c oxidase subunit 8C	14871	- 0.051302887 49933243	-0.37829643	Yes
**row_38**	ATP5J2	ATP5J2	ATP synthase, H+ transporting, mitochondrial F0 complex, subunit F2	14881	- 0.051924023 777246475	-0.36964464	Yes
**row_39**	COX7A2L	COX7A2L	cytochrome c oxidase subunit VIIa polypeptide 2 like	15066	- 0.062114436 17939949	-0.3695311	Yes
**row_40**	PPA1	PPA1	pyrophosphatase (inorganic) 1	15128	- 0.064905077 2190094	-0.36341637	Yes
**row_41**	ATP6V1A	ATP6V1A	ATPase, H+ transporting, lysosomal 70kDa, V1 subunit A	15134	- 0.065126620 23305893	-0.3545694	Yes
**row_42**	NDUFB9	NDUFB9	NADH dehydrogenase (ubiquinone) 1 beta subcomplex, 9, 22kDa	15184	- 0.068603150 54655075	-0.34786922	Yes
**row_43**	COX7C	COX7C	cytochrome c oxidase subunit VIIc	15236	- 0.072168357 67030716	-0.3412666	Yes
**row_44**	COX6B1	COX6B1	cytochrome c oxidase subunit Vib polypeptide 1 (ubiquitous)	15322	- 0.076806917 7865982	-0.33632284	Yes
**row_45**	UQCRQ	UQCRQ	ubiquinol-cytochrome c reductase, complex III subunit VII, 9.5kDa	15429	- 0.082984261 21473312	-0.33240366	Yes
**row_46**	NDUFB6	NDUFB6	NADH dehydrogenase (ubiquinone) 1 beta subcomplex, 6, 17kDa	15526	- 0.088709272 4442482	-0.32799658	Yes
**row_47**	COX7A2	COX7A2	cytochrome c oxidase subunit VIIa polypeptide 2 (liver)	15528	- 0.088837563 99154663	-0.31895447	Yes
**row_48**	NDUFA4L2	NDUFA4L2	NADH dehydrogenase (ubiquinone) 1 alpha subcomplex, 4-like 2	15597	- 0.093455903 23209763	-0.31318128	Yes
**row_49**	NDUFV3	NDUFV3	NADH dehydrogenase (ubiquinone) flavoprotein 3, 10kDa	15929	- 0.115533672 27315903	-0.32023987	Yes
**row_50**	COX8A	COX8A	cytochrome c oxidase subunit 8A (ubiquitous)	15976	- 0.118415772 91488647	-0.3133933	Yes

**row_51**	NDUFA8	NDUFA8	NADH dehydrogenase (ubiquinone) 1 alpha subcomplex, 8, 19kDa	16013	-0.12040300667285919	-0.30605882	Yes
**row_52**	NDUFA4	NDUFA4	NADH dehydrogenase (ubiquinone) 1 alpha subcomplex, 4, 9kDa	16172	-0.13011720776557922	-0.30467674	Yes
**row_53**	UQCRC1	UQCRC1	ubiquinol-cytochrome c reductase core protein I	16516	-0.15236227214336395	-0.3123208	Yes
**row_54**	SDHD	SDHD	succinate dehydrogenase complex, subunit D, integral membrane protein	17035	-0.18738345801830292	-0.32850313	Yes
**row_55**	COX5A	COX5A	cytochrome c oxidase subunit Va	17107	-0.19382213056087494	-0.3228763	Yes
**row_56**	NDUFAB1	NDUFAB1	NADH dehydrogenase (ubiquinone) 1, alpha/beta subcomplex, 1, 8kDa	17246	-0.20355217158794403	-0.3205184	Yes
**row_57**	ATP6V1E1	ATP6V1E1	ATPase, H+ transporting, lysosomal 31kDa, V1 subunit E1	17247	-0.20361901819705963	-0.3114275	Yes
**row_58**	CYC1	CYC1	cytochrome c-1	17410	-0.21724553406238556	-0.3102406	Yes
**row_59**	COX17	COX17	COX17 cytochrome c oxidase assembly homolog (S. cerevisiae)	17435	-0.21917259693145752	-0.30232063	Yes
**row_60**	ATP5G1	ATP5G1	ATP synthase, H+ transporting, mitochondrial F0 complex, subunit C1 (subunit 9)	17493	-0.22411076724529266	-0.29601076	Yes
**row_61**	NDUFA9	NDUFA9	NADH dehydrogenase (ubiquinone) 1 alpha subcomplex, 9, 39kDa	17642	-0.23691420257091522	-0.29414076	Yes
**row_62**	ATP6V1D	ATP6V1D	ATPase, H+ transporting, lysosomal 34kDa, V1 subunit D	17661	-0.23940809071063995	-0.28592807	Yes
**row_63**	NDUFA2	NDUFA2	NADH dehydrogenase (ubiquinone) 1 alpha subcomplex, 2, 8kDa	17711	-0.2448466718196869	-0.27922788	Yes
**row_64**	ATP6V1B2	ATP6V1B2	ATPase, H+ transporting, lysosomal 56/58kDa, V1 subunit B2	17806	-0.25358930230140686	-0.27472323	Yes
**row_65**	NDUFA6	NDUFA6	NADH dehydrogenase (ubiquinone) 1 alpha subcomplex, 6, 14kDa	17848	-0.2567613422870636	-0.26763272	Yes
**row_66**	ATP6V0D1	ATP6V0D1	ATPase, H+ transporting, lysosomal 38kDa, V0 subunit d1	17886	-0.2593167722225189	-0.26034704	Yes
**row_67**	ATP6V1G2	ATP6V1G2	ATPase, H+ transporting, lysosomal 13kDa, V1 subunit G2	17969	-0.26702532172203064	-0.25525692	Yes
**row_68**	UQCRFS1	UQCRFS1	ubiquinol-cytochrome c reductase, Rieske iron-sulfur polypeptide 1	18077	-0.27712565660476685	-0.25138652	Yes
**row_69**	UQCRC2	UQCRC2	ubiquinol-cytochrome c reductase core protein II	18121	-0.28011181950569153	-0.24439359	Yes
**row_70**	NDUFS5	NDUFS5	NADH dehydrogenase (ubiquinone) Fe-S protein 5, 15kDa (NADH-coenzyme Q reductase)	18137	-0.2820643484592438	-0.23603453	Yes
**row_71**	SDHA	SDHA	succinate dehydrogenase complex, subunit A, flavoprotein (Fp)	18191	-0.2862662374973297	-0.2295295	Yes
**row_72**	NDUFB5	NDUFB5	NADH dehydrogenase (ubiquinone) 1 beta subcomplex, 5, 16kDa	18315	-0.29817628860473633	-0.22643976	Yes
**row_73**	NDUFV1	NDUFV1	NADH dehydrogenase (ubiquinone) flavoprotein 1, 51kDa	18317	-0.29824551939964294	-0.21739763	Yes
**row_74**	ATP6V1F	ATP6V1F	ATPase, H+ transporting, lysosomal 14kDa, V1 subunit F	18498	-0.3150407671928406	-0.21708892	Yes
**row_75**	PPA2	PPA2	pyrophosphatase (inorganic) 2	18514	-0.3170350193977356	-0.20872986	Yes

**row_76**	ATP5C1	ATP5C1	ATP synthase, H+ transporting, mitochondrial F1 complex, gamma polypeptide 1	18525	-0.31772103905677795	-0.20012686	Yes
**row_77**	NDUFB2	NDUFB2	NADH dehydrogenase (ubiquinone) 1 beta subcomplex, 2, 8kDa	18532	-0.3181929290294647	-0.19132869	Yes
**row_78**	ATP5B	ATP5B	ATP synthase, H+ transporting, mitochondrial F1 complex, beta polypeptide	18567	-0.32335686683654785	-0.18389665	Yes
**row_79**	ATP6V1C1	ATP6V1C1	ATPase, H+ transporting, lysosomal 42kDa, V1 subunit C1	18571	-0.32373589277267456	-0.1749521	Yes
**row_80**	ATP5F1	ATP5F1	ATP synthase, H+ transporting, mitochondrial F0 complex, subunit B1	18616	-0.3293675482273102	-0.16800795	Yes
**row_81**	ATP6V1H	ATP6V1H	ATPase, H+ transporting, lysosomal 50/57kDa, V1 subunit H	18727	-0.3420751094818115	-0.16428395	Yes
**row_82**	COX6C	COX6C	cytochrome c oxidase subunit VIc	18728	-0.34214159846305847	-0.15519305	Yes
**row_83**	NDUFA5	NDUFA5	NADH dehydrogenase (ubiquinone) 1 alpha subcomplex, 5, 13kDa	18798	-0.3484514355659485	-0.14946865	Yes
**row_84**	NDUFS6	NDUFS6	NADH dehydrogenase (ubiquinone) Fe-S protein 6, 13kDa (NADH-coenzyme Q reductase)	18805	-0.34929755330085754	-0.14067048	Yes
**row_85**	ATP5J	ATP5J	ATP synthase, H+ transporting, mitochondrial F0 complex, subunit F6	18858	-0.35540473461151123	-0.13411665	Yes
**row_86**	ATP5A1	ATP5A1	ATP synthase, H+ transporting, mitochondrial F1 complex, alpha subunit 1, cardiac muscle	18878	-0.357760488986969	-0.12595275	Yes
**row_87**	NDUFA7	NDUFA7	NADH dehydrogenase (ubiquinone) 1 alpha subcomplex, 7, 14.5kDa	18928	-0.363879531621933	-0.11925255	Yes
**row_88**	NDUFS4	NDUFS4	NADH dehydrogenase (ubiquinone) Fe-S protein 4, 18kDa (NADH-coenzyme Q reductase)	18930	-0.36420780420303345	-0.110210426	Yes
**row_89**	NDUFS3	NDUFS3	NADH dehydrogenase (ubiquinone) Fe-S protein 3, 30kDa (NADH-coenzyme Q reductase)	19047	-0.37942391633987427	-0.10677916	Yes
**row_90**	UQCRH	UQCRH	ubiquinol-cytochrome c reductase hinge protein	19053	-0.3803313374519348	-0.097932205	Yes
**row_91**	ATP6V1G1	ATP6V1G1	ATPase, H+ transporting, lysosomal 13kDa, V1 subunit G1	19098	-0.38597771525382996	-0.090988055	Yes
**row_92**	NDUFS2	NDUFS2	NADH dehydrogenase (ubiquinone) Fe-S protein 2, 49kDa (NADH-coenzyme Q reductase)	19173	-0.39477768540382385	-0.0855076	Yes
**row_93**	ATP5G3	ATP5G3	ATP synthase, H+ transporting, mitochondrial F0 complex, subunit C3 (subunit 9)	19190	-0.3984123468399048	-0.077197336	Yes
**row_94**	NDUFB3	NDUFB3	NADH dehydrogenase (ubiquinone) 1 beta subcomplex, 3, 12kDa	19239	-0.40455251932144165	-0.07044835	Yes
**row_95**	SDHB	SDHB	succinate dehydrogenase complex, subunit B, iron sulfur (Ip)	19289	-0.41164103150367737	-0.06374815	Yes
**row_96**	ATP6AP1	ATP6AP1	ATPase, H+ transporting, lysosomal accessory protein 1	19375	-0.423543781042099	-0.05880439	Yes
**row_97**	COX4I1	COX4I1	cytochrome c oxidase subunit IV isoform 1	19387	-0.42700624465942383	-0.050250173	Yes
**row_98**	NDUFB1	NDUFB1	NADH dehydrogenase (ubiquinone) 1 beta subcomplex, 1, 7kDa	19476	-0.44196099042892456	-0.045452785	Yes

**row_99**	NDUFA1	NDUFA1	NADH dehydrogenase (ubiquinone) 1 alpha subcomplex, 1, 7.5kDa	19486	-0.4441453218460083	-0.036800984	Yes
**row_100**	NDUFC1	NDUFC1	NADH dehydrogenase (ubiquinone) 1, subcomplex unknown, 1, 6kDa	19607	-0.4638125002384186	-0.033564877	Yes
**row_101**	NDUFV2	NDUFV2	NADH dehydrogenase (ubiquinone) flavoprotein 2, 24kDa	19759	-0.49130919575691223	-0.03184126	Yes
**row_102**	NDUFA10	NDUFA10	NADH dehydrogenase (ubiquinone) 1 alpha subcomplex, 10, 42kDa	19765	-0.4936276376247406	-0.0229943	Yes
**row_103**	NDUFC2	NDUFC2	NADH dehydrogenase (ubiquinone) 1, subcomplex unknown, 2, 14.5kDa	19893	-0.5258064866065979	-0.020099722	Yes
**row_104**	ATP5L	ATP5L	ATP synthase, H+ transporting, mitochondrial F0 complex, subunit G	20066	-0.5752218961715698	-0.019400695	Yes
**row_105**	ATP5H	ATP5H	ATP synthase, H+ transporting, mitochondrial F0 complex, subunit d	20162	-0.6046980619430542	-0.014944837	Yes
**row_106**	NDUFS1	NDUFS1	NADH dehydrogenase (ubiquinone) Fe-S protein 1, 75kDa (NADH-coenzyme Q reductase)	20203	-0.6192063689231873	-0.0078055286	Yes
**row_107**	SDHC	SDHC	succinate dehydrogenase complex, subunit C, integral membrane protein, 15kDa	20304	-0.6647133231163025	-0.0035936204	Yes
**row_108**	ATP5O	ATP5O	ATP synthase, H+ transporting, mitochondrial F1 complex, O subunit (oligomycin sensitivity conferring protein)	20367	-0.7001892328262329	0.002472308	Yes
**row_109**	UQCRB	UQCRB	ubiquinol-cytochrome c reductase binding protein	20423	-0.748714804649353	0.008879767	Yes

Supplementary table 6: Canonical KEGG pathways from GSEA analysis of PD versus HC from three independent studies (GSE7621, GSE20141, GSE49036)

Rank	Pathway	Size	ES	NES	p-value	FDR q-value	FWER p-value	Rank at max
**1**	**CYTOKINE CYTOKINE RECEPTOR INTERACTION**	245	0.20	3.55	0.000	0.000	0.000	12458
**2**	RIBOSOME	79	0.33	3.45	0.000	0.000	0.000	12226
**3**	SYSTEMIC LUPUS ERYTHEMATOSUS	107	0.27	3.20	0.000	0.000	0.000	11113
**4**	HEMATOPOIETIC CELL LINEAGE	83	0.31	3.20	0.000	0.000	0.000	8590
**5**	**JAK STAT SIGNALING PATHWAY**	151	0.21	3.13	0.000	0.000	0.000	11808
**6**	PATHWAYS IN CANCER	315	0.15	3.04	0.000	0.000	0.000	11704
**7**	LEISHMANIA INFECTION	62	0.32	2.96	0.000	0.000	0.000	4089
**8**	NATURAL KILLER CELL MEDIATED CYTOTOXICITY	129	0.20	2.70	0.000	0.000	0.003	9742
**9**	ALLOGRAFT REJECTION	34	0.38	2.68	0.000	0.000	0.003	9742
**10**	AUTOIMMUNE THYROID DISEASE	49	0.31	2.60	0.000	0.001	0.005	10834
**11**	APOPTOSIS	82	0.24	2.58	0.000	0.001	0.008	4046
**12**	PROSTATE CANCER	87	0.24	2.56	0.000	0.001	0.009	6541
**13**	**TOLL LIKE RECEPTOR SIGNALING PATHWAY**	98	0.22	2.50	0.000	0.001	0.014	4550
**14**	GRAFT VERSUS HOST DISEASE	37	0.34	2.46	0.002	0.001	0.015	9742
**15**	CELL ADHESION MOLECULES CAMS	125	0.18	2.38	0.000	0.002	0.034	10088
**16**	INTESTINAL IMMUNE NETWORK FOR IGA PRODUCTION	45	0.30	2.35	0.000	0.003	0.042	10335
**17**	LEUKOCYTE TRANSENDOTHELIAL MIGRATION	107	0.19	2.30	0.000	0.004	0.057	10939
**18**	ANTIGEN PROCESSING AND PRESENTATION	81	0.21	2.28	0.002	0.004	0.071	9603
**19**	CHRONIC MYELOID LEUKEMIA	72	0.23	2.26	0.000	0.005	0.080	6692
**20**	FOCAL ADHESION	188	0.14	2.23	0.000	0.005	0.098	11862

GSEA analysis was quantile normalized and summarized for each comparison using justPlier implementation of Plier algorithm in R. ES = enrichment score; NES = normalized enrichment score; NOM p-val = nominal p-value; FDR q-val = False Discovery Rate; FWER p-val = family-wise error rate; RANK AT MAX = position in the ranked list at which the maximum enrichment score occurred.

Supplementary table 7: Position of SVs and their heteroplasmy percentage in *Ifnb*$^{-/-}$ mice.

Type of SVs	Breakpoints	Size (nt)	Gene	Heteroplasmy%
**Deletions**	1901–1902	1	mt-Rnr2	25
	3814–3823	9	mt-Tq	40
	10858–11491	633	mt-Nd4	1.12
	10899–11786	887	mt-Nd4/mt-Th/mt-Ts2/ mt-Tl2/mt-Nd5	1.3
	11031–11871	840	mt-Nd4/mt-Th/mt-Ts2/ mt-Tl2/mt-Nd5	0.58
	11035–11841	806	mt-Nd4/mt-Th/mt-Ts2/ mt-Tl2/mt-Nd5	0.87
	11055–11847	792	mt-Nd4/mt-Th/mt-Ts2/ mt-Tl2/mt-Nd5	0.86
	11201–11971	770	mt-Nd4/mt-Th/mt-Ts2/ mt-Tl2/mt-Nd5	0.9
	11205–12089	884	mt-Nd4/mt-Th/mt-Ts2/ mt-Tl2/mt-Nd5	0.74
	11839–11842	3	mt-Nd5	1.16
	12576–12954	378	mt-Nd5	1
	12768–13092	324	mt-Nd5	1.35
	12847–13099	252	mt-Nd5	1
	12849–13101	252	mt-Nd5	0.8
	12910–13201	291	mt-Nd5	9.4
	12923–13051	128	mt-Nd5	1.48
	13046–13047	1	mt-Nd5	2
	5930-10395 (PC)	4465	mt-Co1…mt-Nd4	60
**Inversions**	10521–10922	405	mt-Nd4	23.4
	10752–11418	666	mt-Nd4	53.46
	10786–11206	420	mt-Nd4	1.71
	10819–11208	397	mt-Nd4	3
	10893–11291	398	mt-Nd4	2
	10921–11416	495	mt-Nd4	1.17
	10921–11437	516	mt-Nd4	1.75
	11036_11409	379	mt-Nd4	0.85
	11296–11856	560	mt-Nd4/mt-Th/mt-Ts2/ mt-Tl2/mt-Nd5	33.33
	12103–12445	342	mt-Nd5	0.9
	12106–12487	381	mt-Nd5	0.59
	12193–12397	204	mt-Nd5	0.77
	12193–12448	255	mt-Nd5	0.77
	12229–12567	338	mt-Nd5	0.54
	12305–12398	93	mt-Nd5	1.62
	12668–12849	181	mt-Nd5	10.7
	12727–12997	270	mt-Nd5	0.61
**Short insertions**	10785-10785	44	mt-Nd4	4.95
	11424-11424	44	mt-Nd4	1.61
	13046-13046	1	mt-Nd5	6
**Tandem duplications**	10705-11453	748	mt-Nd4	1.23

Supplementary table 8: Position of SNVs, their consequences, and heteroplasmy percentage detected in in *Ifnb*$^{-/-}$ mice.

Substitution	Consequence	Gene	Amino acid change	Codons	Heteroplasmy %
**m.3331A>C**	missense_variant	mt-Nd1	p.N194T	AAC/ACC	4.93
**m.3399G>C**	missense_variant	mt-Nd1	p.A217P	GCC/CCC	66.67
**m.4520C>T**	missense_variant	mt-Nd2	p.L201F	CTC/TTC	47.87
**m.6205T>A**	missense_variant	mt-Co1	p.F293Y	TTC/TAC	54.55
**m.6225G>T**	missense_variant	mt-Co1	p.D300Y	GAC/TAC	22.28
**m.12788A>C**	stop_lost	mt-Nd5	p.X349S	AGA/AGC	27.34
**m.4679C>G**	missense_variant	mt-Nd2	p.P256A	CCA/GCA	67.77
**m.6661A>G**	missense_variant	mt-Co1	p.D445G	GAT/GGT	50.72
**m.7125T>G**	missense_variant	mt-Co2	p.V38G	GTC/GGC	83.33
**m.7578C>A**	missense_variant	mt-Co2	p.P189Q	CCA/CAA	24.62
**m.7878C>G**	missense_variant	mt-Atp8	p.S38W	TCA/TGA	50.53
**m.8498T>A**	missense_variant	mt-Atp6	p.I191N	ATT/AAT	54.63
**m.11332T>G**	missense_variant	mt-Nd4	p.L389W	TTA/TGA	63.33
**m.3707G>C**	stop_lost	mt-Nd1	p.X319Y	TAG/TAC	52.56
**m.6014T>G**	missense_variant	mt-Co1	p.I229M	ATT/ATG	46.34

Supplementary table 9: Common variations in *Ifnb*$^{-/-}$ single dopaminergic neurons.

		Replicate 1			Replicate 2			Replicate 3			
**Substitution**	**Consequence**	sDA-1	sDA-2	sDA-3	sDA-4	sDA-5	sDA-6	sDA-7	sDA-8	sDA-9	sDA-10
m.15616A>G	Intergenic	-	_-	_-	-_	27.78%	_-	-_	-_	-	9.50%

Supplementary Table 10: GO pathways negatively dysregulated in sporadic PD

Name	Size	Es	Nes	Nom P-Val	Fdr Q-Val	Fwer P-Val
**Phagocytosis**	17	-0.65865344	-1.865.414	0.0020283975	0.5887762	0,26180556
**Cytosolic_Part**	23	-0.5894047	-17.946.182	0.003937008	0.6464161	0,44166667
**Nuclear_Membrane_Part**	39	-0.5211393	-17.519.393	0.004040404	0.67625695	0,56041667
**Ras_Gtpase_Binding**	23	-0.5742026	-17.298.789	0.006048387	0.62698627	0,60763889
**Damaged_Dna_Binding**	19	-0.6070259	-17.264.965	0.0061728396	0.51599807	0,6125
**Carbohydrate_Transport**	19	-0.58509773	-16.804.694	0.014553014	0.66561043	0,66736111
**Protein_Serine_Threonine_Phosphatase_Activity**	23	-0.55747867	-16.694.049	0.009469697	0.6276737	0,675
**Oxidoreductase_Activity_Acting_On_The_Aldehyde_Or_Oxo_Group_Of_Donors**	22	-0.55824864	-16.572.144	0.007905139	0.60850006	0,06805556
**Membrane_Organization_And_Biogenesis**	124	-0.38442543	-1.642.454	00.00	0.6109516	0,68680556
**Lysosome**	55	-0.4465573	-16.421.394	0.0062240665	0.55132973	0,06875
**Lytic_Vacuole**	55	-0.44655734	-1.619.767	0.00877193	0.60498303	0,69166667
**Nucleotide_Excision_Repair**	19	-0.5513221	-16.126.367	0.01927195	0.5862652	0,69166667
**Nuclear_Pore**	28	-0.50892353	-16.085.578	0.018	0.5561783	0,69166667
**Pore_Complex**	32	-0.48488656	-16.016.433	0.022044089	0.5455537	0,69236111
**Protein_Tyrosine_Phosphatase_Activity**	53	-0.4302897	-15.945.115	0.010615711	0.5360159	0,69375
**Receptor_Mediated_Endocytosis**	31	-0.4874128	-15.865.837	0.031936128	0.53668886	0,69375
**Organelle_Inner_Membrane**	72	-0.41009468	-15.858.959	0.0040816325	0.5081687	0,69375
**Peroxisome_Organization_And_Biogenesis**	15	-0.587189	-15.849.314	0.030241935	0.48316646	0,69375
**Basement_Membrane**	34	-0.47176352	-1.584.306	0.025948104	0.45967603	0,69375
**Lipoprotein_Binding**	17	-0.5637113	-15.824.773	0.031128405	0.44327217	0,69375

Supplementary Table 11: Genes in GO pathway of Damaged DNA binding in sporadic PD

PROBE	GENE SYMBOL	GENE_TITLE	RANK IN GENE LIST	RANK METRIC SCORE	RUNNING ES	CORE ENRICHMENT
**ALKBH2**	ALKBH 2	alkB, alkylation repair homolog 2 (E. coli)	5841	0.057695191353559494	-0.24626464	No
**POLQ**	POLQ	polymerase (DNA directed), theta	6237	0.05161980912089348	-0.23193778	No
**OGG1**	OGG1	8-oxoguanine DNA glycosylase	9358	0.0073499130085110664	-0.37871787	No
**APTX**	APTX	aprataxin	9548	0.005126686301082373	-0.38456997	No
**MPG**	MPG	N-methylpurine-DNA glycosylase	10247	-0.00287461350671947	-0.41660854	No
**POLH**	POLH	polymerase (DNA directed), eta	10834	-0.0097323814406991	-0.43875444	No
**ERCC1**	ERCC1	excision repair cross-complementing rodent repair deficiency, complementation group 1	11269	-0.015008884482085705	-0.4500913	No
**ALKBH3**	ALKBH 3	alkB, alkylation repair homolog 3 (E. coli)	11370	-0.016211574897170067	-0.44442353	No
**DDB1**	DDB1	damage-specific DNA binding protein 1, 127kDa	12003	-0.02409016527235508	-0.45948216	No
**FANCG**	FANCG	Fanconi anemia, complementation group G	13073	-0.03760887309908867	-0.4869909	No
**MSH2**	MSH2	mutS homolog 2, colon cancer, nonpolyposis type 1 (E. coli)	13811	-0.04724147170782089	-0.49211907	No
**MSH3**	MSH3	mutS homolog 3 (E. coli)	14083	-0.050981342792510986	-0.47218353	No
**MSH6**	MSH6	mutS homolog 6 (E. coli)	16860	-0.0985795110464096	-0.543024	Yes
**ERCC4**	ERCC4	excision repair cross-complementing rodent repair deficiency, complementation group 4	17500	-0.11334554105997086	-0.5004744	Yes
**FEN1**	FEN1	flap structure-specific endonuclease 1	17943	-0.12525609135627747	-0.4406228	Yes
**XPA**	XPA	xeroderma pigmentosum, complementation group A	18806	-0.1580028086900711	-0.37991193	Yes
**DDB2**	DDB2	damage-specific DNA binding protein 2, 48kDa	19683	-0.2136475294828415	-0.28375423	Yes
**TDG**	TDG	thymine-DNA glycosylase	19964	-0.24537022411823273	-0.13805053	Yes
**XPC**	XPC	xeroderma pigmentosum, complementation group C	20084	-0.26051655411720276	0.025307255	Yes

Supplementary table 12: KEGG pathways positively dysregulated in sporadic PDD

Name	Size	Es	Nes	Nom P-Val	Fdr Q-Val	Fwer P-Val
**Kegg_Cytokine_Cytokine_Receptor_Interaction**	245	0.22641903	40.743.566	00.00	00.00	00.00
**Kegg_Olfactory_Transduction**	114	0.24842387	31.019.006	00.00	00.00	00.00
**Kegg_Jak_Stat_Signaling_Pathway**	151	0.2013886	28.935.444	00.00	6,74e+02	0.002
**Kegg_Neuroactive_Ligand_Receptor_Interaction**	252	0.153708	28.746.104	00.00	5,06e+02	0.002
**Kegg_Intestinal_Immune_Network_For_Iga_Production**	45	0.34367222	27.512.014	00.00	0.0013277071	0.006
**Kegg_Hematopoietic_Cell_Lineage**	83	0.2577003	27.105.896	00.00	0.0011064226	0.006
**Kegg_Natural_Killer_Cell_Mediated_Cytotoxicity**	129	0.18345445	24.394.183	00.00	0.0037768607	0.025
**Kegg_Autoimmune_Thyroid_Disease**	49	0.2950065	2.404.819	00.00	0.0042137867	0.031
**Kegg_Systemic_Lupus_Erythematosus**	107	0.1909124	23.049.753	00.00	0.0077755423	0.063
**Kegg_Primary_Immunodeficiency**	35	0.31640798	2.298.848	00.00	0.007218002	0.065
**Kegg_Toll_Like_Receptor_Signaling_Pathway**	98	0.19364467	22.521.276	00.00	0.010030048	00.01
**Kegg_Vegf_Signaling_Pathway**	71	0.21941513	21.575.115	00.00	0.018321719	0,12916667
**Kegg_Leishmania_Infection**	62	0.2353862	21.431.851	00.00	0.01889759	0,14236111
**Kegg_Renin_Angiotensin_System**	16	0.4420289	21.169.133	0.0060728746	0.020564906	0,1625
**Kegg_Prostate_Cancer**	87	0.1943094	21.126.213	0.003937008	0.01981547	00.24
**Kegg_Cytosolic_Dna_Sensing_Pathway**	**51**	**0.23900488**	**20.225.606**	**0.0077669905**	**0.031307276**	**0,26180556**
**Kegg_Vascular_Smooth_Muscle_Contraction**	110	0.15773632	1.979.751	0.005725191	0.0369755	0,30347222
**Kegg_Melanoma**	71	0.20253429	1.931.158	0.0039138943	0.04484946	0,3625
**Kegg_Leukocyte_Transendothelial_Migration**	107	0.15709539	19.072.187	0.0100603625	0.04829179	0,3875
**Kegg_Ecm_Receptor_Interaction**	81	0.18228298	18.875.932	0.0019646366	0.050027963	0,41458333

Supplementary table 13: Genes in KEGG pathway of Cytosolic DNA Sensing in sporadic PDD

PROBE	GENE SYMBOL	GENE_TITLE	RANK IN GENE LIST	RANK METRIC SCORE	RUNNING ES	CORE ENRICH.
**IRF3**	IRF3	interferon regulatory factor 3	158	0.7471717000007629	0.01192115	Yes
**IFNA10**	IFNA10	interferon, alpha 10	341	0.6695659756660461	0.0226747	Yes
**IFNA16**	IFNA16	interferon, alpha 16	417	0.6529250144958496	0.038633797	Yes
**IFNA7**	IFNA7	interferon, alpha 7	500	0.6337449550628662	0.054252345	Yes
**IKBKE**	IKBKE	inhibitor of kappa light polypeptide gene enhancer in B-cells, kinase epsilon	789	0.5841962099075317	0.059848998	Yes
**IFNA5**	IFNA5	interferon, alpha 5	919	0.5644056797027588	0.073180996	Yes
**IFNB1**	IFNB1	interferon, beta 1, fibroblast	1088	0.5449801683425903	0.08461565	Yes
**IRF7**	IRF7	interferon regulatory factor 7	1290	0.5256357789039612	0.09444485	Yes
**CASP1**	CASP1	caspase 1, apoptosis-related cysteine peptidase (interleukin 1, beta, convertase)	1491	0.5080535411834717	0.1043227	Yes
**IFNA14**	IFNA14	interferon, alpha 14	1552	0.5037133097648621	0.12101155	Yes
**IFNA17**	IFNA17	interferon, alpha 17	1572	0.5025635361671448	0.13969503	Yes
**IFNA13**	IFNA13	interferon, alpha 13	1676	0.4937666952610016	0.15429194	Yes
**DDX58**	DDX58	DEAD (Asp-Glu-Ala-Asp) box polypeptide 58	1737	0.4896621108055115	0.17098078	Yes
**TREX1**	TREX1	three prime repair exonuclease 1	1744	0.48921793699264526	0.19029672	Yes
**IL1B**	IL1B	interleukin 1, beta	1811	0.48461082577705383	0.20669368	Yes
**IL18**	IL18	interleukin 18 (interferon-gamma-inducing factor)	2133	0.46447300910949707	0.21068488	Yes
**IFNA4**	IFNA4	interferon, alpha 4	2568	0.44055870175361633	0.20917864	Yes
**NFKBIB**	NFKBIB	nuclear factor of kappa light polypeptide gene enhancer in B-cells inhibitor, beta	2619	0.4380071461200714	0.22635399	Yes
**IFNA2**	IFNA2	interferon, alpha 2	2763	0.4314329922199249	0.23900488	Yes
**IL6**	IL6	interleukin 6 (interferon, beta 2)	3539	0.3957005739212036	0.220909	No
**IFNA21**	IFNA21	interferon, alpha 21	5210	0.32905668020248413	0.15927142	No
**POLR3 H**	POLR3H	polymerase (RNA) III (DNA directed) polypeptide H (22.9kD)	5475	0.31937581300735474	0.16603567	No
**AIM2**	AIM2	absent in melanoma 2	5852	0.30751100182533264	0.16735113	No
**RELA**	RELA	v-rel reticuloendotheliosis viral oncogene homolog A, nuclear factor of kappa light polypeptide gene	6501	0.28471267223358154	0.15543379	No

		enhancer in B-cells 3, p65 (avian)				
**IFNA8**	IFNA8	interferon, alpha 8	6971	0.2692958116531372	0.15222481	No
**POLR3D**	POLR3D	polymerase (RNA) III (DNA directed) polypeptide D, 44kDa	7156	0.26343846321105957	0.16288105	No
**ADAR**	ADAR	adenosine deaminase, RNA-specific	7755	0.2434481978416443	0.15339622	No
**IFNA1**	IFNA1	interferon, alpha 1	9275	0.18969926238059998	0.09910477	No
**NFKBIA**	NFKBIA	nuclear factor of kappa light polypeptide gene enhancer in B-cells inhibitor, alpha	10129	0.15881672501564026	0.0772142	No
**IKBKG**	IKBKG	inhibitor of kappa light polypeptide gene enhancer in B-cells, kinase gamma	10223	0.1553581804037094	0.09229759	No
**POLR3G**	POLR3G	polymerase (RNA) III (DNA directed) polypeptide G (32kD)	10799	0.13380225002765656	0.08393171	No
**CCL5**	CCL5	chemokine (C-C motif) ligand 5	10848	0.13150088489055634	0.10120435	No
**POLR1D**	POLR1D	polymerase (RNA) I polypeptide D, 16kDa	10895	0.12877798080444336	0.1185743	No
**IFNA6**	IFNA6	interferon, alpha 6	12427	0.0653807669878006	0.06369905	No
**IKBKB**	IKBKB	inhibitor of kappa light polypeptide gene enhancer in B-cells, kinase beta	12502	0.061775267124176025	0.079706796	No
**RIPK3**	RIPK3	receptor-interacting serine-threonine kinase 3	12736	0.051115427166223526	0.0879792	No
**CCL4**	CCL4	chemokine (C-C motif) ligand 4	12828	0.0475873127579689	0.1031599	No
**ZBP1**	ZBP1	Z-DNA binding protein 1	13954	-0.0033709052950143814	0.068036534	No
**CXCL10**	CXCL10	chemokine (C-X-C motif) ligand 10	14349	-0.025276580825448036	0.06847629	No
**PYCARD**	PYCARD	PYD and CARD domain containing	14456	-0.030308295041322708	0.082927234	No
**POLR3K**	POLR3K	polymerase (RNA) III (DNA directed) polypeptide K, 12.3 kDa	15209	-0.07029853761196136	0.06595031	No
**CHUK**	CHUK	conserved helix-loop-helix ubiquitous kinase	15348	-0.0784481018781662	0.07884446	No
**POLR1C**	POLR1C	polymerase (RNA) I polypeptide C, 30kDa	15589	-0.09314949810504913	0.08677631	No
**RIPK1**	RIPK1	receptor (TNFRSF)-interacting serine-threonine kinase 1	16255	-0.13543589413166046	0.07403193	No
**POLR3GL**	POLR3GL	polymerase (RNA) III (DNA directed) polypeptide G (32kD) like	16635	-0.16024237871170044	0.07520144	No
**TBK1**	TBK1	TANK-binding kinase 1	17057	-0.18947164714336395	0.07432765	No

**NFKB1**	NFKB1	nuclear factor of kappa light polypeptide gene enhancer in B-cells 1 (p105)	18278	-0.2935390770435333	0.034582533	No
**POLR3 C**	POLR3C	polymerase (RNA) III (DNA directed) polypeptide C (62kD)	19188	-0.39815741777420044	0.009967562	No
**POLR3 A**	POLR3A	polymerase (RNA) III (DNA directed) polypeptide A, 155kDa	19447	-0.43613821268081665	0.017023716	No
**POLR3 B**	POLR3B	polymerase (RNA) III (DNA directed) polypeptide B	20028	-0.5650253891944885	0.008414581	No
**POLR3 F**	POLR3F	polymerase (RNA) III (DNA directed) polypeptide F, 39 kDa	20365	-0.6991318464279175	0.011676038	No

Supplementary table 14: Genes in KEGG pathway of Toll-Like Receptor in sporadic PDD

PROBE	GENE SYMBOL	GENE_TITLE	RANK IN GENE LIST	RANK METRIC SCORE	RUNNING ES	CORE ENRICHMENT
**PIK3R2**	PIK3R2	phosphoinositide-3-kinase, regulatory subunit 2 (p85 beta)	89	0.8091794848442078	0.0058643115	Yes
**AKT1**	AKT1	v-akt murine thymoma viral oncogene homolog 1	102	0.7975646257400513	0.0154832555	Yes
**IRF3**	IRF3	interferon regulatory factor 3	158	0.7471717000007629	0.023005456	Yes
**IRF5**	IRF5	interferon regulatory factor 5	254	0.697068452835083	0.0285772	Yes
**IFNA10**	IFNA10	interferon, alpha 10	341	0.6695659756660461	0.034587797	Yes
**IFNA16**	IFNA16	interferon, alpha 16	417	0.6529250144958496	0.041134767	Yes
**IRAK4**	IRAK4	interleukin-1 receptor-associated kinase 4	443	0.6466980576515198	0.050119814	Yes
**IFNA7**	IFNA7	interferon, alpha 7	500	0.6337449550628662	0.057593253	Yes
**IRAK1**	IRAK1	interleukin-1 receptor-associated kinase 1	570	0.6173790097236633	0.06443279	Yes
**TLR5**	TLR5	toll-like receptor 5	773	0.586562991142273	0.06478706	Yes
**IKBKE**	IKBKE	inhibitor of kappa light polypeptide gene enhancer in B-cells, kinase epsilon	789	0.5841962099075317	0.07425972	Yes
**TLR9**	TLR9	toll-like receptor 9	826	0.5783514976501465	0.08270839	Yes
**IFNA5**	IFNA5	interferon, alpha 5	919	0.5644056797027588	0.08842641	Yes
**MAPK12**	MAPK12	mitogen-activated protein kinase 12	933	0.5621751546859741	0.0979966	Yes
**IFNB1**	IFNB1	interferon, beta 1, fibroblast	1088	0.5449801683425903	0.100691415	Yes
**MYD88**	MYD88	myeloid differentiation primary response gene (88)	1091	0.5445457100868225	0.11079797	Yes
**IRF7**	IRF7	interferon regulatory factor 7	1290	0.5256357789039612	0.11134729	Yes
**IFNA14**	IFNA14	interferon, alpha 14	1552	0.5037133097648621	0.108824626	Yes
**IFNA17**	IFNA17	interferon, alpha 17	1572	0.5025635361671448	0.11810224	Yes
**MAPK3**	MAPK3	mitogen-activated protein kinase 3	1589	0.5009751915931702	0.12752613	Yes
**IFNA13**	IFNA13	interferon, alpha 13	1676	0.4937666952610016	0.13353673	Yes
**MAP2K7**	MAP2K7	mitogen-activated protein kinase kinase 7	1789	0.48538392782211304	0.13827953	Yes
**IL1B**	IL1B	interleukin 1, beta	1811	0.48461082577705383	0.14745963	Yes
**TICAM2**	TICAM2	toll-like receptor adaptor molecule 2	1948	0.4752063751220703	0.15103215	Yes
**TIRAP**	TIRAP	toll-interleukin 1 receptor (TIR) domain containing adaptor protein	2458	0.4467725455760956	0.13641664	Yes
**AKT2**	AKT2	v-akt murine thymoma viral oncogene homolog 2	2505	0.44389283657073975	0.1443777	Yes
**IFNA4**	IFNA4	interferon, alpha 4	2568	0.44055870175361633	0.15155856	Yes
**IFNA2**	IFNA2	interferon, alpha 2	2763	0.4314329922199249	0.15230292	Yes
**PIK3CG**	PIK3CG	phosphoinositide-3-kinase, catalytic, gamma polypeptide	2836	0.42768803238868713	0.15899618	Yes

**TICAM1**	TICAM1	toll-like receptor adaptor molecule 1	3214	0.40976935625076294	0.1508172	Yes
**IL12B**	IL12B	interleukin 12B (natural killer cell stimulatory factor 2, cytotoxic lymphocyte maturation factor 2, p40)	3327	0.40452226996421814	0.15555999	Yes
**IL6**	IL6	interleukin 6 (interferon, beta 2)	3539	0.3957005739212036	0.15547541	Yes
**MAP2K2**	MAP2K2	mitogen-activated protein kinase kinase 2	3553	0.39521417021751404	0.16504559	Yes
**TLR8**	TLR8	toll-like receptor 8	3674	0.39029771089553833	0.1693983	Yes
**CD80**	CD80	CD80 molecule	4012	0.3756698966026306	0.16316976	Yes
**CD86**	CD86	CD86 molecule	4456	0.3579317033290863	0.15177251	Yes
**CD40**	CD40	CD40 molecule, TNF receptor superfamily member 5	4505	0.3564003109931946	0.15963605	Yes
**PIK3CD**	PIK3CD	phosphoinositide-3-kinase, catalytic, delta polypeptide	4829	0.3439567983150482	0.15409018	Yes
**TNF**	TNF	tumor necrosis factor (TNF superfamily, member 2)	4987	0.3369358777999878	0.15663871	Yes
**TLR4**	TLR4	toll-like receptor 4	5206	0.32920098304748535	0.15621279	Yes
**IFNA21**	IFNA21	interferon, alpha 21	5210	0.32905668020248413	0.1662706	Yes
**IL8**	IL8	interleukin 8	5231	0.32841554284095764	0.17549944	Yes
**TLR1**	TLR1	toll-like receptor 1	5232	0.3283589482307434	0.18570353	Yes
**CTSK**	CTSK	cathepsin K (pycnodysostosis)	5629	0.31447649002075195	0.17659807	Yes
**MAPK13**	MAPK13	mitogen-activated protein kinase 13	5826	0.30847060680389404	0.1772449	Yes
**CASP8**	CASP8	caspase 8, apoptosis-related cysteine peptidase	6043	0.3010207712650299	0.17691651	Yes
**FOS**	FOS	v-fos FBJ murine osteosarcoma viral oncogene homolog	6061	0.30031004548072815	0.18629165	Yes
**TLR2**	TLR2	toll-like receptor 2	6467	0.28594449162483215	0.17674734	Yes
**RELA**	RELA	v-rel reticuloendotheliosis viral oncogene homolog A, nuclear factor of kappa light polypeptide gene enhancer in B-cells 3, p65 (avian)	6501	0.28471267223358154	0.1853423	Yes
**IL12A**	IL12A	interleukin 12A (natural killer cell stimulatory factor 1, cytotoxic lymphocyte maturation factor 1, p35)	6541	0.2833622097969055	0.19364467	Yes
**IFNA8**	IFNA8	interferon, alpha 8	6971	0.2692958116531372	0.1829301	No
**PIK3R5**	PIK3R5	phosphoinositide-3-kinase, regulatory subunit 5, p101	7080	0.26564356684684753	0.18786794	No
**SPP1**	SPP1	secreted phosphoprotein 1 (osteopontin, bone sialoprotein I, early T-lymphocyte activation 1)	8399	0.22000934183597565	0.13380441	No
**IFNAR1**	IFNAR1	interferon (alpha, beta and omega) receptor 1	8944	0.2016969919204712	0.11748226	No
**FADD**	FADD	Fas (TNFRSF6)-associated via death domain	9268	0.18991141021251678	0.11193639	No

**IFNA1**	IFNA1	interferon, alpha 1	9275	0.18969926238059998	0.1218479	No
**CXCL11**	CXCL11	chemokine (C-X-C motif) ligand 11	9623	0.1763661503791809	0.11513176	No
**LBP**	LBP	lipopolysaccharide binding protein	9679	0.1745533049106598	0.122653954	No
**PIK3R3**	PIK3R3	phosphoinositide-3-kinase, regulatory subunit 3 (p55, gamma)	9820	0.16914792358875275	0.12603143	No
**NFKBIA**	NFKBIA	nuclear factor of kappa light polypeptide gene enhancer in B-cells inhibitor, alpha	10129	0.15881672501564026	0.12121698	No
**IKBKG**	IKBKG	inhibitor of kappa light polypeptide gene enhancer in B-cells, kinase gamma	10223	0.1553581804037094	0.12688625	No
**TLR7**	TLR7	toll-like receptor 7	10260	0.154314324259758	0.13533492	No
**PIK3CA**	PIK3CA	phosphoinositide-3-kinase, catalytic, alpha polypeptide	10647	0.139031782746315	0.12671708	No
**CCL5**	CCL5	chemokine (C-C motif) ligand 5	10848	0.13150088489055634	0.12716886	No
**TOLLIP**	TOLLIP	toll interacting protein	11206	0.1171623021364212	0.119965106	No
**TLR3**	TLR3	toll-like receptor 3	12402	0.06631596386432648	0.07189924	No
**IFNA6**	IFNA6	interferon, alpha 6	12427	0.0653807669878006	0.08093305	No
**IKBKB**	IKBKB	inhibitor of kappa light polypeptide gene enhancer in B-cells, kinase beta	12502	0.061775267124176025	0.08752879	No
**MAPK11**	MAPK11	mitogen-activated protein kinase 11	12513	0.061489593237638474	0.09724525	No
**CXCL9**	CXCL9	chemokine (C-X-C motif) ligand 9	12521	0.061244554817676544	0.107108004	No
**CCL4**	CCL4	chemokine (C-C motif) ligand 4	12828	0.0475873127579689	0.10239108	No
**LY96**	LY96	lymphocyte antigen 96	12882	0.04519515112042427	0.1100108	No
**MAP2K4**	MAP2K4	mitogen-activated protein kinase kinase 4	13732	0.006487403530627489	0.07881641	No
**RAC1**	RAC1	ras-related C3 botulinum toxin substrate 1 (rho family, small GTP binding protein Rac1)	13957	-0.003696293104439974	0.07809792	No
**CD14**	CD14	CD14 molecule	14087	-0.010682184249162674	0.082011774	No
**MAPK8**	MAPK8	mitogen-activated protein kinase 8	14294	-0.022509494796395302	0.08217099	No
**CXCL10**	CXCL10	chemokine (C-X-C motif) ligand 10	14349	-0.025276580825448036	0.08974195	No
**TLR6**	TLR6	toll-like receptor 6	14389	-0.02677215449512005	0.098044336	No
**MAPK1**	MAPK1	mitogen-activated protein kinase 1	14515	-0.03341846913099289	0.102153234	No
**MAP3K8**	MAP3K8	mitogen-activated protein kinase kinase kinase 8	14811	-0.04813077300786972	0.09797269	No
**MAP3K7**	MAP3K7	mitogen-activated protein kinase kinase kinase 7	14865	-0.050766076892614365	0.105592415	No
**MAPK14**	MAPK14	mitogen-activated protein kinase 14	15064	-0.062105000019073486	0.10614172	No
**MAPK10**	MAPK10	mitogen-activated protein kinase 10	15173	-0.0674557238817215	0.111079566	No
**CHUK**	CHUK	conserved helix-loop-helix ubiquitous kinase	15348	-0.0784481018781662	0.11279915	No

**PIK3CB**	PIK3CB	phosphoinositide-3-kinase, catalytic, beta polypeptide	15426	-0.08269017934799194	0.119248606	No
**JUN**	JUN	jun oncogene	15478	-0.0859072357416153	0.12696585	No
**MAP2K6**	MAP2K6	mitogen-activated protein kinase kinase 6	16027	-0.12146072089672089	0.11044865	No
**RIPK1**	RIPK1	receptor (TNFRSF)-interacting serine-threonine kinase 1	16255	-0.13543589413166046	0.109583884	No
**IFNAR2**	IFNAR2	interferon (alpha, beta and omega) receptor 2	16987	-0.1839001476764679	0.08414334	No
**TBK1**	TBK1	TANK-binding kinase 1	17057	-0.18947164714336395	0.09098288	No
**MAP2K1**	MAP2K1	mitogen-activated protein kinase kinase 1	17222	-0.20127750933170319	0.09319008	No
**TRAF3**	TRAF3	TNF receptor-associated factor 3	17639	-0.2368105947971344	0.08310939	No
**AKT3**	AKT3	v-akt murine thymoma viral oncogene homolog 3 (protein kinase B, gamma)	17705	-0.24429956078529358	0.09014398	No
**STAT1**	STAT1	signal transducer and activator of transcription 1, 91kDa	17857	-0.25760892033576965	0.092985086	No
**NFKB1**	NFKB1	nuclear factor of kappa light polypeptide gene enhancer in B-cells 1 (p105)	18278	-0.2935390770435333	0.08270935	No
**MAPK9**	MAPK9	mitogen-activated protein kinase 9	19766	-0.49379464983940125	0.020405142	No
**TRAF6**	TRAF6	TNF receptor-associated factor 6	19942	-0.5384869575500488	0.022075968	No
**PIK3R1**	PIK3R1	phosphoinositide-3-kinase, regulatory subunit 1 (p85 alpha)	20070	-0.5771433115005493	0.026087344	No

Mitochondrial DNA damage triggers spread of Parkinson's disease like pathology

Emilie Tresse[1], Joana Marturia-Navarro[1], Wei Qi Guinevere Sew[1], Marina Cisquella-Serra[1], Elham Jaberi[1], Lluis Riera-Ponsati[1], Natasha Fauerby[1], Erling Hu[1], Oliver Kretz[2], Susana Aznar[3] and Shohreh Issazadeh-Navikas[1*]

[1]Neuroinflammation Unit, Biotech Research & Innovation Centre (BRIC), Faculty of Health and Medical Sciences, University of Copenhagen, Copenhagen Biocentre, Ole Maaløes Vej 5, DK-2200 Copenhagen N, Denmark

[2]Department of Medicine, University Medical Center Hamburg-Eppendorf, Hamburg, Germany

[3]Centre for Neuroscience and Stereology, University Hospital Bispebjerg-Frederiksberg, 2400 Copenhagen, Denmark.

[*]**Corresponding author:** Shohreh Issazadeh-Navikas, Professor, Neuroinflammation Unit, Biotech Research and Innovation Centre (BRIC), University of Copenhagen, Ole Maaløes Vej 5, DK-2200 Copenhagen N, Denmark

E-mail: shohreh.issazadeh@bric.ku.dk
Tel: +45-353 25649

Running Title: ***Mitochondrial DNA damage propagates PD***

Supplementary Materials and Methods

Brain samples

Human brain samples from non-neurological affected healthy controls (NCs) ($N = 7$, 3 females and 4 males) and sporadic PD (N=7, 3 females, 4 males) were dissected from the medial frontal gyrus, a region implicated in cognitive impairments in PD[1,2]. Samples were acquired from the Harvard Brain Tissue Resource Center (Harvard Medical School, USA), Bispebjerg Hospital Brain Bank (Copenhagen, Denmark), and Netherlands Brain Bank (the Netherlands) [ethics (DNK) approval, jr. no. H-20066711]. All diagnoses were confirmed by postmortem pathology.

Human microarray data and processing

For Affymetrix microarray analysis, publicly available microarray data from Parkinson disease dementia (PDD), Parkinson disease without dementia (PD), and healthy controls (HC) were obtained (GSE7621, GSE20141, GSE49036) [PMID:18649390][3], preprocessing and normalization were performed with the frma package using the “robust weighted average" option for probe summarization as we previously reported[4].

Mice

Ifnb$^{-/-}$ or *Ifnar*$^{-/-}$ mice were backcrossed for 20 generations to C57BL6 mice and utilized as models for PD[4,5]. The C57BL6 was used as WT animals. Mice were housed in standard facilities. Sex- (equal proportion male and female), age (10 weeks old at injection), and weight-matched (28 to 31g). All experiments followed the Danish ethical standards under ethical permission license 2018-15-0201-01572. All injections and behavioral tests were performed in a randomized double-blind manner.

mtDNA preparation for *in vitro* experiments

mtDNA was prepared from WT, *Ifnb*$^{-/-}$ or *Ifnar1*$^{-/-}$ cortical neurons purified mitochondria, obtained from fractionation as described earlier[6]. Nuclear fraction was conserved to purify nuclear DNA (nuDNA). DNA was purified from fractions using the DNA blood and tissue kit (Qiagen, cat nº 69504). Quality of mtDNA was verified by qPCR prior to use.

Primary neuron, MEFs and Neuro2A (N2A) neuroblastoma cell line preparation

Primary cortical neuron (CN) cultures were obtained from the microdissected cortices from 0 to 1-day-old B10.RIII *Ifnb*$^{+/+}$, B10.RIII *Ifnb*$^{-/-}$ or C57BL6 *Ifnar*$^{-/-}$ mice and kept in Hibernate A medium w/o Calcium (BrainBits LLC, HA-Ca) supplemented with B27 (Gibco by Life Technologies, 17504-044) and $CaCl_2$ during dissection. Papain (2mg/mL) was used to dissociate the tissue for 10 minutes at 37ºC, and a single cell suspension was obtained by pipetting the cortices 10-13 times with a glass

pipette. Then, cells were centrifuged at x300g for 10 minutes and were resuspended in Neurobasal A medium (Gibco by Life Technologies, 10888-022) supplemented with B-27 (Gibco by Life Technologies, 17504-044), GlutaMAX (Gibco by Life Technologies, 35050-038) and 10ug/ml Gentamicin (Gibco by Life Technologies, 15710- 049). Primary CNs were seeded in cell culture dishes coated with poly-D-lysine hydrobromide (Sigma-Aldrich, P7280-5MG) at a density of $0,5x10^6$ cells/ml. Cells were cultured in Buffer B at 37ºC with 5% CO_2 and half media was changed every 3-4 days.

Neurons are fully differentiated by DIV6 and then gradually develop neurodegenerative-like cell-death starting around DIV15[7].

Immortalized Mouse Embryonic Fibroblasts (MEFs) were generated as described earlier [6].

The NTC and *Ifnb*$^{-/-}$ Neuro2A (N2A) neuroblastoma cell line were generated by CRISPR/Cas9 editing as previously described[6].

MEFs and N2A were cultured in DMEM containing GlutaMax (ThermoFisher cat num. 10569010) supplemented with 10% FBS.

ELISA for oxidative mitochondrial DNA release (8-OHdG)

Supernatants from N2A cells and primary cortical neurons were centrifuged, passed through a 0.22-µm filter and stored at -80°C. Oxidative mitochondrial DNA release was quantified using the DNA Damage (8-OHdG) ELISA kit (Bosterbio #EK7008) per the manufacturer's instructions.

Extracellular Vesicles (EVs) purification

EVs were purified from cell culture medium, using the ExoEasy kit (Qiagen) following manufacturer recommendations. EVs were then pelleted by ultracentrifugation at 160000g for 2h at 4ºC and resuspended in culture medium for EV treatment or in lysis buffer for protein analysis or in PBS for DNA purification.

From brain tissue, EVs were purified from fresh collected tissue as described earlier[8].

***In vitro* treatments**

Neuro2A and MEFs cells were cultured as previously described[6]. To induce oxidative mtDNA damages, neurons were treated for 30min with 50uM H2O2 and then let recover in full medium for 24h to ensure nuDNA repair[9]. Mitotempo was used at a concentration 2mM of and S3qel at a concentration of 10mM.

MtDNA treatment: N2As or CNs were treated for 24h with 30ng/mL of mtDNA purified as previously described and quantified with NanoDrop.

TLR inhibitors:

TLR9 inhibitor ODN-2088 (InvivoGen #tlrl-2088) was always used at a final concentration of 2.5 μM (1:400).

TLR4 inhibitor, LPS-rs (InvivoGen #tlrl-prslps) from the photosynthetic bacterium *Rhodobacter sphaeroides* was always used at a final concentration of 0.5 ng/mL (1:2000).

cGAS inhibitor RU.521 (InvivoGen #inh-ru521) was always used at a final concentration of 0.5 ng/mL (1:4000). (Please see Supplementary table 1).

Knock down: Reverse transfection was carried out on N2A cells by preparing a master mix of 25nM siRps3 (Dharmacon L-047921-01-0005) or 25μM siTLR4 (Dharmacon L-047487-00- 0005) and its respective non-targeting control (siNTC) diluted in 1X siRNA buffer (Dharmacon B-002000-UB-100) together with Optimem buffer (ThermoFisher 31985070) and Lipofectamine RNAiMAX (Invitrogen 13778030). The master mix was incubated 20 minutes at room temperature and then added to a 6- or 96-well plate. N2A cells were then added on top of the solution and left on the incubator at 37C. Efficiency of knock-down was assessed 1-day afterwards for siRps3 knock-down and 3-days afterwards for siTLR4 knock-down.

On the other hand, cortical neurons were seeded in 12-well plate at a density of $0,8 \cdot 10^6$ cells and transfection was carried out (DIV=2) by preparing a master mix of 100nM siRps3 or 100nM siTLR4 and its respective siNTC diluted in 1X siRNA buffer together with Neurobasal A buffer and Lipofectamine RNAiMAX. The master mix was incubated 20 minutes at room temperature and then added on the cells. Efficiency of knock-down was assessed 3-days afterwards.

Cellular assays

Live cell staining for cell death

Annexin V Dye (Sartorius #4642) was used to monitor cell death in real time. Primary cortical neurons were seeded in a 96-well plate (Thermo #167008) and Annexin V was added together with the treatment (1:200) at D7. Images were acquired with an IncuCyte S3 Basic Analyzer (Essen Bioscience) at 10 or 20x every 4 or 8h for 2 days. Confluence was analyzed with IncuCyte S3 Base Software.

DCFDA

DCFDA is a cell-permeant form of fluorescein that has been chemically reduced and is commonly employed as an indicator for reactive oxygen species (ROS) within cells. When the acetate groups are cleaved by intracellular esterases and undergo oxidation, the initially nonfluorescent H2DCFDA is converted into the highly fluorescent 2',7'-dichlorofluorescein (DCF). This conversion indicates the presence of oxidative activity, which can be attributed to hydrogen peroxide, peroxynitrite, or hydroxyl radicals. Additionally, although to a lesser extent, superoxide anions can also contribute to the oxidation of DCFDA. The fluorescence level of DCF has been extensively used as means to assess oxidation through techniques such as fluorescence-activated cell sorting (FACS) and microscopy in various cell types, including neurons[10-12]. Accordingly, we employed DCFDA to measure the extent of oxidation at a specific time point in our study comparing different stimuli and /or inhibitors as described earlier[6]. Briefly, CNs with a specific treatment were added DCFDA (5uM), incubated for

30 min at 37C, washed with warm media and incubated again. After 15 minutes, cells were imaged in the Incucyte® S3 Live-Cell Analysis Instrument.

MitoSox

MitoSOX™ Red Mitochondrial Superoxide Indicator (#M36008) was used to selectively detect superoxide in the mitochondria of live cells (CNs or N2A) Shortly, MitoSOX was used at a final concentration of 5μM and quantified using an Incucyte S3 (20X), as described previously[6], thresholding over the background signal including the one obtained from the nuclei.

Cell metabolic activity assay (MTT)

The Cell Proliferation Kit I (MTT) (Sigma Aldrich #11465007001) was used to measure cell metabolic activity. Primary cortical neurons or N2A cells were seeded in a 96-well plate and MTT labelling reagent was added at DIV6/9 or 72h after siRNA transduction respectively. After 4h incubation, the solubilization buffer was added and left overnight at 37C. Absorbance was measured at 550-600 nm in a GloMax. Discover Microplate Reader (Promega). Absorbance was normalized to confluency which was analyzed by IncuCyte S3 Base Software.

Cell fractionation

Cell fractionation was performed as described previously[6]. Briefly, cells were mechanically lysed with a 27G syringe in MTiso Buffer and were subsequently centrifuged at different speeds to separate nuclear, mitochondrial and cytoplasmic fractions.

Immunostaining, microscopy and analysis

Immunostainings and imaging were performed as described previously[13,6]. Antibodies reference and concentration are listed in Supplementary Table 2.

Cells were visualized by confocal microscopy (Leica TCS Sp8 X). Pictures were taken at 2,048 x 2,048 resolution, bidirectional acquisition, pinhole <1.0 and frame average = 4. Settings were applied equally across all fluorescent images from an experiment. Signal and colocalisation were quantified using FIJI (Scripts in below after the Supplementary material and method).

Microscope from GE Healthcare in Cell Analyzer 2200 was used for Caspase 3 staining. Images were taken in Incell 2200 Analyzer software. Plate Nunc 160376 96-well plate was used to set up imaging. Protocol was set up using Fluorescence with Nikon 20X/0.45, Plan Fluor, ELWD, Corr Collar 0-2.0, CFI/60.

Incucyte® Annexin V Dye (Sartorius #4642) was used to monitor cell death in real time.

Primary cortical neurons were seeded in a 96-well plate (Thermo #167008) and Annexin V was added together with the treatment (1:200) at D7. Images were acquired with an Incucyte S3 Basic Analyzer (Essen Bioscience) at 10 or 20x every 4 or 8h for 2 days. Confluence was analyzed with IncuCyte® S3 Base Software.

Electron microscopy

3 months post mtDNA or PBS injection, mice were intracardially perfused with 2% PFA (EM-grade, Electron Microscopy Sciences, cat. # 15710) and 2% glutaraldehyde (Sigma, cat. # 340855) in 0.1 M PB, pH 7.4. Brains were removed and shipped with unexpected delays to Germany. Coronal sections of striatum and frontal cortical regions were cut on a vibratome (50 µm). Sections were contrasted using 0.5% OsO_4 and 1% uranylacetate, dehydrated, and embedded in epoxy resin (Durcupan, Sigma-Aldrich, cat. # 44611). Sections, approximately 70 nm thick, were cut with a Ultracut 7 (Leica, Wienna, Austria) and collected on copper grids with Formvar supporting membranes. Sections were examined with a Philips CM 100 Transmission EM (Philips, Eindhoven, The Netherlands), operated at an accelerating voltage of 80 kV. Digital images were recorded with an OSIS Veleta digital slow scan 2k x 2k CCD camera and the ITEM software package.

qPCR

qPCR was performed as described earlier[6] using primers in Supplementary table 3.

qPCR from SN were prepared from 1 to 4 SN filtered a 0.2micron gauge filter. DNA was purified using the Blood and Tissue DNA kit (Qiagen), then quantified using a nanodrop and qPCR was performed as previously described.

Western Blotting

Tissue: Tissue was shredded using a qiashredder (Qiagen) and process using the All-prep Protein-RNA-DNA kit (Qiagen). Protein pellet was resuspended in a final 4M Urea. 2X loading buffer was added and sample boiled for 10 minutes before electrophoresis.

Cells: As described earlier[6]. In detail: Cells were washed in PBS and lysed with RIPA lysis buffer for 10 minutes on ice. Then, cells were scrapped, collected, and sonicated using a Bioruptor (Diagenode #B01020001), 8x30 second cycles. Samples were centrifuged at 13200g for 5 minutes and supernatant was used for loading.

Cell lysate samples were added 6x Laemli (300 mM Tris-HCl, 12% (w/v) SDS, 60%(v/v) Glycerol and Bromophenyl blue) with β-Mercaptoethanol (0.85 M) at appropriate volume. Proteins were denatured at 95 °C for 5 min and equal amounts of protein were loaded (10-20 μg) and separated on NuPAGE 4-12 % precast gels (Life Technologies NP0335BOX) in MES buffer or NuPAGE 4-20% Tris-Glycine gels (Invitrogen XP04205BOX) in Tris-Glycine buffer.

PageRuler Plus Prestain Protein Ladder (Thermo Scientific #815-968-0747) was used to indicate protein size. Proteins were subsequently transferred to Nitrocellulose membranes and blocked in TBST 1x (Thermo #J77500-K8) with skim milk or BSA (5%) for 1 h at room temperature. Membranes were incubated with primary antibodies diluted at appropriate concentration in 5% BSA and 0.05% Sodium Azide in TBST or in Antibody diluent solution (Thermo #003118) overnight at 4ºC. Then, membranes were washed in TBST x3 times for 5 minutes and incubated with horseradish

peroxidase (HRP) or fluorophore coupled secondary antibodies in washing buffer with skim milk or BSA (5%) for 1 h at room temperature.

Membranes were developed using chemiluminescent (ECL) HRP detection substrate (Millipore) or SuperSignal West Femto Maximum Sensitivity Substrate (Thermo Scientific #34096) for low expression proteins. Odyssey system (Li-Cor) was used for detection of fluorescence signal. As a general rule, Fiji software was used for quantification of Western blots. ImageJ software was used to analyse only Western blots performed with secondary antibodies coupled to fluorophore and images taken with Li-Cor Odyssey CLx Imaging system provided that the signal was very clear.

Gels were stained with ProBlue Safe Stain (Giotto #G00PB001) and subsequently washed in dH2O.

Mass Spectrometry and Proteomic analysis

Sample preparation

EVs were purified from media of 3 WT and 3 *Ifnb*$^{-/-}$ primary cortical neuronal cultures as previously described and final pellet was resuspended in 50 uL of Guanidine buffer containing 6M Guanidinium Hydrochloride, 10 mM Tris (2-carboxyethylphosphine), 40 mM 2-Chloroacetamide and 50 mM HEPES. Samples were kept at -80ºC until they were sent to our collaborators for LC MS analysis at DTU Proteomics facility. Peptides were digested with lysyl endopeptidase C and trypsin. Enzyme activity was quenched by adding 2% trifluoroacetic acid to a final concentration of 1%. The peptide samples were desalted using Sep-Pak column. Peptides were eluted over 140min gradient (4% to 23% in 76min, 23% to 38% in 30min, 38% to 60% in 3min, 60% to 95% in 4min, 95% for 6min, 4% for 21min using 95% buffer B 80% Acn, 0.1% FA) at 250 nl/min from 50cm uPAC column and run on Thermo Fisher Fusion instrument using Top Speed method. Thermo Fisher Fusion instrument MS1 was set to 120.000 resolution, scanning between 400-1500 m/z range, max IT of 50ms, AGC 4e5; ddMS2 scans were triggered after recognition of ions between 2&7 charge, dynamically excluded for 60sec. MS2 cycle was set to scan all selected ions within 3 sec, isolated at 1.6m/z, using rapid Ion

Trap scans, with 35ms injection time, HCD set to 30, AGC target 1e5. Data were automatically analyzed by Proteome Discoverer v2.4.

Differential expression proteomics analysis

DEP package (Bioconductor) was used for data preparation and differential expression analysis of proteomics data in RStudio (version 1.4.1106 with R version 4.0.4). Missing values were imputed with a pseudocount equivalent to the minimum abundance detected divided by 2 (= 1077). Differential protein expression analysis between *Ifnb*$^{+/+}$ and *Ifnb*$^{-/-}$ EVs was analyzed using limma and significant cut-offs were defined as a< 0.05 and Fold change > 1.5. Visualization of the data was either generated by DEP functions in R, ggplot2 (v3.3.5), heatmapper.ca or Proteome Discoverer (v2.4) (R script after the Supplementary methods). Gene Set Enrichment Analysis (GSEA v4.1.0) was performed in the data set previously prepared. As no TLR activator/modulator gene list existed and we observed some of these proteins amongst the most modified genes, we created one based on literature (Supplementary table 1).

mtDNA/Rps3 immunoprecipitation

Neuro2A cells were cultured in 15cm plates. Upon reaching 80% confluency, medium was removed, and cells were cross-linked in 1% PFA in DMEM 10% FBS for 10 min at RT with shaking. Crosslinking reaction was stopped by addition of 0,125M glycine for 5-10 mins at RT with shaking. Cells were then washed two times with ice-cold PBS and a third time with PBS containing Protease and phosphatase inhibitors. From this point, cells were kept on ice.

Cells were then collected and lysed in RIPA buffer. Protein concentration in lysates was quantified and 0,5 mg was used for each Immunoprecipitation with 30μL of pre-cleared Protein G Sepharose 4 Fast Flow beads and 6μL anti-Rps3 or anti-Tfam antibody. After incubation ON at 4ºC, beads were first washed with chilled 500μL lysis buffer, then with 500μL high-salt wash buffer (250mM LiCL, 1% NP-40, 1% sodium deoxycholate, 1mM EDTA, 10mM tris-HCL (pH=8)) and then with 500μL

TE buffer (10mM tris-HCL (pH=8.0), 1mM EDTA). Then DNA was eluted in 1% SDS, 100mM $NaHCO_3$. To reverse crosslinking, NaCl was added to a final concentration of 200mM and incubated at 95ºC for 15 min to reverse PFA cross-link. Afterwards, samples were incubated at 62ºC for 2h with Proteinase K, which was afterwards inactivated at 95ºC for 10 min. Finally, DNA was precipitated using 1/10 volume of 3M sodium acetate (pH=5.2), 2.5 volumes of absolute EtOH, and glycogen to 0,1 µg/µL. After 1h incubation at -20ºC, DNA was pelleted by centrifugation and resuspended in water. qPCR was performed to quantify DNA binding to Rps3 as explained earlier and normalized to mtDNA immunoprecipitation control without antibody (beads and lysate only).

Fiji Macro for quantification of DNA particles in the cytoplasm not colocalizing with H3

```
run("Duplicate...", "title=[KO 7] duplicate");
selectWindow("210119.lif - KO H3 7");
selectWindow("KO 7");
run("Split Channels");
selectWindow("210119.lif - KO H3 7");
selectWindow("C1-KO 7");
selectWindow("C2-KO 7");
selectWindow("C1-KO 7");
selectWindow("C3-KO 7");
selectWindow("C2-KO 7");
selectWindow("C4-KO 7");
selectWindow("C3-KO 7");
selectWindow("C4-KO 7");
selectWindow("C3-KO 7");
selectWindow("C2-KO 7");
selectWindow("C1-KO 7");
run("Gaussian Blur...", "sigma=15");
setOption("BlackBackground", false);
run("Convert to Mask");
run("Fill Holes");
//run("Threshold...");
//setThreshold(255, 255);
run("Convert to Mask");
run("Close");
run("Create Selection");
selectWindow("C2-KO 7");
run("Restore Selection");
setBackgroundColor(0, 0, 0);
run("Clear", "slice");
run("Duplicate...", "title=[mtDNA no nuclei]");
selectWindow("C2-KO 7");
selectWindow("mtDNA no nuclei");
run("Subtract...", "value=4");
run("FeatureJ Laplacian", "compute smoothing=3.0");
73
setAutoThreshold("Otsu dark");
//run("Threshold...");
run("Convert to Mask");
run("Close");
run("Analyze Particles...", "size=0.05-Infinity display include
summarize");
selectWindow("mtDNA no nuclei");
selectWindow("C1-KO 7");
selectWindow("C2-KO 7");
selectWindow("C3-KO 7");
run("Subtract...", "value=35");
run("Median...", "radius=5");
setAutoThreshold("Otsu dark");
//run("Threshold...");
//setThreshold(21, 255);
run("Convert to Mask");
run("Close");
run("Create Selection");
selectWindow("mtDNA no nuclei");
run("Duplicate...", "title=[mtDNA no nuclei no mito]");
run("Restore Selection");
run("Clear", "slice");
run("FeatureJ Laplacian", "compute smoothing=3.0");
setAutoThreshold("Otsu dark");
//run("Threshold...");
```

```
setThreshold(-1000000000000000000000000000000.0000, -1.0100);
run("Convert to Mask");
run("Analyze Particles...", "size=0.05-Infinity display include
summarize");
run("Close");
selectWindow("mtDNA no nuclei no mito");
selectWindow("mtDNA no nuclei");
selectWindow("C3-KO 7");
selectWindow("C4-KO 7");
run("Subtract...", "value=9");
run("Gaussian Blur...", "sigma=5");
run("FeatureJ Laplacian", "compute smoothing=3.0");
setAutoThreshold("Otsu dark");
//run("Threshold...");
run("Convert to Mask");
run("Create Selection");
selectWindow("mtDNA no nuclei no mito");
run("Duplicate...", "title=[mtDNA no nuclei no mito no H3]");
run("Restore Selection");
run("Clear", "slice");
run("Close");
run("FeatureJ Laplacian", "compute smoothing=3.0");
setAutoThreshold("Otsu dark");
//run("Threshold...");
setThreshold(-1000000000000000000000000000000.0000, -1.0100);
run("Convert to Mask");
run("Analyze Particles...", "size=0.05-Infinity display include
summarize");
```

FIJI script for Rps3/DNA/mitochondria, colocalized pixels

```
Remove pixels from nuclear area
//setTool("freehand");
run("Cut");
Determine rps3/DNA colocalized points
run("Split Channels");
run("Colocalization ", "channel_1=[200211_MEFs wt R3 5.tif (red=Rps3)] channel_2=[200211_MEFs wt R3 5.tif (blue=DNA)] ratio=50 threshold_channel_1=50 threshold_channel_2=50 display=255");
run("Split Channels");
run("Measure");
Determine pixels for total DNA staining
selectWindow("Colocalizated points (RGB)  (green)");
setOption("BlackBackground", true);
run("Make Binary");
run("Measure");
Determine triple positive pixels
run("Colocalization ", "channel_1=[Colocalizated points (RGB)  (blue= colocalized points)] channel_2=[200211_MEFs wt R3 5.tif (green = Hsp60)] ratio=50 threshold_channel_1=50 threshold_channel_2=50 display=255");
run("Split Channels");
run("Measure");
```

R Script: Differential Enrichment analysis in proteomics data from Ifnb-/- vs Ifnb+/+ EVs

```
1. Data preparation and differential expression analysis
# Open libraries
library(readxl)
library(ggplot2)
library(ggsignif)
library(dplyr)
```

```
library(tidyverse)
library(DEP)
library(stringr)
library(ggrepel)
library(cowplot)
library(DT)
library(knitr)
library(kableExtra)
library(DESeq2)
library(hexbin)
library(writexl)
# Import data
normdata <- read_excel("normabundancesforpseudoimp.xlsx")
# View(normdata)
data <- normdata
# Check for missing gene names (symbol): 1
sum(is.na(data$Symbol))
# Check for duplicated gene names (symbol): 23
sum(duplicated(data$Symbol))
# Make unique names using the annotation in the "Symbol" column as primary
names and the annotation in "Accession" as name for those that do not have
a gene name: we are adding 2 columns (name, symbol)
data_unique <- make_unique(data, "Symbol", "Accession", delim = ";")
data_unique_complete <- data_unique
# We add 2 columns to the dataset (1 with the number of missing values of
the KOs per row and 1 with the number of missing values of the WTs per row)
# Save the names of the rows (we need to get rid of columns because otherwise we
can't make the following steps)
rownames(data_unique) <- data_unique$name
#Eliminate the columns that have names or accession numbers
data_unique$Accession <- NULL
data_unique$Symbol <- NULL
data_unique$name <- NULL
data_unique$ID <- NULL
#Change the missing values (i.e. equal to 0) to "NA" (to be able to count
them after)
data_unique[data_unique == 0] <- NA
# Add back the names of the rows and accession
data_unique$name <- rownames(data_unique)
data_unique$ID <- data_unique_complete$ID
# Define the variables knock & wild to equal to the vector containing the
names of all the KOs and of all the WT, respectively.
KO <- c("KO_F1","KO_F2","KO_F3")
WT <- c("WT_F4","WT_F5","WT_F6")
# Create 2 columns with the count of the number of missing values per row
for each genotype
data_unique$NA_KO <- apply(data_unique[,KO], 1, function(x) sum(is.na(x)))
data_unique$NA_WT <- apply(data_unique[,WT], 1, function(x) sum(is.na(x)))
# Save table with columns indicating number of imputed values
write_xlsx(data_unique,"/Users/joanamarturia/Desktop/Untitled
folder/EVproteomicsJMN/210825protdata_numNAspercondition.xlsx")
#1.6 Substitute the missing values of each row of each genotype by 0.1 (sample
by sample)
data_unique$KO_F1 <- ifelse(is.na(data_unique$KO_F1), 1077,
data_unique$KO_F1)
data_unique$KO_F2 <- ifelse(is.na(data_unique$KO_F2), 1077,
data_unique$KO_F2)
data_unique$KO_F3 <- ifelse(is.na(data_unique$KO_F3), 1077,
data_unique$KO_F3)
data_unique$WT_F4 <- ifelse(is.na(data_unique$WT_F4), 1077,
data_unique$WT_F4)
data_unique$WT_F5 <- ifelse(is.na(data_unique$WT_F5), 1077,
```

```
data_unique$WT_F5)
data_unique$WT_F6 <- ifelse(is.na(data_unique$WT_F6), 1077,
data_unique$WT_F6)
protdatapseudocount <- data_unique
# Remove extra columns so we have name and ID
Clean_datapseuocount <- select(protdatapseudocount, -c("NA_KO", "NA_WT"))
# Reorder columns so we have name and symbol first. First get the names of
the columns assigned to numbers and we can call them by their position and
rearrange them.
colnames(Clean_datapseuocount)
data_pseudocount_ready <- Clean_datapseuocount[, c(8, 7, 1, 2, 3, 4, 5, 6)]
# Save table with pseudocount ready for DEP
write_xlsx(data_pseudocount_ready,"/Users/joanamarturia/Desktop/Untitled
folder/EVproteomicsJMN/210826data_pseudocount_1077_fordep.xlsx")
# Import .txt files
abundancepseudo <- read_tsv("210826data_pseudocount_1077_fordep.txt")
design <- read_tsv("design.txt")
# Generate a SummarizedExperiment object using an experimental design
data_columns <- grep("_", colnames(abundancepseudo))
data_se <- make_se(abundancepseudo, data_columns, design)
# Differential Enrichment Analysis. Test every sample against control
data_diff <- test_diff(data_se, type = "control", control = "WT")
# Denote significant proteins based on user defined cutoffs
data_dep <- add_rejections(data_diff, alpha = 0.05, lfc = log2(1.5))
# Generate a results table
results_dataprotpseudocount <- get_results(data_dep)
# Number of significant proteins: 287
results_dataprotpseudocount %>% filter(significant) %>% nrow()
#Generate data frame
df_results_pseudocount <- get_df_wide(data_dep)
view(df_results_pseudocount)
# Save results table as Excel
write_xlsx(df_results_pseudocount,"/Users/joanamarturia/Desktop/Untitled
folder/EVproteomicsJMN/210826results_pseudocount_1077_normabundance_notfilt
.xlsx")
2. Data Vidualization
2.1. Volcano Plot
# Import data: norm abundance, pseudocount 1077
data_287 <-
read_excel("210826results_pseudocount_1077_normabundance_notfilt.xlsx")
# Volcano plot with p-val (NOT ADJUSTED)
data_287$diffexpressed <- "NO"
data_287$diffexpressed[data_287$KO_vs_WT_diff > 0.6 &
data_287$KO_vs_WT_p.val < 0.05 ]<- "UP"
data_287$diffexpressed[data_287$KO_vs_WT_diff < -0.6 &
data_287$KO_vs_WT_p.val < 0.05 ]<- "DOWN"
data_287$name <- as.character(data_287$name)
data_287$protlabel <- NA
data_287$protlabel[data_287$diffexpressed == "UP" | data_287$diffexpressed
== "DOWN"] <- data_287$name[data_287$diffexpressed == "UP" |
data_287$diffexpressed == "DOWN" ]
p <- ggplot(data= data_287, aes(x=KO_vs_WT_diff, y=-log10(KO_vs_WT_p.val),
col=diffexpressed, label=protlabel)) + geom_point()+
geom_text_repel(max.overlaps=30, box.padding = 0.5, size=3,
segment.size=0.3)
p + geom_vline (xintercept = c(-0.6, 0.6), col= "black", linetype="dashed")
+
geom_hline (yintercept=-log10(0.05), col="grey", linetype="dashed") +
ylab("-Log10 pval") + xlab("Log2FC") + theme_bw()
2.2. Heatmap
heat287 <- read_excel("210830pseudocount_1077_only100of287forheatmap.xlsx")
# Make unique names using the annotation in the "name" column as primary
```

```
names and the annotation in "ID" as name for those that do not have a gene
name
heat287unique <- make_unique(heat287, "name", "ID", delim = ";")
rownames(heat287unique) <- heat287unique$
```

References

1 Li, L. *et al.* The structural changes of gray matter in Parkinson disease patients with mild cognitive impairments. *PLoS One* **17**, e0269787, doi:10.1371/journal.pone.0269787 (2022).

2 Pereira, J. B. *et al.* Initial cognitive decline is associated with cortical thinning in early Parkinson disease. *Neurology* **82**, 2017-2025, doi:10.1212/WNL.0000000000000483 (2014).

3 Stamper, C. *et al.* Neuronal gene expression correlates of Parkinson's disease with dementia. *Mov Disord* **23**, 1588-1595, doi:10.1002/mds.22184 (2008).

4 Magalhaes, J. *et al.* PIAS2-mediated blockade of IFN-beta signaling: a basis for sporadic Parkinson disease dementia. *Mol Psychiatry* **26**, 6083-6099, doi:10.1038/s41380-021-01207-w (2021).

5 Ejlerskov, P. *et al.* Lack of Neuronal IFN-beta-IFNAR Causes Lewy Body- and Parkinson's Disease-like Dementia. *Cell* **163**, 324-339, doi:10.1016/j.cell.2015.08.069 (2015).

6 Tresse, E. *et al.* IFN-beta rescues neurodegeneration by regulating mitochondrial fission via STAT5, PGAM5, and Drp1. *EMBO J* **40**, e106868, doi:10.15252/embj.2020106868 (2021).

7 Kim, M. J. *et al.* Neuronal loss in primary long-term cortical culture involves neurodegeneration-like cell death via calpain and p35 processing, but not developmental apoptosis or aging. *Exp Mol Med* **39**, 14-26, doi:10.1038/emm.2007.3 (2007).

8 Perez-Gonzalez, R. *et al.* A Method for Isolation of Extracellular Vesicles and Characterization of Exosomes from Brain Extracellular Space. *Methods Mol Biol* **1545**, 139-151, doi:10.1007/978-1-4939-6728-5_10 (2017).

9 Yakes, F. M. & Van Houten, B. Mitochondrial DNA damage is more extensive and persists longer than nuclear DNA damage in human cells following oxidative stress. *Proc Natl Acad Sci U S A* **94**, 514-519, doi:10.1073/pnas.94.2.514 (1997).

10 Eruslanov, E. & Kusmartsev, S. Identification of ROS using oxidized DCFDA and flow-cytometry. *Methods Mol Biol* **594**, 57-72, doi:10.1007/978-1-60761-411-1_4 (2010).

11 Wang, X. & Roper, M. G. Measurement of DCF fluorescence as a measure of reactive oxygen species in murine islets of Langerhans. *Anal Methods* **6**, 3019-3024, doi:10.1039/C4AY00288A (2014).

12 Ng, L. F. *et al.* The mitochondria-targeted antioxidant MitoQ extends lifespan and improves healthspan of a transgenic Caenorhabditis elegans model of Alzheimer disease. *Free Radic Biol Med* **71**, 390-401, doi:10.1016/j.freeradbiomed.2014.03.003 (2014).

13 Kim, N. C. *et al.* VCP is essential for mitochondrial quality control by PINK1/Parkin and this function is impaired by VCP mutations. *Neuron* **78**, 65-80, doi:10.1016/j.neuron.2013.02.029 (2013).

proteomic	TLR Ligand	mitochondrial genome maintenance	oxidized DNA binding
Fam20b	Ppp3r1	Tfam	Rps3
Vps4b	Gdf11	Polg1	Xrcc1
Nrcam	Apoe	Polg2	Ogg1
Grm5	Wars1	Ssbp1	Recql4
Adarb2	Hsp90b1	Vdac1	Blm
Psmb6	Npm1	Vdac2	Msh2
Glt8d1	Rps3	Vdac3	Mutyh
Tenm1	Hsp70	Rps3	Msh6
Slc23a2	Tril	Mpg	Wrn
Slc7a1	Fn1	Aag	Pot1a
Atp9a	Hmgb1	Mutyh	Pot1b
Hist1h2ak	Hsp60	Ung	
Gm216	App	Ogg1	
Sec31a	Tnc	Neil1	
Spag4	Vcan	Neil2	
Usp39	Bgn	Nthl1	
Hmgn5	Agt	Ape1	
Polr2f	BD-2	Pnkp	
Rnf213	Fgl1	Fen1	
Eif2b4	Habp2	Dna2	
Casc4	Ndst1	Exog	
Spryd7	Mrp8	Lig3	
Asah1	Mrp14	Parp1	
4933402P(	Olr1	Tdp1	
Ppp3r1	SAA-1	Aptx	
Fat4		Ybx2	
Sorcs3		Rad51	
Plod2		Rad51C	
Gdf11		Xrcc3	
Myeov2		Brca1	
Chst10		Tp53bp1	
Vat1l		53bp1	
Mbtps1		Rev3	
Eif4a2		Primpol	
Eif6		Chchd4	
Ppp6c		Mgme1	
Lgals3bp		Twnk	
Actr1b		peo1	
Sfn		TFB2M	
Prr11		mtTFB2	
Rufy3		Supv3l1	
Psmb1		PolB	
Cpxm1		Polrmt	
Dtd1		Apex1	
Mybbp1a		Atad3a	
Tpp1		Endog	
Rab4a		Mettl4	
Kdm1a		Mterf1a	
Uchl5		Mterf1b	

Ash2l
Rrp9
Ppp2r5e
Arf5
Strn3
Nup133
Rps19
Inhba
Lyn
Npm3
Pnn
Ctnnbl1
Polr2l
Fto
Apoe
Lsamp
Ilkap
Lphn2
Drap1
Cpsf1
Vps28
Bgn
Thoc6
Scube1
Anapc1
Sdc4
Polr2i
Ctsb
St3gal2
Htatsf1
Fst
Mfge8
Ywhaz
Calr
Apex1
Chst11
Tpp2
Igsf10
Psma1
Creg2
Wdr77
Rap2b
Slc35b4
B3gat2
Atp6ap2
Edil3
Cntn1
Snrpe
Rplp0
Psma6
Mterf2
Pif1
Poldip2
Polg
Polq
Ppa2
Recql4
Rnaseh1
Tfb2m
Top1mt
Top3a
Twnk
Cfh
Sesn2
Tk2
Rrm1
Rrm2b
Dnaja3
dpol-gamma_mouse
rrm1-rrm2b_mouse
Tp53
Atg7
Stox1
Mpv17
Ppargc1a
Flcn

Wars
Cnp
Mapre2
Sf3b4
Rpl18a
Psma7
Sf3a3
Gas1
Eif3f
Itgb1
Ly6h
Csnk2b
Aplp1
G3bp2
Mcat
Hnrnpc
P97798-2
Vcp
Snrpf
Rplp1
Psmd2
Rac2
Adam10
Pla2g7
Copb1
Polr2b
Clta
Rbbp4
Chmp5
Spon1
Carm1
Ppm1l
Ncl
Galnt15
Uba3
Col19a1
Psma5
Nedd4l
Rbbp7
Dctn1
Lrp4
Prkcsh
Gphn
Ntrk2
Rcn2
P4hb
Farp1
Hspa13
Ptma
St8sia4

Dkk3
Hist1h1b
Apoa1
Snrpb
Pdcd6ip
Dhx30
Cxadr
Itga6
Nlgn3
Cct7
Sf3a2
Snrpd1
Nptn
Sfpq
Adamts2
Rap2c
Eif3d
St8sia2
Sdcbp
Snrpd2
Psmb5
Snrpd3
Nlgn2
Grem2
Sf3b1
G3bp1
Psap
Clns1a
Phf5a
Polr2h
Atp1a2
Hyou1
Rpl13a
Psmd6
Atp6ap1
Polr2a
Cltb.1
Mgat4b
Ywhah
Hnrnpul1
Bai2
Por
Cpe
Rps5
Tmeff2
Hsp90aa1
Chuk
Snrpb2
Polr2j
Arpc3

Cd9
0610009D07Rik
Slitrk1
Ywhae
B4galnt4
Btbd17
Rpl7
Kars
Hsp90b1
Hnrnpd
Rpl14
Lpl
Chst15
Pdia4
Copg2
Calu
Ywhag
Clstn1.1
Tsg101
Prmt1
Psmd1
Itm2b
Rpl18
Cd47
Igsf8
Rpl11
Tenm2
Uxs1
Ywhaq
Psma2
Slc1a4
Ppm1g
Copg
Prmt8
H1f0
Rangap1
Bsg
Rpl35
Ncdn
Gpm6a
Hnrnpl
Hnrnpu
Ganab
Psmb4
Ssb
Tomm70a
Actr1a
Dlgap4
Pcyox1
Hsp90ab1

Nxph3
Cct4
Ncam1
Acaca
Cct6a
Prpf4
Cct3
Uso1
Psme2
Sdha
Nap1l1
Plrg1
Zbtb43
Sf3a1
Grwd1
Dync1i2
Hdac2
Calm3
Usp9x
Ppp2cb
Polr1c
Hbb-b1
Hist1h1e
Psmc5
Lrfn5
Rps6-ps4
Psmd12
Stambp
Npm1
Xylt1
Cops2
Rpl35a-ps4
Nutf2
Nop58
Tspan6
Nap1l4
Alb
Prpf8
Psmc4
Ncan
Gmpr2
Pdia6
Eif3h
Cltc
Srp68
Caprin1
Cdh13
Rpsa
Rps3
Strn

Ruvbl1
Tcp1
Kpna3
Ywhab
Cct5
Vps37b
Chd4
BC005764
Cct8
Ilf3
Rpl9
Trove2
St8sia1
Iars
Lgi2
Ptprs
Sf3b3
Itih5
Rab2a
Hist1h2af
Bzw1
Cadm1
Cd81
Kpna4
Trim47
Nlgn4l
Kpnb1
Rtn4
D10Wsu52e
Map1b
Cdc23
Met
Psmd8
Ctsd
Rps2
Cops4
Lsm2
Vprbp
Hnrnpk.1
Slc3a2
Psmd13
Rap2a
Abat
2610301G19Rik
Pgm1
Dnm1l
Rps7
Rps14
Ptgfrn
Rpl3

Qdpr
Gal3st3
Sae1
Huwe1
Fstl4
Farsb
Nhp2l1
Cdc16
Crlf1
Cct2
Polr2e
Marcksl1
Ruvbl2
Eif3b
App
Vangl2
Rps18
Hgs
Ist1
Ggh
Eif3a
Supt6
Nrd1
Psmd11
Hist2h2bb
Ipo7
Hspa4
Ppp1ca
Cmas
Cbln2
Cst3
Lphn1
Car11
Gna11
Rps16-ps2
Trim28
Dhx9
Rps11
Rps15a
Sars
Pcdha4-g
Pcdh8
Dctn5
Eif4a3
Astn1
Man2a2
Cyb5r3
Wdr82
Fam125b
Mars

Eftud2
Camk2b
Anapc4
Dnaja2
Nasp
Ap3m1
D630045J12Rik
Copb2
Eif2s1
Grik2
Scrn1
Dnm1
Polr2c
Dctn3
Atp6v1b2
Abca1
Rab4b
Atl1
Rps27a
Atp1b2
Wdr61
Prrt4
Eif3l
Dpysl2
Eef1b2
Sept5
Gars
Rpl22
Dnaja1
Snrpa1
Tsta3
Snrnp70
Ptpra
Aprt
Psmc3
Dpysl3
Aars
Oxct1
Arl8b
Psma3
Hist1h1d
Mdh2
Nucks1
Hs3st1
Fscn1
Clu
Gnb2l1
Eif2s3x
Cs
Crmp1

Ube2r2
Grm3
Atp5b
Syn3
LOC100044829
Dpysl5
Acot7
Hist1h2ae
Cul5
Actb
Ddx17
Add1
Uba1
Prkar2b
Dkc1
Hnrnpul2
Strap
Atp6v1a
Mmp24
Ilf2
Hspa8
Chl1
Hspa1a
Csf1
Dars
Mgrn1
Lrrc55
Scamp1
Hnrnpll
Ptprd
Dctn2
Tubb3
Mapk3
Sar1a
Eef1g
Mta1
Osbpl8
Thop1
Tmeff1
LOC100045999
Rbm12
Rpl10a
Dync1h1
Pcsk2
St3gal1
Canx
Rpl17
Rpl12
Rps17
Stmn3

Pa2g4
Nmt1
Gapdhs
Prl7c1
Fermt2
Psmb7
Rbx1
Stam
Supt16
Srp19
Snrpg
Chst1
Mif
Rplp2
Hba-a1
Ddx3x
Ppp2r2a
Efnb3
Gipc2
Eif4g2
Tubb4a
Thy1
Fam20c
Dync1li1
Rps9
Ptges3
Bub3
Syt11
Vcam1
Rnmt
Gtf2a2
1190002N15Rik
Cand1
Dnm2
Prps1
Elavl3
Rcor3
Efnb2
Psmd7
Eif3i
Eif3m
B3gnt5
Eif3c
Eif1ax
Scg3
Ldhb
Actr2
Asns
Psme3
Tnik

Rpl4
Rpl15
Ptprf
Ogt
Rab9
Col4a1
Synj2
Vdac1
Map3k3
Lphn3
Gpm6b
Rab13
Ttn
Atp1a1
Vps4a
Nop56
Chl1.1
Wscd1
Gsk3a
Dock7
Rfx5
Xpo1
Mta2
Wnt7b
Eif3k
Aplp2
Chmp3
Hspa9
Ttyh3
Dnajc5
Prom1
Gfra1
Bag6
Prpf19
Hnrnpa3
Ddx5
Syt4
Stmn2
Myl6
Sept6
Actg1
Copz1
Slc8a1
Fam171a2
Nans
Smarcd1
Eif3e
Serpinc1
Ankfy1
Ddx41

Ppa1
Dip2b
Ctbp1
Gpc1
Zfp326
Lrfn1
Ppp3ca
Rpl32
Slc6a1
St6galnac5
Pdxk
Rpl38
Actl6a
Sept11
Slc1a3
Acly
Lama5
Nog
Chmp2a
Pygb
Rab6b
Ssrp1
Nono
Atp2a2
Arpc1a
Gps1
Psmd3
Pgrmc1
Yars
Naa10
Gnl1
Atxn10
Prpf4b
Ddb1
Trim2
B4galt2
Zfp28
Ranbp9
Slc44a2
Sat2
Supt5
Gan
Cpsf6
Spr
Atat1
Zcchc8
Plxna2
Cope
Dis3
Pak1ip1

Smarcd3
Lrfn2
Brcc3
Anapc7
Tpst1
Dpy30
Tpm3
Rab5b
Polr2k
Prmt3
Pvrl1
Mfap1a
Gdpd1
Lmnb1
Prdx4
Cdc27
Tsku
Acrv1
Adnp
Ptprk
Atp6v1h
Cadps
Ankrd46
Plch1
Xrn2
B3gat1
Abce1
Fbxo22
Srp14
Dpp6
Bre
Mblac2
Sephs2
Col5a1
Thoc1
Smarce1
Cops8
Psmd5
Ppp4c
Vsnl1
Numbl
Tspan5
Dag1
Mbd3
Psme4
Lamp2
Abhd15
Gid8
Aimp2
Ube2n

Ubtf
Dynll2
Gria4
Fgfbp3
Eef1e1
Tpm4
Thoc2
Dhx15
Rbbp5
Sfrp2
Rwdd1
Glmn
Man2b1
Akr7a5
Chchd8
Dis3l
Pddc1
Lap3
Srrm2
Tubb2b
Vps37c
Tbca
Ptpn5
Sulf1
Lamtor1
Ttr
Npdc1
Cttn
Gclm
Akr1e1
Ddx39b
Macrod2
Rin2
Tspan14
Pum2
Psmb2
Nmt2
Eif4g1
Cdc26
Lsm7
Sdccag3
Tbc1d24
Ndufv1
Celf2
Dhrs11
Ppp2r2d
Scube3
Mat2a
Hmgb1
Otud7a

Trim32
Pbx1
Synj1
Akap8l
Cadm2
Sema6d
Atp1a3
Ggps1
Fgfr1
Vbp1
Trmt5
C1qc
Snca
Igfbpl1
Nup43
Prpf31
Lancl1
Tspan18
Mcts1
Aatf
Cryz
Rpl26
Psph
Nt5c
Tbc1d13
Commd9
Dscaml1
Safb
Col11a1
Prpsap2
Sart3
Dtx1
Usp4
Pkm.1
Fbxl16
Cd63
Gpc2
Chst2
Cltb
Nrbp1
Khsrp
Sh3pxd2b
Islr
Arpc5
Rpl5
Ctnnd2
Med1
Tubb4b
Psmd4
Hspa5

Ncald
Ptprt
Farsa
Spnb1
Mob4
Gpx4
Ctsz
Scg5
Capn5
Pcyt2
Ndufa4
Ube4b
2310035C23Rik
Adrm1
Lrp1
Ttyh1
Mta3
Rps27
Arpc4
Kif21b
Igsf3
Psmg3
Hnrnpm
Ola1
Dctn4
Actl6b
Bcan
Sept7
Nrxn1
Fah
Ftl1
Eif2s2
Actr3
Fam69b
Dcaf5
Gabra3
Eef2
Rtn3
Khdrbs1
Fasn
Ago1
Eef1a2
Cyfip1
Rpl21
Xpo7
Idh3a
Mdh1
Ins1
Aspm
Swi5

Tkt
Tuba4a
U2af1
Snd1
Egflam
Myh10
Csnk2a1
Capza2
Ube2m
Apoa1bp
Rpl30
Rab5c
Epm2aip1
Efnb1
Fn1
Sparcl1
Acta1
Prelp
Gart
Cx3cl1
Eprs
Coro1a
Ptpro
Smarcc2
Sf3b5
Atp6v1e1
Strbp
Pcsk1
Snf8
Ppp2r1a
Lrrc47
Vars
St8sia3
Serpine2
Cbln1
Mthfd1
Etf1
Hist4h4
Psmc2
Ap2a1
Tmem198
Slc4a4
Rpl10
Masp1
Lrrn1
Chga
Macf1
Cdc42
Cspg4
Pkm

Sept9
Adk
Snap25
Rab3a
Pcdha9
C1qb
Brox
Zfml
Rpl24
Cpne8
Cp
Vat1
Tubb5
Ciita
Tceb1
Usp5
Sema3c
Tuba1a
Anxa7
Matr3
Acadm
Rps26
Hpd
Fh1
Vamp2
Rps4x
Taldo1
Syn1
Tom1
Esm1
Lrrc4b
Gpr56
Ptp4a2
Fbll1
Htra1
Dazap1
Nlgn1
Purg
Rps13
Ehd1
Vapb
Tollip
Ndrg4
Draxin
Ddx6
Rps12
Rpl13
Rap1a
Tril
Gpc5

Rab11b
Rab6a
Tubb2a
Cnrip1
Plod3
Rap1b
Chadl
Sept3
Krt15
Cbr1
Gdi1
Rpl28
Vps29
Ppp1cc
Gm1673
Ssbp3
Spock1
Prdx1
Spna2
Hnrnpa2b1
Nrxn3
Clic1
Med13l
Nxph1
Vps35
Rpl27a
Mapre1
Psmc1
Rab8a
Celsr2
Rrp1
Hmgcs1
Acaa1b
Psma4
Gsk3b
Hs6st2
Ccbe1
Rab1
Nrp2
Ptk7
Hist2h2ac
Fstl1
Hs6st1
Hp1bp3
Rab7
Rtn4rl2
Prkacb
Rab1b
Mapt
Epb4.1l1

Acat1
Gapdh
Rps15
Cacna2d2
Cap1
Add2
Skp1a
Prrc2b
Atxn2l
Pgm2
Islr2
Paf1
Ppm1e
Arf3
Vapa
Cacna2d1
Adh5
Mbtd1
Mvd
Lgi1
Rab14
Top2b
Actr10
Neto2
Fgfr3
Iws1
Pdgfra
Uba2
Prkar2a
Cdc42.1
Bmpr2
Gm13826
Gstp1
Purb
Casp3
Ap3b2
Pspc1
Prss23
Anxa6
Stx1b
Arl15
Nrcam.2
Ptprz1
Arpc1b
Erbb4
Dpysl4
Fbln2
Cul2
Plxdc2
Camta1

Rabl3
Glo1
Naa15
Prdx2
Scrg1
B3galt1
Nfib
Plekhb2
Ndrg2
Ssbp1
Copa
Ptms
Mdm4
Clstn1
Eif4e
Arpc2
Prmt5
Dbi
Gar1
Rpl6
Plxna3
Rpl34-ps1
Krt76
Rps3a
Flna
Grlf1
Adprh
Pnp
Grin1
Psmd14
Usp14
Mgea5
Esd
Rpl19
Sez6l2
Gprc5b
Pgam1
Hint1
Flot1
Nell2
Hist3h2ba
Scg2
Slc25a23
Cd200
Upb1
Serpina3n
Clcn4-2
Ccar1
Rpl31
Trim67

Rpl23
Pcbp2
Smarca4
Rars
Eno1
Rcc1
Fbxo2
Elavl1
Atp1b1
Vps37d
Ppp1r7
Lama2
Camk2a
Krt10
Lss
Acss3
Sst
Nars
Itm2c
Smoc1
Hcfc1
Rpl27
Atp6v1g1
Rbm39
Rpp30
Prnp
Hmgb2
Fyn
Trim33
Akr1b3
Gmps
Otub1
Rps20
Eva1a
Drg1
Tpi1
Tceb2
Ppp2ca
Acnat2
Hist2h2be
H2afv
Arl3
Usp7
Capza1
Fn3krp
Rpl37a
Emid1
Spp1
Cops6
D3Bwg0562e

Dpyd
Anxa2
Rps23
Hepacam
Leo1
Col7a1
C1qa
Cops5
Gpc6
Dis3l2
Stx12
Xpr1
Gabra2
Aldh9a1
Pygl
H2afz
Dynll1
Eef1d.1
Rac3
Nsg1
Sgta
3110003A17Rik
Myh9
Ipo5
Arcn1
Plxna4
Gspt1
Flot2
Hist1h3i
Tspan9
Nxph2
Hnrnpdl
Hapln1
Vps36
Psmc6
Pcca
Snrnp40
Rab21
Luzp2
Xylt2
Gmpr
Ngdn
Dynlt1b
Eef1a1
Negr1
Eif5
Hist1h1c
Rpl8
Dbn1
Aldoa

Nrcam.1
Marcks
Mtpn
Ntm
Capn2
Aldh1l1
Sfrp1
Abi1
Hnrnph1
Ddx1
Rala
Mag
Aco1
Rpl7a
Pafah1b1
Lypla2
Rps8
Ank2
Lsm12
Anp32a
Luc7l3
Ndrg3
Pcdhb14
Tars
Ppp1cb
Camk2g
Acan
Fkbp4
Ranbp1
2610002M06Rik
Tfrc
Epha5
Plxna1
Ldha
Anp32e
Fras1
Pgk1
Hspa2
Sclt1
Ncbp1
Syncrip
Wdr1
Dpys
Capzb
Tnr
Cyfip2
Atic
Cse1l
Cops3
Map1lc3a

Ndufa6
Ids
Elfn2
Rab18
Rab10
Acat2
Txnl1
Rps24
Pcsk1n
Rps25
Alad
Fam78a
Thrap3
Ptn
Cd151
Syn2
Psip1
Eif5a
Hnrnpab
C3
Dstn
Pfn2
Vwa1
Snx27
Cbln4
Brk1
Olfm2
Serinc5
Podn
Rp2h
Ak1
Pebp1
Ech1
Aifm1
Nrxn1.1
Sec23ip
2410066E13Rik
Sri
Gria2
Papss1
Eif4a1
Map4k4
Lrrn3
Aldob
Asna1
Angpt1
Gstm1
Suclg2
Asl
Pcdhga4

Fam19a1
Ssc5d
Uchl1
Adam17
Aldh1a1
Bhmt
Myof
Syt1
Src
Rcc2
Mapre3
Rgs18
Plekhj1
Robo2
Ap2s1
Agrn
Krt5
Prpf40a
Cbx3
Atp5a1
Nbea
Api5
Nup85
Rpia
Krt79
Reln
Basp1
Cpsf7
Ndp
Asap2
Nop2
Krt16
Larp4b
Pgm2l1
Srsf10
Nae1
Wdr26
Krt75
Cadm3
Bcat1
Arhgdia
Sparc
Dnajb11
Lsm6
Krt17
Wdr13
Chm
Sltm
Srrm1
Vim

Sprtn
Hnrnpk
Atp6v0d1
Peli2
Dmxl2
Amph
Eci2
Stx7
Bmpr1a
Tra2a
Pacs2
Ly6e
Zc3h18
Usp10
M6pr
Atp6v0a1
Tssc1
Ptpn1
Qtrt1
Gabrg2
Ddx46
Cspg5
Sult4a1
Arid1a
Akr1a1
Polr2g
Uap1l1
Utp18
Lage3
Stx6
Polr1d
Ppp1r9b
Arf1
Anapc11
Map2
Smu1
Syngap1
Gpd1l
Igf2r
Stx1a
Krt19
Tbcb
Pcna
Mtmr2
Fsd1l
Wtap
Vit
Tgm2
Ptpn23
Adcyap1r1

Akt1s1
Ednrb
Nudt16l1
Sh3gl2
Wdr43
Birc6
Ube2z
Fmnl2
AI314180
Katnb1
Stc2
Rbp1
Usp15
Rhoc
Rab8b
Env
Slc4a8
Dpp3
Mrto4
Tmsb4x
Napg
Slc6a9
Bai3
Trappc6b
Cstf1
Rab3c
Cdc42bpb
Mn1
Appl1
Alpl
1500003O03Rik
Cacna2d3
Lxn
1110037F02Rik
Acadsb
Nudcd2
Srp54a
Ostn
Ndrg1
Tspan13
Fech
Polr3d
Srpk2
Atrn
Scpep1
Dlg3
Atp6v1c1
Capn7
Pold1
Mib1

Tmem176b
Smchd1
Plxnc1
Lipg
Tenm4
Zmym3
Srpk1
Cdk7
Hpx
Fahd2a
Dcx
Rgn
Chmp2b
Kctd12
Trip12
Myl9
Dlg4
Gng2
Grin2b
Sept2
Clstn3
Alcam
Ethe1
Ndnf
Olfml2b
Mapk1
Rnasel
Kif5c
Npepps
Gemin5
Gpc4
Rac1
Ap2a2
Nsg2
Vac14
Epb4.1l3
Prune2
Myo18a
Cask
Seh1l
Hist1h3d
Pafah1b2
Napa
Sap18
Wdr33
Krt73
Hbb-y
Ppp2r5c
Gstm2
Wdr47

Strn4
Srgap3
Gnai3
Carkd
Eef1d
Mfap2
Ppap2b
Dhrs4
Atxn2
Hmgb3
Txn1
Hspd1
Gja1
Matn2
Krt42
Slc1a2
Thoc7
Fam19a2
Plxnb2
Lypla1
Sepp1
Ddah2
Dcn
Lrfn4
Itih2
Cant1
Arhgap23
Pfdn5
Gnb4
Stip1
Ephb3
Ptx3
Gdi2
Lgals1
Btf3
Ap2m1
Rps10
Tcf7
Ttll12
Rpl36
Ephb2
Ostf1
Pafah1b3
Rpl23a
Hsph1
Srsf2
Rab31
Gpi1
Pik3c3
Spock2

Ttl
Rmnd1
Srsf5
Bcas2
Chgb
Snx3
Atp6v1c2
Cacybp
Gnaq
Tenm3
Spock3
Arhgap1
Adam22
Nptx2
Fli1
Glyr1
Fam98b
Stmn1
Fjx1
Myo5a
Map1a
Luc7l2
Ntn1
Wdr37
Phyhipl
Decr2
Mdk
Kras
Sh3gl1
Phgdh
Mpst
Anapc5
Naa50
Emilin1
Dner
Kpna1
Mtap
Epb4.1
Trf
Magee1
Agxt2
Rhoa
Emilin2
Csnk1g1
Nop10
Dync1li2
Apobr
Hnrnpa1
2810405K02Rik
Idi1

Spnb2
Ctnna1
Hsd17b10
Cmpk1
Pon1
Ap2b1
Zc3hc1
Dnm3
Slc6a11
Dclk1
Psmb3
Bzw2
Snrnp200
Acacb
Anapc2
Abhd14b
Arg1
Hist2h2ab
Coro1c
Dcaf7
Dbnl
Nudt11
Atrnl1
Olfr147
Mras
Vti1b
Mia3
Cndp2
Git1
Fam125a
Lars
Selenbp2
Paics
Gnb1
Elmod1
Sdf4
S100a4
Vta1
Dcxr
Prkar1a
Dtx4
Ctnnb1
Psd3
Hgd
Proser1
Pabpc1
Dak
Gng12
Blmh
B4galt3

Cmip
Gnb2
Dmgdh
Cdk5
Clstn2
Chordc1
Acp1
Crym
Mrps25
C1qtnf4
Slc39a10
Hsd17b4
Pip4k2b
Glul
Ugdh
Tnn
Gcat
Tra2b
Actn4
9930013L23Rik
Aebp1
Ubr4
H2afx
Cotl1
Hist1h1a
Vps33a
Kif5b
Glg1
Ppme1
Pcbp1
Stmn4
Lama1
Efcab10
Fgfrl1
Cecr6
Gria1
Hnrnpa0
Cenpv
Sulf2
Sez6
Mecp2
Notch1
Srsf3
Sec13
Gap43
U2af2
H2afy2
Stam2
Ube3a
Tspan7

Rtn1
Kif1b
Tnc
Tiprl
Alpk1
Prkce
Slit2
Atp7a
Cfdp1
Ahcy
Idh1
Mvp
Drg2
Galnt2
Ahcyl1
Chmp4b
Aldh2
Srm
Vtn
Prpsap1
Pfkm
Akap8
Prkar1b
Gna13
Isoc1
Acvr1b
C2cd4d
Ppid
Jup
Tnpo2
St13
Hspb1
Glud1
Col18a1
Aox1
Pgd
Ybx1
Gnao1.1
Pacsin2
Aldh8a1
Kcnd3
Acy1
Hapln3
Hs6st3
Krt1
Hdgfrp3
Tmem132a
Gnai2
Rab5a
Acads

Lrp6
Top1
Klc2
Itfg1
Wbp2
Vcan
Set
Cps1
Ier5l
Dchs1
Thoc5
Acin1
Lamb2
Ckap5
Stxbp1
Arpc5l
Krt14
Srsf1
Fam49a
Kif13a
Aco2
Col2a1
Aldh1a7
Otc
Srsf7
Nlrp4f
Nras
Trim3
Pcdhb18
Sms
Rpl36al
Clic4
Ctnnd1
Nsf
Gpc3
Itpa
Naprt1
Tor2a
Ppia
Fbp1
Psat1
Gnao1
2310016M24Rik
Luc7l
Fgfr2
Cpd
Set.1
Keg1
Ezr
Atp6v1d

Dcps
Srrt
Gcdh
Fam169a
Bmp1
Trio
Aldoc
Fabp5
Rab35
Nme2
Slit1
Sez6l
Ap3d1
Bin1
Taok1
Cbx1
Cul4a
Oat
Tgfb2
Mapk10
Ube2o
Car10
N28178
C4b
Upf1
S100a11
Ctps
Pklr
Mgat5
Bclaf1
Rnf215
Prdx6
Ankrd28
Igfbp5
Col12a1
Syt5
Acaa2
Dek
Bmper
Nrp1
Atp2b2
Nell1
Chmp1b
Tardbp
Arl6ip5
Smarcb1
H2afy
Serpini1
Scn4a
Inpp4a

C1qbp
Ctnna2
Tpt1
Echs1
Notch3
Uchl3
Pop4
Klc1
Vps25
H3f3a
Daam1
Ap3s1
Eef2k
Fabp7
Napb
Decr1
Ablim1
Kit
Cspg5.1
Nfia
Gsto1
Sep15
Ddah1
Cbx5
Pfn1
Nid1
Cxcl14
Cfl1
Cdc37l1
Dsp
Epha4
Adsl
Pmpca
Atp2b1
Dnajc6
Akt3
Pura
Aarsd1
Col26a1
Top3b
Thra
Mfap4
Nid2
Nagk
Krt2
Hspg2
Galnt16
Lsm4
Pgls
Egfr

Aldh7a1
Phlpp1
Gyk
Naca
Trmt6
Hoga1
Wdfy1
Gnas
Pacs1
Lamc1
Rsc1a1
Skiv2l2
Ckb
Pccb
L1cam
Fmod
Sdc3
Cfl2
Col4a2
Cd44
Ptpn11
Dip2a
Wdr65
Sar1b
1190003J15Rik
Cort
Cacnb3
Casz1
Lama4
Plxnb1
Cat
Vstm2l
Podxl2
Snrpa
Park7
Ntrk3
Exosc2
Rtca
Epb4.1l2
Wdr5
Idh2
Tubb6
Cox6a1
Hkdc1
Sub1
Gatm
Ube2v1
Ube2d1
Impdh1
Slfn5

Grcc10
Bfsp2
Ctr9
Banf1
Cdh2
Antxr1
Vcan.1
Pmvk
Tln2
Snx18
Wdr48
Rrm1
Pak3
Lin7c
Arl2
Wnt5a
Sqstm1
Pea15a
Prep
Rhob
Ptpn12
Slc9a3r1
Olfm1
Heatr7b2
Lamb1
Eif5b
Pacsin3
Zhx1
Smpd3
Apaf1
Ctps2
Fut8
Adhfe1
Ryr3
Ccdc93
Tsc22d1
Commd3
Tsen34
Ext1
Crcp
Gstm5
Mat2b
Mink1
2310057M21Rik
Arvcf
Prps2
Trp53bp1
Olig1
Dhx57
Ppp4r2

Tyro3
Pik3r1
Prepl
Ppargc1b
Cntfr
Cdc37
Hhla1
Prkcb
Fam171b
Cpeb2
Pan2
Stom
Cdh6
Man1a
Braf
Atr
Tnpo1
Bccip
Mpc2
Cdc5l
Tprgl
Psmg2
Prune
Cybb
Pdgfrb
Cdh11
Nhp2
Fbln1
Trmt1
Acss2
Aimp1
Phpt1
Thoc3
Samhd1
Got2
Pfdn1
Pygm
Disp2
Fam129b
Crnkl1
Arhgef7
Timp3
Emg1
Ppfia2
Hcls1
Acvr2a
Lamp1
Commd7
Atp2b4
Ehbp1

Sirt2
Nudt2
Llgl1
Maea
Gabra5
Crip2
Stx16
Lgalsl
Igf2
Slc38a3
Zfp91
Cnbp
Anp32b
Aacs
Wasf3
Lmo4
Acot13
Nup155
Lman1
Thsd7a
Pnkp
Arf4
Wnt5b
1110012J17Rik
Pnpla6
Pdcd6
Dusp3
Pogz
Tc2n
Alg8
Atp6v1g2
Pwp1
Hexa
Fat3
Ndfip2
Galc
1300001I01Rik
Apba1
Pdhb
Gba
Ubash3b
Pwp2
Dab1
Nprl2
Lmtk3
Nrxn1.2
Qars
Olfml3
Celf1
Terf2

Shmt1
Rprd2
BC018242
Bzrap1
Afap1l2
Urod
Trmt61a
Fsd1
Kif2a
Arglu1
App.1
Dhrs1
Clcf1
Itgav
Fmr1
2310021P13Rik
Eppk1
Tubgcp4
Lmna
Crx
Glipr2
Ctbp2
Shprh
U2surp
Asph
Hectd3
Phf6
Polr3e
Cherp
Ranbp10
Cd99l2
Zdbf2
Ccdc22
Cacng4
Nup107
Pcsk5
Gtf3c1
Adss
Gabpa
Tomm34
Rap1gap
Plek
Arrb1
Prrt3
Rgma
Dus4l
Coro7
Ehd3
Hdgf
Otud4

Baz1a
C1ql1
Slitrk2
Disp2.1
Usp26
Cnot10
Hnrnpc.1
Unc119
Sdpr
Uroc1
Nadk2
Clic5
Exosc7
Denr
Avl9
Uba5
Ddr1
Eif2b5
Nploc4
Ppm1d
Cobra1
Coro2b
Unc5c
Ghitm
Shoc2
Scfd2
Narg2
Hmgcl
Vps53
Grn
Gng5
Grip1
Epha3
Atp4a
Atrx
Mrps11
Amigo3
Col9a1
Polr2d
Rheb
Xpnpep1
Osbpl11
Suv420h1
Ndufaf1
Satb2
Cops7b
Cad
Gng3
Rnf130
Nfe2l2

Agrn.1
Ptges2
Hdac1
Evl
Git2
Plod1
Tgfbr3
Ttc24
Aox4
Dpp8
1700047I17Rik2
Cnot1
Snx6
Tubg1
Tmem130
Msx1
Smarca2
Dcaf11
Pfas
Mtmr7
Atl2
Adarb1
Prrt1
Faim2
Calml3
Gabbr2
4930506M07Rik
Trim47.1
Klhdc4
Smarca5
E130309F12Rik
Atp1b3
Immt
H2afy.1
Actr3b
2810459M11Rik
Akt1
Cbs
Nmral1
Fkbp1a
Ptch1
Cdip1
Zranb2
Tubb1
Gnb5
Dlg2
Clip3
Plat
Pdgfc
Rhog

Ptprj
Cd2bp2
Mzt1
Hip1
Unc79
Ppp6r3
Cxcl12
Fam120a
Wnt7a
Ptpru
Nat10
Exosc5
Entpd2
Creg1
Abhd17c
D6Wsu163e
Ecm2
Ppp6r1
Rrm2b
Memo1
Polr3b
Cct6b
Usp34
Uba6
Xirp2
Rnaset2a
Amdhd1
Tnc.1
Rnf123
Obfc1
Srsf4
Magohb
Gaa
Dhx36
Tppp3
Nod1
Ube2i
Tuba1c
Ppt1
2700060E02Rik
Eif3j1
Trp53rk
Cdh23
Snx33
Rbm22
Cops7a
Iah1
Mrgpra5
Vps18
Pan3

Xpot
Inhbb
Msn
Scyl2
Glrx3
Shroom2
Btf3l4
Pld2
Fth1
Lhfpl4
Gabrb3
Tmem67
Pam
Slc4a7
AK010878
Kif3b
Akirin1
Serpinf1
Serpinb6a
Gmppa
Metap1
Mgat5b
Tagln2
4632415K11Rik
Robo1
Med20
Gamt
Prr15
Tax1bp1
Gpx1
Atp2c1
Naa11
Wasf1
Hao1
Erh
Ube3b
G3bp2.1
Map2k1
Prdx5
Pld3
8430419L09Rik
Irf2bpl
Ipo9
Serpinh1
Grhpr
Thbs4
Nt5c3b
Exoc4
Epha10
Setd7

Ptprn2
Acot4
Notch2
Kat2b
Nudt5
Paox
Gpsm2
Ankrd17
Ptrh2
Fip1l1
Uty
Nt5c2
Ppp2r5b
Camk2d
Crybb1
Dpp10
Gabrr1
6030458C11Rik
D130043K22Rik
Ntn4
Zzef1
Plcg1
Fntb
Cep290
Pkdcc
Mbd3l1
Vdac3
Vrk1
Nup93
Lancl2
Ccdc81
Wdr59
